# Proteogenomic Analysis of Protein Sequence Alterations in Breast Cancer Cells

**DOI:** 10.1038/s41598-019-46897-z

**Published:** 2019-07-17

**Authors:** Iulia M. Lazar, Arba Karcini, Shreya Ahuja, Carly Estrada-Palma

**Affiliations:** 1 Department of Biological Sciences, Virginia Tech 1981 Kraft Drive, Blacksburg, VA 24061 USA; 2 Carilion School of Medicine and Virginia Tech 1981 Kraft Drive, Blacksburg, VA 24061 USA; 3 Department of Biochemistry, Virginia Tech 1981 Kraft Drive, Blacksburg, VA 24061 USA

**Keywords:** Breast cancer, Mass spectrometry

## Abstract

Cancer evolves as a result of an accumulation of mutations and chromosomal aberrations. Developments in sequencing technologies have enabled the discovery and cataloguing of millions of such mutations. The identification of protein-level alterations, typically by using reversed-phase protein arrays or mass spectrometry, has lagged, however, behind gene and transcript-level observations. In this study, we report the use of mass spectrometry for detecting the presence of mutations-missense, indels and frame shifts-in MCF7 and SKBR3 breast cancer, and non-tumorigenic MCF10A cells. The mutations were identified by expanding the database search process of raw mass spectrometry files by including an in-house built database of mutated peptides (XMAn-v1) that complemented a minimally redundant, canonical database of *Homo sapiens* proteins. The work resulted in the identification of nearly 300 mutated peptide sequences, of which ~50 were characterized by quality tandem mass spectra. We describe the criteria that were used to select the mutated peptide sequences, evaluate the parameters that characterized these peptides, and assess the artifacts that could have led to false peptide identifications. Further, we discuss the functional domains and biological processes that may be impacted by the observed peptide alterations, and how protein-level detection can support the efforts of identifying cancer driving mutations and genes. Mass spectrometry data are available via ProteomeXchange with identifier PXD014458.

## Introduction

Cancer develops as a result of genetic alterations that include mutations and chromosomal abnormalities. Mutations include alterations such as single nucleotide substitutions (missense, nonsense and silent) and small insertions, deletions or frameshifts. Chromosomal abnormalities can be numerical, i.e., manifest themselves either as gains or losses in chromosome numbers (aneuploidy), or structural, i.e., lead to rearrangements such as translocations, inversions, deletions, insertions or duplications. Comprehensive high-throughput sequencing efforts have revealed a complex interplay of aberrations that define the heterogeneous nature of cancer genomes.

Earlier studies have identified ~140 genes that drive tumorigenesis, when altered by mutations^1^. These genes regulate three essential cellular processes through twelve signaling pathways, i.e., genome maintenance, cell survival and cell fate. Depending on type, tumors can harbor from 1–2 to 8 driver mutations that generally contribute to the development of cancer over 20–30 years in adults^1^. The field of cancer genomics, supported by the advance of sequencing technologies, has witnessed, however, a remarkable growth in the past decade, leading to joint multi-institutional efforts aimed at creating comprehensive resources that encompass hundreds to thousands of gene alterations with potential diagnostic and prognostic significance. Some of these resources include the Wellcome Sanger Institute’s COSMIC (Catalogue of Somatic Mutations in Cancer) and CGC (Cancer Gene Census) databases^2^, the cancer-specific projects supported by the International Cancer Genome Consortium (ICGC)^3^, the Cancer Cell Line Encyclopedia (CCLE)^4^, and the PanCancer Atlas project conducted by The Cancer Genome Atlas (TCGA) collaboration^5^. With millions of documented mutations, efforts have shifted towards advancing lists of specific cancer driver genes and mutations. On the line of such efforts, the OncoKB-a precision oncology knowledge database-has expanded the number of cancer genes to 1,019, highlighting actionable alterations^6^, while the CGC has catalogued 719 genes matched to cancer hallmarks and grouped into two tiers (tier 1 with documented activity in cancer, and tier 2 with strong indication of having a role in cancer). Recently, the PanCancer Atlas project reported results that were generated through an effort that encompassed the analysis of >10,000 tumors across 33 different cancer types. To analyze the data, a variety of bioinformatics algorithms and tools have been developed for quality control, reproducible computing, elimination of biases, handling of noise and false positives, and, overall, for dealing with the logistics of handling such large datasets^7^. Multi-institutional collaborations, such as the MC3 project, were launched for the very purpose of providing resources and methods, and for outlining best practices^7^. Novel software tools have been developed for performing advanced tasks for data alignment^7^, normalization, scoring, and curation. Specific needs such as variant or gene fusion calling, annotation, filtering, validation, indel location, or identification of splice creating mutations were addressed. Additional tasks focused on assessing correlations between cancer aneuploidy and mutations (or mutation rates), and the expression of proliferation and immune signaling genes^8^. Overall, the effort resulted in entire mutational landscapes and signatures that have been proposed for assessing the functional consequences of mutations in DNA repair genes^9^ and splicing factors^10,11^, and in genes with splice-site-creating^12^ and immune response^13^ role. For example, a set of mutation calling algorithms were advanced for generating a dataset of ~3.6 million somatic variants, of which ~3.4 million were single nucleotide variants (SNVs)^7^. Both the software methods and the datasets were made publically available. From the same source of PanCancer tumor exomes, Bailey *et al*. proposed a total of 299 cancer driver genes encompassing over 3,400 putative missense driver mutations, which when further refined by using three structural level tools, resulted in 579 driver mutations associated with 53 genes^14^. The missense driver mutations appeared to be more prevalent in oncogenes than in tumor suppressors, the driver genes being associated with biological processes related to RTK and immune signaling, transcription, preservation of genome integrity and protein homeostasis and ubiquitination. Many driver genes were associated with a single cancer type, and >50% of the samples contained druggable alterations. Alternatively, tumor suppressors were richer in truncations and frameshifts, and appeared to harbor rare cancer predisposing alleles^15^. Studies have also suggested that gene fusions play a role in the development of ~16% of cancer cases, and that such fusions may provide leads for targeted and immune-based therapies^16^.

Despite the abundance of data, due to the variability introduced by different sequencing technologies, depth of sequencing and data-processing pipelines, compounded by a heterogeneous biological background, the identification of driver mutations even within cancer–driver genes continues to pose a challenge. Studies focused on characterizing cancer cell lines by using reversed-phase protein arrays have shown that protein expression captured better the signals from genetic alterations than mRNA expression, and that correlations between patient cohorts and cell lines do exist (i.e., in terms of top mutated genes, mutation frequencies, and associated pathways such as p53, PI3K, NOTCH, and SWI/SNF complex signaling)^17^. Similar copy number abnormalities (CNA) were also found in cell lines and breast tumors^18^. Overall, however, peptide-level confirmation of genomic and transcriptomic variants has been proven to be low due to limited protein sequence coverage via tandem mass spectrometry (MS/MS) analysis, presence of posttranslational modifications (PTMs), and protein degradation^19,20^. Moreover, certain samples have been shown to display anti-correlations between mRNA and protein levels as a result of gene-level CNAs^19,20^. Therefore, future protein-level identification and confirmation of genomic alterations and coding mutations is expected to play an essential role in the discovery of cancer driver genes and their mutations, in assessing the impact of these alterations on the regulatory mechanisms that govern aberrant cell behavior, in the classification of cancer types and subtypes, in the identification of actionable mutations, and ultimately in the development of precision medicine therapeutic approaches. In this work we describe the results of mass spectrometry-based profiling of three cell lines that led to the identification of single amino acid mutations in over 200 proteins. We describe the data selection process, evaluate the mass spectrometric quality of the data, and assess the biological relevance of the observed peptide alterations.

## Results

### Selection of mutated peptide sequences

Two breast cancer cell lines, representative of ER + (MCF7) and HER2 + (SKBR3) breast cancers, as well as a non-tumorigenic cell line (MCF10A), were subjected to tandem mass spectrometric analysis to enable proteome-level detection of altered peptide sequences. The use of various cell states (cell cycle-arrested and proliferating cells), three biological and five technical replicates, as well as of nuclear/cytoplasmic enriched cell extracts, provided a rich resource for enabling a comprehensive examination of the cells. The proteomic profiling of the three cell lines led to the identification of a few thousand protein groups matched by ~20,000 unique peptide sequences, when a conventional database search against a reviewed, canonical *Homo sapiens* database was performed. The search for matches between the experimental data and the theoretical protein sequences in the database was performed with the Proteome Discoverer 1.4 software package that included the Sequest HT search engine. The identification of mutated peptides was accomplished with the aid of an additional database (XMAn-Unknown mutation analysis database) that comprised over 700,000 mutated entries formatted in FASTA format^21^. XMAn-v1 was built with information extracted primarily from the COSMIC database^2^, and comprised mainly missense mutations and a few small-scale insertions, deletions and frameshifts. When the MS raw file search parameters were modified to include the mutated entries, a list of an additional 275 proteins matched by 294 unique mutated peptides (402 MS/MS hits), carrying mainly missense mutations and a few deletions/frameshifts and insertions, was obtained. Only 265 of these proteins could be matched to reviewed UniProt IDs. The list of mutated peptides was selected from the database search results by applying criteria that minimized the false matches, i.e.: (a) The FDRs were set to <1% and <3% for high and medium confidence matches, respectively; (b) The peptides that included amino acid substitutions with close mass, that resulted in small m/z differences for multiply charged ions that are difficult to distinguish be ion trap instruments, were excluded (i.e., Q → E, E → Q, D → N, N → D, Q → K, K → Q, K → E, E → Q, L → I, I → L, I/L → N, where the masses of the residues are K = 128.17, Q = 128.13, E = 129.11, D = 115.09, N = 114.10, I/L = 113.16); and (c) The matches were retained only if they were unique to the XMAn-v1, but not common to both the *Homo sapiens* and XMAn-v1 databases.

### Description of parameters that characterize the mutated peptides

**Figure 1:**
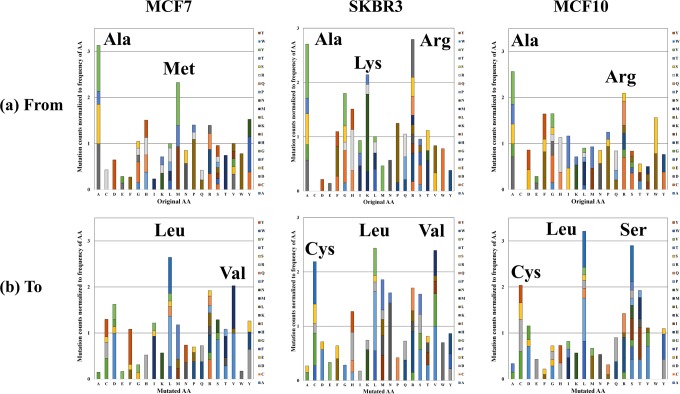
Stacked column charts depicting the relative frequency of amino acid mutations. (**a**) Charts representing the number of mutations from each amino acid in the original protein sequence to a mutated residue; (**b**) Charts representing the number of mutations to each amino acid from the original residues. The mutation counts were normalized to the frequency of the respective amino acids in the human proteome.

A breakdown of the mutated database search findings is presented in Supplemental Table S1. The results include the mutated peptide sequences, the number of spectral counts, the UniProt IDs, the gene names, the protein group accession names, the gene and protein-level mutation sites and type, a short sequence for identifying the site of the mutation in a peptide, other possible UniProt organism matches, the non-mutated sequence counterparts, the cell line presence, and a description of the protein function extracted from UniProt. For some mutated peptide entries, the ID of the parent protein was updated manually to the ID from the canonical/reviewed UniProt database. This was necessary either because the peptide was recognized in XMAn-v1 under a gene name or some other identifier, or because the mutated peptide was assigned to another master protein group during the XMAn-v1 search than its non-mutated counterpart in the canonical database search. In addition, the table includes one set of search engine results and scores, i.e., Xcorr, missed cleavage site, charge state, m/z, MH + , difference between the theoretical and experimental mass (Δm), LC retention time, and identification confidence criteria. With few exceptions, most peptide identifications were of high confidence. The Xcorr score values for these peptides indicated good quality matches between the experimental and theoretical mass spectra [34 peptides (1 + ), Xcorr = 2.34–3.56; 152 peptides (2 + ), Xcorr = 3.34–5.31; 200 peptides (3 + ), Xcorr = 3.87–6.26; and 16 peptides (4 + ), Xcorr = 3.98–4.74). Additional information, such as altered peptide domains and BLOSUM62 scores^22^, was also added to the table. Per cell line, 139 (SKBR3), 124 (MCF7), and 119 (MCF10) mutated sequences were identified, representing 126, 111 and 110 proteins, respectively. Stacked column charts illustrate the distribution of missense mutations in each cell line, from- and to- each amino acid, normalized to the frequency of the amino acids in the human proteome (Fig. 1). As shown in previous work, the normalized frequency of random mutations is expected to not show much variability among amino acids^21^. Similar trends were observed in this dataset as well, however, mutations from Arg, Ala, Lys and Met, and to Cys, Leu, Met, Asn, Arg, Ser, Thr and Val, were encountered at higher frequencies than other mutations in some of the cell lines. The higher frequency of mutations from Arg was in line with previous observations that the CpG dinucleotide sequence, which is present in four out of the six Arg codons, mutates at higher rates than other dinucleotides^23^. Mutations from Glu (E) and Asn (N) were under-represented, most likely due to the elimination from consideration of mutations that involved amino acids with close mass (see above). Overall, the largest mutation frequencies that could lead to changes in amino acid properties from basic to acidic or from polar to nonpolar, or *vice versa*, with possible impact on protein structure and function, were encountered for the following substitutions: A → D, A → S, D → Y, H → R, K → N, M → I, P → L, Q → R, R → C, R → Q, R → H, and T → M.

### Artifacts that can lead to false positive identifications

Visual inspection of the tandem MS data revealed some noisy spectra with un-assigned product ions, despite the low FDRs that were associated with these mass spectra. The database search engine enabled the identification of only *b*, *y*, *a*, and H_2_O/NH_3_ neutral loss ions. As a result, some tandem mass spectra displayed a few but rather intense unassigned ions, which often were found to belong to internal fragments or other neutral losses. Manual assignment of these ions can be completed with tools such as ProteinProspector^24,25^. Noisy spectra, or spectra in which intense fragment ions cannot be accounted for, are produced either from low abundance precursors, multiple overlapping peptide parents (including ions with posttranslational modifications), or false matches to the database. Peptide alterations can be confirmed in such cases via sequencing or targeted multiple reaction monitoring (MRM)/MS. A subset of 52 mutated sequences with high quality tandem mass spectra were included in Table 1, with spectral data being provided in the Supplemental Dataset S1. Differences between theoretical and experimental masses that exceeded 100–300 ppm (values typical of low mass accuracy ion trap instruments) were most often due to the fact that the peptide’s 2^nd^ or 3^rd^ isotopes were more intense than the 1^st^ one, and were selected consequently for fragmentation. As a result, it was the mass of the 2^nd^ or 3^rd^ isotope that was compared to the theoretical monoisotopic mass, leading to the observed differences. For 70 of the mutated peptides, non-mutated peptide counterparts were found. The relative abundance of the mutated and non-mutated peptides, as well as of their parent proteins, was added to Supplemental Table S1, with spectral counts used as a measure of abundance. Spectral counting is a semi-quantitative method that estimates the abundance of peptides and proteins based on the total number of tandem mass spectral matches. Highly abundant proteins are typically represented by many peptides and a large number of counts.

**Table 1:** Selected peptides with mutated sequences.

Sequence	UniProt ID	Protein Name	Mutation	Cells line presence	Pfam Domain
AFQHLSEAVQAAEEEAQPPSWSCGLAAGVIDAYMTLADFCDQQLR	P78527	DNA-dependent protein kinase catalytic subunit	P3405L	MCF10	FAT
**AGNVSKDEIDSAVKMLVSLK**	P23381	Tryptophan–tRNA ligase, cytoplasmic	A31V	MCF10	WHEP-TRS
AINQSQSVQESLESLLQSIGEVEQNLEGK	Q9UPN3-5	Isoform 4 of Microtubule-actin cross-linking factor 1	A2547?	MCF10	
ALMLQGVDLLDDAVAVTMGPK	C9JL25	60 kDa heat shock protein, mitochondrial (Fragment)	A90D	MCF7, MCF10, SKBR3	Cpn60 TCP1
AMQAAGQIPATALLPTMTPDGLSVTPTPVPVVGSQMTR	P26368	Splicing factor U2AF 65 kDa subunit	A131S	MCF7	
ASTVKSVLELIPELNEKGEAYNSLMK	P42704	Leu-rich PPR motif-containing protein, mitochondrial	E1318G	MCF7, MCF10	
CAPLFSGTEHHASLIDSLLHTVYRLSK	Q92736	Ryanodine receptor 2	A2536S	MCF7	
CQEKWDKLLLTSTEK	Q7L0Y3	Mitochondrial ribonuclease P protein 1	Y262C	MCF7, MCF10	tRNA m1G MT
DDLQFLADLEELITKFQVFRISQR	Q9Y6X0	SET-binding protein	H971Q	MCF7, MCF10, SKBR3	
DIMTYVSSFYHAFSGAQK	O43707	Alpha-actinin-4	A256D	MCF7, MCF10	CH
DQEGQDLLLFIDNIFR	P06576	ATP synthase subunit beta, mitochondrial	V301L	SKBR3	ATP-synt ab
**EAMQQADDWLGIPQVITPEEIVDPNVDEHSVMTYMSQFPK**	P21333	Filamin-A	L261M	MCF7, MCF10	CH
EAVMSFSITETEKIK	Q5T4T6	Synaptonemal complex protein 2-like	N353S	MCF10	
EFADSLGIPFLETSAKNAMNVEQSFMTMAAEIK	P62820	Ras-related protein Rab-1A	T159M	SKBR3, MCF7	Ras
EFDTLSGKVEESPDK	Q9BXX2	Ankyrin repeat domain-containing protein 30B	L760V	SKBR3	
EIYPYVIQERRPTLNELGISTPEELGLDKV	P20674	Cytochrome c oxidase subunit 5 A, mitochondrial	L130R	SKBR3, MCF7	COX5A
EQLQQEQALLEEIER	Q15149	Plectin	R1386Q	SKBR3, MCF7	
FGLAHLMALGLGPWMAVEIPDLIQK	Q9Y3C8	Ubiquitin-fold modifier-conjugating enzyme 1	L146M	MCF10	UFC1
FQSSAVMALQEGCEAYLVGLFEDTNLCAIHAK	P68431	Histone H3.1	A96G	SKBR3, MCF10	Histone
**FTLDCTHPVEDGIMDAANYEQFLQER**	P35268	60 S ribosomal protein L22	39Y	SKBR3, MCF10	Ribosomal L22e
GCIEKLSEDVEQLKK	P61266	Syntaxin-1B	V53L	MCF7	Syntaxin
GFDFVTFESPADAK	P38159-3	Isoform 3 of RNA-binding motif protein, X chr	A52D	MCF10	RRM 1
GFGFITFTNPEHASDAMR	P98179	Putative RNA-binding protein 3	V62D	MCF7	RRM 1
GMLDLLEVHLLDFPNIVIK	Q6P2Q9	Pre-mRNA-processing-splicing factor 8	P1871L	MCF7	PRP8 domainIV
**GTAAAAAAAAAAAAAK** (insertion in frame)	P50914	60 S ribosomal protein L14	A159_K160insAAA	MCF7, MCF10	
HQGVMVGMCQKDSYVGDEAQSK	P68133	Actin, alpha skeletal muscle	G50C	MCF7, MCF10, SKBR3	Actin
HRILPEKYPPPTELLDLQPLPVSALR	O75643	U5 small nuclear ribonucleoprotein 200 kDa helicase	L1289R	MCF7	
IMSLVDPNHCGLVTFQAFIDFMSR	O43707	Alpha-actinin-4	S823C	SKBR3, MCF7	
**KHTLSFVDVGTGK**	P31040	Ubiquinone flavoprotein subunit, mitochondrial	Y629F	MCF7	Succ DH flav C
KSQESLTENPSETLKPATSISSTSQTKGINVK	F5GXV7	Neurobeachin	I1735T	MCF10	
LCGLLVLGSWCISVMGFLLETLTILR	O60412	Olfactory receptor 7C2	S156F	MCF10	7tm 4
LCYVALYFEQEMATAASSSSLEK	Q6S8J3	POTE ankyrin domain family member E	D922Y	MCF7	Actin
LDTNSDGQLDYSEFLNLIGGLAMACHDSFLK	P31949	Protein S100-A11	F77Y	MCF10	EF-hand 1
LFDHLESPTPNPTEPLFLAQAEVYK	P49327	Fatty acid synthase	P972L	SKBR3	PS-DH
LFLASLAAAGSGTDAQVALENEVK	Q7Z6Z7	E3 ubiquitin-protein ligase HUWE1	V2153E	SKBR3	
LTENLSALQR	Q8TD16-2	Isoform 2 of Protein bicaudal D homolog 2	R398Q	MCF7	BicD
LTQAQIFDYSEIPNFPR	P00491	Purine nucleoside phosphorylase	G51S	MCF7	PNP UDP 1
MDATFIGNSTAIQELFK	P04350	Tubulin beta-4A chain	A364D	MCF7, MCF10, SKBR3	Tubulin C
MHDMNTDQENLVGTHDAPIR	O43684	Mitotic checkpoint protein BUB3	L84M	MCF7	
MLVVLRQGTREEDDVVSEDLVQQDVQDLYEAGELK	P08133	Annexin A6	L162R	SKBR3	
QVHPDTGISSKVMGIMNSFVNDIFER	P23527	Histone H2B type 1-O	A59V	SKBR3, MCF7	Histone
QVYPDTGISSKAMGIMNSFVNDIFER	O60814	Histone H2B type 1-K	H50Y	SKBR3, MCF7	Histone
SDASSGQSGSRSASRTTR	P20930	Filaggrin	R884S	SKBR3	
SLGQNPTEAELQDMINEVDADGNGTIDFPEFFTMMAR	P62158	Calmodulin	L70F	MCF7, MCF10, SKBR3	EF-hand 7
SMGIMNSFVNDIFER	Q8N257	Histone H2B type 3-B	A59S	MCF7, MCF10, SKBR3	Histone
SVIVVLRLNVDLQAVVIFELVY (del/frame shift)	Q8TC27	Disintegrin and metalloproteinase domain protein 32	K388fs*39	SKBR3, MCF7	
SYELPDGQVITIGKER	P68133	Actin, alpha skeletal muscle	N254K	MCF7, MCF10, SKBR3	Actin
**TSPDPLPVSAAPSKAGLPR**	Q13469	Nuclear factor of activated T-cells, cytoplasmic 2	S330L	MCF7	
TTGIVMDSGDGVTHTVPIYEAYALPHAILR	P63261	Actin, cytoplasmic 2	G168A	MCF7, MCF10, SKBR3	Actin
**TVSLGAGSKDELHIVEAEAMNYEGSPIK**	P06748-3	Isoform 3 of Nucleophosmin	A53S	SKBR3, MCF10	Nucleoplasmin
**VSELEEFINGPNNAHIQQVGDR**	P53675	Clathrin heavy chain 2	D1188E	MCF10	Clathrin
VTNGAFTGEISLGMIK	P60174-1	Isoform 2 of Triosephosphate isomerase	P81L	SKBR3, MCF7	TIM

### Chromosomal maps of missense mutations

**Figure 2:**
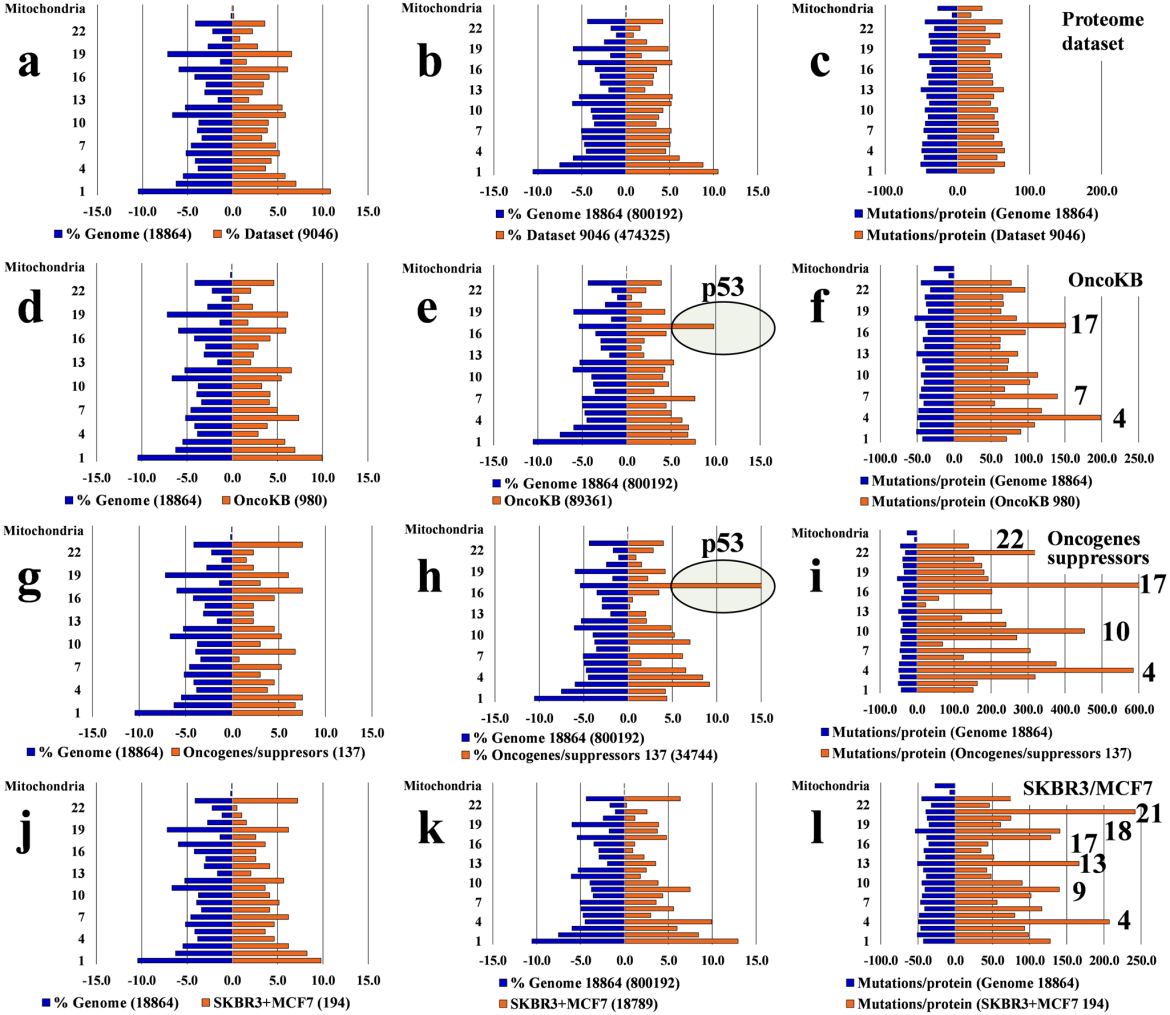
Comparison of chromosomal distributions of missense mutations in various datasets to the human genome. The left panels (blue) represent mutations in the human genome (18,864 genes), and the right panels (orange) represent mutations in various protein or gene datasets. Comparisons to the human genome are provided for: (**a**–**c**) an aggregate list of 9,046 genes coding for proteins detected in the proteome profile of MCF7, MCF10 and SKBR3 cells; (**d**–**f**) the list of 980 genes in the OncoKB database^33^; (**g**–**i**) the list of 137 oncogenes and tumor suppressors proposed in ref.^1^. (**j**–**l**) the list of 194 mutated proteins detected in SKBR3 and MCF7 cells. The 1^st^ column of histograms represents the % distribution of genes or proteins per chromosome in the human genome and in the various datasets; the 2^nd^ column represents the % distribution of missense mutations per chromosome in the human genome and in various datasets; the 3^rd^ column represents the distribution of the total number of mutations per gene or protein and per chromosome. The numbers of genes or proteins for each dataset are provided for each panel, and the 2^nd^ column provides the total mutation counts per dataset, as well. All mutation counts represent the catalogued values for the existing genes in the mutation database that was used in this study.

To assess the extent to which mass spectrometry can be used to map the chromosomal profile of missense mutations (reflected, so far, mainly by gene- and transcript-level sequencing results), we evaluated the chromosomal distribution of catalogued substitutions for the entire *Homo sapiens* database (18,864 gene entries for which the locus was found), and compared it to that of several datasets: aggregate lists of genes (**a–c**) for which proteins were detected in various experiments conducted in our laboratory with breast cancer and non-tumorigenic cells (~1,500–9,000 entries); lists of cancer genes, oncogenes and suppresssors such as proposed by OncoKB (1,019) (**d–f**) and Vogelstein *et al*. (137) (**g–i**)^1^; and list of genes for which mutated proteins were detected in the present work (**j-l**). These gene or protein sets were queried for the hypothetical presence of any substitution that was included in the XMAn-v1 database. Representative histograms built with these data are provided in Fig. 2. Each panel comprises the distribution of mutations for a particular dataset (right side of each panel) compared to the genome-level distribution used as a control (left side of the panel). The three columns represent distributions for the % genes or proteins per chromosome (column 1), % mutations per chromosome (column 2), and mutation counts per gene or protein and per chromosome (column 3). The % genes (or proteins) per chromosome was calculated based on the chromosome distribution of human genes for which the chromosome location could be extracted from the HGNC database (note that the numbers on display are lower than the ones submitted for search, due to less than 100% match in HGNC). The % mutations and mutation counts per gene and per chromosome were calculated based on the number of somatic mutations recorded in the COSMIC database (v. 66) and included in XMAn-v1.

## Discussion

The analysis of the data was geared towards assessing the validity and relevance of the observed peptide alterations. In terms of validity, factors related to possible sources of contamination, to artifacts that may lead to sequence miss assignments, and to abundance relationships between mutated and non-mutated counterparts, were addressed. In terms of biological relevance, issues that tackled subjects related to the identification of matches to cancer driver genes, to the impact of peptide alterations on biological function and protein-protein interactions (PPIs), as well as to the utility of mass spectrometry to generate chromosomal maps of missense mutations of relevance to diagnostics, were explored.

To identify possible sources of contamination, the list of mutated peptides was further scrutinized by performing a search against the entire UniProtKB database^26^. The search resulted in the identification of 32 unique sequences matched to 1,040 catalogued organisms (human, mouse, rat, orangutan, birds, insects, nematodes, bacteria, fungi, and various parasites), that included 9 matches to *Homo sapiens* (5 reviewed, 4 non-reviewed), 3 mouse reviewed, and 20 non-reviewed sequences. These sequences were flagged in the list. Contamination would have been expected to occur mainly from bovine proteins originating from the FBS added to the culture medium, but there was only one mutated peptide match to a non-reviewed protein. Consequently, contamination from bovine proteins or other organisms was deemed unlikely, as the observed matches belonged to non-reviewed sequences. Contamination of biological origin, if present, would have rather returned candidates matched to more abundant, reviewed protein sequences.

The abundance relationship between the mutated peptides and their non-mutated counterparts was further explored. The mutated peptides accrued only a few spectral counts, characteristic of low abundance, while their non-mutated counterparts accrued from a few to hundreds or thousands of counts. The total count for the parent proteins followed similar trends. For some mutated peptides, the parent protein was not identifiable in the canonical UniProt database. The spectral count values for the non-mutated peptide counterparts and the parent proteins were appended to Supplemental Table S1. The scenarios that could lead to the observed combinations of spectral counts are summarized in Table 2. The spectral count observed for a given peptide will be determined by the number of cells carrying a specific mutation in a larger population of cells, and the abundance of the protein carrying the mutation. In practice, the detectability of low abundance proteins is generally poor, and certain peptides may not be observable even if they are consistently present in the entire population of cells. Our data suggest that the observed peptide alterations were present in low abundance either because were originating from a small sub-population of cancer cells with various levels of protein abundance, or from a larger population of cells with low protein abundance. Low spectral counts for high-abundance peptides can also be explained. Poor enzymatic protein digestion, loss during sample preparation, physico-chemical properties that do not support a strong ESI signal, or the presence of PTMs, are additional factors that hamper detection even in the case of abundant peptides.

**Table 2:** Hypothetical spectral count scenarios that reflect the detectability of mutated peptides and proteins in a subpopulation of cancer cells that carry the mutation.

	Small cell number/Low abundance protein	Large cell number/Low abundance protein	Small cell number/High abundance protein	Large cell number/High abundance protein
Spectral counts of the mutated peptides	Zero-to-low	Low	Low	High
Spectral counts of the non-mutated counter-peptides	Low	Zero-to-low	High	Low
Spectral counts of the mutated protein	Low	Low	High	High

Two scenarios, low and high, of the number of cancer cells carrying a specific mutation in a larger population of cancer cells, and of the abundance of the protein carrying the mutation, are considered.

From a biological stance, transcriptomic and proteomic studies of aneuploid cells have shown that changes in mRNA and protein expression levels generally correlate with changes in the chromosome copy numbers^27,28^, *albeit* it appears that cellular mechanisms that can adjust the abundance of certain proteins to normal levels, do exist (e.g., the case of protein subunits that are part of macromolecular complexes and kinases^28^). Consistent with these findings, proteomic analysis of disome V and XIII yeast cells displayed an average of 1.8–1.9-fold increase in protein expression levels for genes located on these chromosomes, relative to the wild-type^29^. The MCF7 and SKBR3 cells have a complex chromosomal composition, having a modal chromosome number of 82 and 84, respectively. As aneuploidy drives the acquisition of various abnormal cell karyotypes and genomic instability in cancer cells, it would be difficult to draw straightforward correlations between chromosome copy numbers and protein abundances in a heterogeneous cell population. Standing genetic variations in small sample sizes would not be inferrable either. One can speculate only that the proteins that are detected by many spectral counts, and that display mutated sequences but no non-mutated counterparts, carry mutations that occurred early in the population. However, it is also possible that a wild-type sequence generates a peptide that cannot be cleaved by trypsin or detected by MS. If immunoprecipitation can generate sufficient protein for reliable analysis (e.g., 25 fmol/mg total protein extract), targeted MRM-MS could be used to accurately quantify selected mutant and non-mutant peptide pairs and verify such hypotheses^30^. Spectral counting, on the other hand, could provide insights into the impact that certain mutations have on the global regulatory mechanisms that drive protein expression. Overall, as most of the observed alterations are expected to be stochastic in nature, mutations that are detected consistently in multiple cell lines should be subjected to closer scrutiny, as these mutations may represent either already known variants, or peptides, possibly chemically modified, that were erroneously matched to the altered sequences. The presence of such mutations should be corroborated by orthogonal technologies (e.g., sequencing or MRM-MS).

The exploration of aggregate results from large sequencing projects of cancer tissues and cell lines that have been deposited in public databases^31–34^ enabled so far the advance of several panels of genes and proteins believed to play a significant role in the development of cancer. The peptide-level alterations that were identified in this work were compared to the COSMIC CGC^27^, OncoKB^29^, and CCLE^28^ databases, and to the proposed 579 driver genes^14^ and 137 oncogene/suppressor^1^ datasets. Gene names were matched to UniProt entries based on nomenclature provided by HGNC (HUGO Gene Nomenclature Committee^35^). Protein matches have been found mainly to the proteins included in the CGC and the OncoKB, and a few to the list of 137 oncogenes and suppressors (35 total)^1^. Supplemental Table S2 provides the full list of mutated proteins in each cell line, the matches to the cancer genes and their locus, as well as the total number of catalogued mutations for these genes. Specific missense mutation matches have been found to six proteins from the COSMIC Cancer Gene Census database (FLNA, RPL22, SDHA, NFATC2, NPM1, and CLTCL1). Five of these mutations carried a high pathogenicity score (0.81–0.99), with potential relevance to cancer, and three were also present in the TCGA datasets. These mutations were identified in a variety of tissues (endometrium, pancreas, skin), and one mutation in the SDHA protein (Y629F) was observable in eight different cancerous samples (adrenal gland, lung, haematopoietic and lymphoid, and soft tissues). In addition, two matches have been found to the mutations from the COSMIC Cancer Cell Line project, of which one pathogenic missense in the WARS protein, and one neutral insertion in RPL14. These eight mutated peptides were highlighted in bold in Table 1, and a description of the mutations was provided in Supplemental Table S3, including their COSMIC and TCGA IDs. The identification of only a few specific mutation matches, out of 52, was not unexpected. Previous reports have also noted that peptide-level confirmation of genomic and transcriptomic variants via MS is generally low^19^. Multiple reasons can be invoked to explain this outcome, including low protein sequence coverage by tandem MS, the presence of PTMs that hamper MS detection, protein degradation, poor correlation between mRNA and protein levels, and the very absence of these mutations from the XMAn-v1 database, to start with. For example, only ~100 MCF7 and 5 SKBR3 unique amino acid mutations, from the total of 670 and 275 mutations, respectively, that were included in the CCLE database, were also present in XMAn-v1. It must be also emphasized that cancer cells represent a heterogeneous population with an unstable genome that evolve new mutations at much higher rates than normal cells (~10–10,000X)^36^, with 2–8 driver mutations per cell being predicted to be necessary for tumor formation^1,37^. Therefore, correlations between genomic, transcriptomic and proteomic results that describe somatic mutations would be best pursued from data generated from the same cell population, not from populations that have diverged during a large number of doublings.

**Figure 3:**
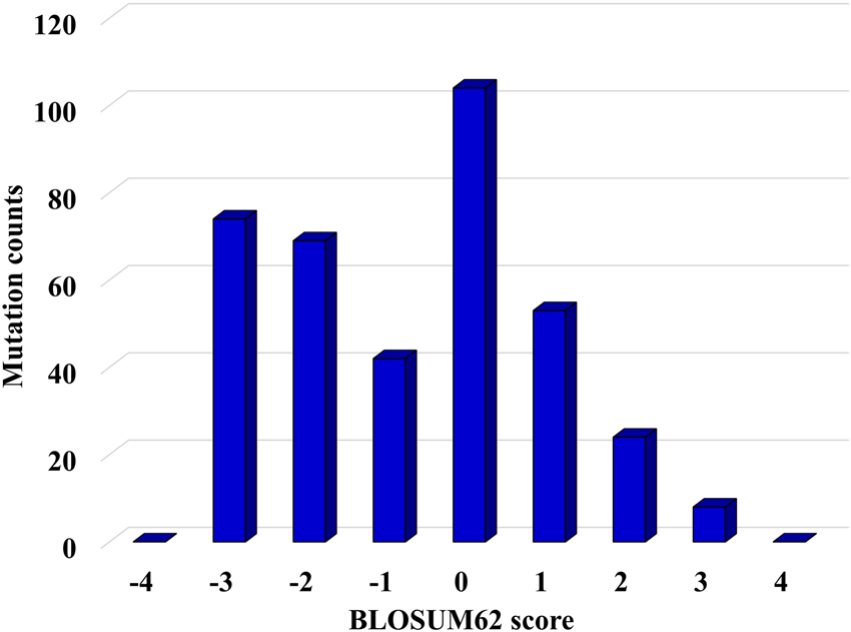
Column chart representing BLOSUM62 scores associated with the observed amino acid substitutions. The scores were assigned based on the BLOSUM62 substitution matrix and reflect the frequency of amino acid substitutions in conserved regions of protein families. More negative scores reflect less likely substitutions, while more positive scores reflect more likely substitutions. For scores close to zero, the observed frequency of a substitution approaches the expected value.

A variety of scoring systems have been developed to assess the probability of a substitution. The BLOSUM (BLOcks SUbstitution Matrix) scores reflect the frequency of amino acid substitutions in conserved regions of protein families^22^. The scores are log-odd ratios of observed per expected-by-chance substitution frequencies obtained from local alignments of evolutionarily divergent protein sequences. The advantage of using BLOSUM scores is that the likelihood of a substitution can be assessed based on the % similarity of the protein alignments. For example, the commonly used BLOSUM62 matrix, was built using protein sequences with <62% similarity. The scores range from (−4) to (+11). For a score of zero, the observed frequency of substitution equals the expected one. Scores that are less or larger than zero are reflective of lower or higher observation frequencies, respectively. Mutations that lead to significant differences in the physico-chemical properties of an amino acid upon substitution are less conservative and will have lower BLOSUM scores (i.e., are less likely to be preserved in conserved protein regions where the functional impact of the substitution can be high). Espinosa *et al*. have proposed that cancer driver mutations, in comparison to neutral ones, will be less conservative, have higher functional impact, and will be characterized by lower BLOSUM scores^38^. Calculation of BLOSUM62 scores for the observed mutations in this work led to the column chart from Fig. 3, with many amino acid substitutions clustering around zero, but also with a group of negative scores, (−2)-(−3), reflective of non-conservative mutations with potentially profound impact on protein structure and function.

**Figure 4:**
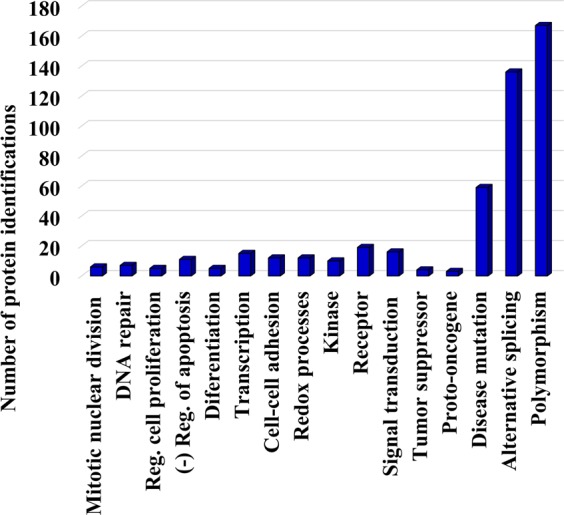
Column chart representing a few GO biological processes of relevance to cancer. Assignments of proteins to a particular category were made with DAVID tools. The selection of biological processes was made based on relevance and number of protein components per category (>4).

To uncover whether the mutated sequences have any impact on biological function, GO annotations were retrieved with DAVID (Database for Annotation, Visualization and Integrated Discovery)^39,40^ bioinformatics tools. The list of proteins included preponderantly structural components of the cytoskeleton, and proteins associated with the cytoplasm, nucleosome, plasma membrane, ribonucleoprotein complex, mitochondrion and the extra cellular matrix and exosomes. Aggregate data representing a few biological processes of potential relevance to cancer, matched by more than 4–5 proteins, were captured in Fig. 4. As it would be expected for the outcome of random mutation events, significant enrichment in specific processes could not be observed, the results being rather reflective of categories supported by the more abundant proteins that are easier to detected. It must be also noted that if the variants were SNPs, their likely role in cancer development may no longer be relevant. Out of the 52 selected peptides, six presented mutations that could be matched to one or more databases that compile natural variants, SNPs and clinically relevant mutations^41–46^. The availability of such protein-level alterations may prove to be useful, however, to predicting an individual’s susceptibility to disease or therapeutic response.

To assess whether the observed peptide alterations can affect protein function, the domains associated with the mutated sequences were extracted from the Pfam^47^, PROSITE^48^, and InterPro^49^ databases. A total of 139 unique domains were identified for the 294 mutated peptides sequences. The domains and their associated functions were included in Table 1 and Supplemental Tables S1 and S3. These domains included: chaperone/heat shock domains (Cpn60 TCP1), structure stability and filament formation (CH, Tubulin C, Actin), DNA/RNA synthesis, folding and processing (Histone, WHEP-TRS, RRM 1, PRP8 domainIV), and catalytic functions associated with metabolic pathways, redox, and transport (ATP-synt ab, COX5A, Succ DH flav C, PS-DH, TIM, BicD). Other domains were associated with DNA repair (Ku C), cytokinesis (Dynamin N), apoptosis and cell migration (Death, Peptidase C2), and zinc fingers or zinc dependent catalytic activities (zf-C2H2, ADH zinc N, carb anhydrase). Several domains were linked to functions related to peptide cleaving (Reprolysin), protein degradation (Rad4), and ubiquitination (UQ_con, UFC1). Protein kinase domains (Pkinase_Tyr) with involvement in proliferation, differentiation, and apoptosis, were also identified. A recurring domain across these mutated sequences included the different EF-hand motifs. Upon calcium binding, the conformational change of the EF-hand domain is responsible for the activation or inactivation of the specific protein. Experimental analysis and computational predictions can be used to assess whether the implicated alterations have any impact on protein function, as evidenced by protein folding, stability, cellular localization, gain/loss or switch of function, ability to form PPIs, and druggability^50^. To begin with, it has been shown, for example, that α-helices are more tolerant toward single amino acid substitutions than β-sheets^51^. In the absence of physico-chemical and structural data, evolutionary analysis can provide insights into the aggregate impact of a mutation not just on cellular function, but also on organ physiology^50^.

A few substitutions of potential relevance are discussed in the followings. The DNA-dependent protein kinase catalytic subunit (PRKDC/P78527) carried a mutation (p.P3405 → L) in its FAT domain, which has Tyr kinase activity. PRKDC is involved in nonhomologous end-joining, a repair mechanism that targets double strand breaks mainly in the G1 stage of the cell cycle^52^. The Ras-related protein RAB1A carried a mutation at T159 → M. The protein has GTPase activity and has multiple roles in cell adhesion/migration, protein trafficking, and cellular defense against pathogenic bacteria (P62820)^53^. It has been suggested that RAB1A is an mTORC1 activator and an oncogene^54^. Tryptophanyl-tRNA synthetase (WARS) has been shown to regulate the ERK, Akt and eNOS pathways (UniProt P23381), and it is believed to be involved as a negative regulator of cell proliferation^55–57^. Succinate dehydrogenase A (SDHA) represents the flavoprotein catalytic subunit of the succinate dehydrogenase complex II of the mitochondrial electron transport chain (P31040). The substitution of Y629 with F in SDHA was catalogued as variant rs6960. It has not been associated yet with disease (dbSNP and Ensemble). Nevertheless, it was catalogued as pathogenic in the CGC database (pathogenicity score of 0.81), and has been suggested that SDHA can act as a tumor suppressor gene^58,59^. The nuclear factor of activated T-cells cytoplasmic 2 protein (Q13469) is a transcription factor that plays a role in activating the expression of cytokine genes in T-cells^60^. The protein also supports invasiveness^61^. Phosphorylation at the mutation site (S330 → L) has been documented, but polymorphism was not, and Ser 330 is not part of a functional domain. The protein subcellular localization, however, is controlled by phosphorylation at multiple sites in the 99–363 region, and has a regulatory role in gene transcription. The RRM1 (RNA-recognition motif 1) domain, which was mutated in P38159 (A52 → D), has been found to be essential for the oncogenic activity of the proto-oncogene SRSF1 splicing factor that is required for the activation of the B-Raf-MEK-ERK pathways and cellular transformation^62^. Altogether, while based solely on these data direct oncogenic roles cannot be associated with the observed amino acid substitutions, their protein-level identification can support a more refined interpretation of the mutational landscapes that are considered when assessing pathogenicity. An interruption of their PPIs, as a result of a change in structure, would be one of the first effects with direct impact on function.

Genome instability leads to alterations that affect randomly gene integrity. The heterogeneous genome profile of cancer cell populations is defined by an ensemble of mutations that lead to gain or loss of function in oncogenes and tumor suppressors, respectively^63^. Most often, such mutations overlap an aneuploid state that can further trigger changes in chromosome numbers, and losses or gains in critical chromosome regions that harbor such genes. Alterations in the structure of oncogenes and tumor suppressors lead to abnormal protein expression, loss of protein function, perturbed protein-protein interactions, and ultimately to an abnormal cellular response that supports uncontrolled cell proliferation and behavior. To gain a global view over our ability to map genome-scale mutations at the protein level, in Fig. 2 we compared the chromosomal profile of the catalogued missense substitutions in various datasets with that of the entire *Homo sapiens* database. The first row of panels in Fig. 2a–c compares an aggregate list of 9,046 proteins that were identified in the MCF7, SKBR3 and MCF10 cell lines, to the entire human genome. These panels also served as a control, to confirm that an all-inclusive list of experimentally detectable proteins can mirror the chromosomal distribution of the entire human genome (and, accordingly, of the associated hypothetical mutations), with no bias induced by the protein-level detection process. Histograms generated from smaller datasets (1,550–4,000 proteins), representing one cell line proteome as a whole or just nuclear or cytoplasmic cellular sub-fractions, led to similar results (data not shown). The 2^nd^ and 3^rd^ rows in the figure display the mutation distribution results for the previously reported OncoKB (Fig. 2d–f)^33^ and the list of 137 oncogenes and suppressors (Fig. 2g–i)^1^, while the 4^th^ row provides actual protein-level MS mutation data for the combined MCF7/SKBR3 cell lines (Fig. 2j–l).

We hypothesized that a non-inclusive list of genes or proteins encompassing only components that share a particular attribute (e.g., oncogenes) would not mirror the whole genome, but rather display a pattern-like behavior. As expected, for the gene-per-chromosome distributions (1^st^ column, Fig. 2d,g, j), the symmetry between each dataset and the whole genome was generally preserved, with somewhat larger discrepancies for the smaller datasets (Fig. 2g,j). The symmetry started to break in the histograms that displayed the % mutations per chromosome (2^nd^ column, Fig. 2e,h; note chromosome 17 that carries p53 and many other cancer molecular markers^64^ with a large number of mutations), and the differences became most pronounced for the histograms that compared the number of mutations per gene and per chromosome to the whole genome (3^rd^ column, Fig. 2f,i; note chromosomes 4, 7, 17 for the OncoKB, and 4, 10, 17, 22 for the list of 137). These chromosomes harbor many known or potential tumor suppressors and oncogenes, for which a large number of mutations have been catalogued (e.g., chromosome **4**: KIT, FAT1, FAT4, TET2; chromosome **7**: RELN, EGFR, KMT2C, PCLO; chromosome **10**: PTEN, MKI67, RET; chromosome **17**: TP53, NF1; chromosome **22**: NF2, EP300). The chromosomes carrying many mutations became most strongly defined in the refined list of driver oncogenes and suppressors (Fig. 2i)^1^. The mutated profiles of the SKBR3 and MCF7 cell lines that were processed in this work, as determined by mass spectrometry, were each represented by only a limited dataset of 110–125 proteins with ~8,000–11,000 catalogued gene or transcript-level mutations (Fig. 2l). These profiles displayed a rather random pattern of peaks governed by a protein selection process that was based on the sole detection of one or just a few mutated peptides per protein. A total of 26 cancer genes, combined for the two cell lines, and distributed over all chromosomes, were part of the dataset that matched this profile. Chromosomes **4** (MCF7 and SKBR3) and **17** (SKBR3) emerged as potential mimics of the upper cancer gene histograms from Fig. 2f,i. The MCF10 data did not match the cancer gene profiles, however, oncogenes with mutations were identified in these cells, as well (Supplemental Fig. 1). While it is clear that whole proteome profiling can provide a comprehensive coverage of protein identifications across all chromosomes (see panels 2a–c), and that the data confirms our hypothesis that the cancer genes could generate chromosomal mutational profiles, the small datasets of experimentally detected peptide mutations did not allow for formulating quantitative inferences at this stage. Nevertheless, similar to gene-level alterations, it is expected that the impending accumulation of MS data will support the development of extensive protein mutation databases that will define a landscape that can corroborate or complement the gene profiles and support the advance of novel driver gene and protein mutation patterns.

Altogether, while incomplete sequence coverage hampers at present the confirmation efforts and calls for conservative data interpretation approaches, the future use of combined genome- and proteome-level mutation data, complemented with protein abundance measurements, will help establish correlations between certain mutations and protein expression profiles, assess the cumulative effect of mutational hotspots, and identify genome-scale alterations that trigger abnormal cell behavior. The availability of multi-scale data will also facilitate the identification of chromosomal mutation patterns (e.g., see Fig. 2f,i) that could reveal susceptibility to cancer development, and help expand the range of predictable drug targets, recognize propensity for evolving drug resistance, and guide the choice of effective therapeutic decisions.

## Methods

### Materials

The breast cancer (MCF7, SKBR3) and nontumorigenic (MCF10) cell lines were purchased from ATCC (Manassas, VA). Cell culture reagents and media (EMEM, PBS, trypsin 0.25%/EDTA) were from ATCC, DMEM/F12 1:1 phenol-red free and horse serum from Invitrogen (Carlsbad, CA), and McCoy from Life Technologies (Carlsbad, CA). FBS was purchased either from Gemini Bio-Products (West Sacramento, CA) or from ATCC. Charcoal/dextran treated FBS was from Hyclone (Logan, UT). Cell culture media additives (cholera toxin, hydrocortisone, 17-β estradiol, L-glutamine, bovine insulin), various other reagents (NH_4_HCO_3,_ NaF, Na_3_VO_4_, protease inhibitor cocktail, DTT, urea, and acetic and trifluoroacetic acids), as well as the nuclear/cytoplasmic separation Cell Lytic™ NuCLEAR™ kit were from Sigma (St. Louis, MO). Sequencing grade trypsin for enzymatic digestion of cell extracts was from Promega (Madison, WI). C18 and SCX sample cleanup cartridges (SPEC-PTC18 and SPEC-PTSCX) were acquired from Agilent Technologies (Palo Alto, CA). DI water was from an in-house MilliQ Ultrapure water system (Millipore, Bedford, MA), and all solvents were HPLC grade from Fisher Scientific (Fair Lawn, NJ).

### Cell culture

Detailed conditions for cell culture and processing were described in previous work^65^. Three biological replicates were produced for each cell state. Briefly, the cells were cultured per manufacturer’s protocol in their optimal growth medium supplemented with various factors: SKBR3 in McCoy 5 A + FBS 10%; MCF7 in EMEM + bovine insulin 10 μg/mL + FBS 10%; and MCF10 in DMEM/F12 + horse serum 5% + EGF 20 ng/mL + hydrocortisone 0.5 μg/mL + cholera toxin 0.1 μg/mL + insulin 10 μg/mL. The culture incubator was maintained at 37 °C with 5% CO_2_. The cells were arrested for 48 h in serum free medium, and then released for 24–36 h with medium supplemented with serum and growth factors. After harvesting, the cells were frozen at −80 °C.

### Cell extract preparation

The nuclear and cytoplasmic fractions of the cells were separated with the Cell Lytic™ NuCLEAR™ kit, in the presence of phosphatase/protease inhibitors and DTT, by following the manufacturer’s recommendations. The cell extracts were aliquotted in 500 µg batches based on concentration measurements performed with the Bradford assay and a SmartSpec Plus spectrophotometer (Bio-Rad, Hercules, CA). The protein extracts were reduced with DTT 4.5 mM in the presence of urea 8 M, at 55–60 °C for 1 h. After dilution 10-fold with NH_4_HCO_3_ 50 mM, the extracts were subjected to enzymatic digestion overnight, at 37 °C, with trypsin (~30–50:1 substrate:enzyme ratio). Sample cleanup was performed with SPEC-PTC18 and SPEC-SCX cartridges, and the samples were dissolved in acidified (0.01% TFA) aqueous solution (2–5% CH_3_CN) at 2 μg/μL.

### Liquid chromatography (LC)-MS/MS analysis

A micro-LC 1100 system (Agilent Technologies) was used for performing nano-LC separations with columns prepared in-house (100 μm i.d. × 360 o.d. × 12 cm long, packed with Zorbax SB-C18 particles/5 μm), and operated at ~180 nL/min. The experimental set-up was described in detail in previous work^66,67^. The composition of the mobile phases A and B was 95:5:0.01 and 20:80:0.01 v/v, H_2_O:CH_3_CN:TFA, respectively. The eluent gradient was 200 min long, from 10% to 100% B^65^. A linear trap quadrupole (LTQ) MS system (Thermo Fisher, San Jose, CA), with electrospray ionization (ESI) operated at 2 kV, was used for performing mass analysis. ESI was produced with the aid of a fused silica capillary (20 μm i.d. × 90 μm o.d. × 10 mm long) that was inserted in the outlet of the nano-LC column. Data acquisition occurred by using a triple play data-dependent analysis method. Zoom/MS^2^ scans were retrieved for the five most intense peaks of a prior MS scan (500–2,000 m/z), with the following settings: ±5 m/z zoom scan width, 60 s exclusion duration, ±1.5 m/z exclusion width, exclusion list 200, dynamic exclusion at repeat count 1, repeat duration 30 s; collision induced dissociation at 35% normalized collision energy, Q 0.25, 30 ms activation time, 3 m/z isolation width, and MS2 trigger threshold 100 counts. Five LC-MS/MS replicates were performed for each sample.

### Bioinformatics analysis

The raw MS data were processed by Proteome Discoverer 1.4 (Thermo Fisher) using Sequest HT as a search engine, and a *Homo sapiens* non-redundant, canonical, reviewed database with 20,198 entries (January 2015 download). The search allowed for matches only to fully tryptic peptides with maximum two missed cleavages, in a mass range of 500–5,000 Da, with a minimum and maximum peptide length of 6 and 144 amino acids, and with tolerances of 2 Da and 1 Da for precursor and fragment ions (b/y/a), respectively. The peptide FDRs (calculated with the Target Decoy PSM Validator node) were set to <3% and <1% relaxed and stringent matches, respectively. No posttranslational modifications were included. To enable the identification of mutated peptide sequences, the database search process was devised such that the experimental tandem mass spectra that could not be matched to the canonical *Homo sapiens* database, were searched against a database containing only altered peptide sequences (XMAn-v1)^21^. The database comprised over 700,000 mutated peptide entries, and was developed in previous work based on information extracted from public resources (COSMIC, IARC P53, OMIM and UniProtKB), mostly from COSMIC (v. 66). For the purpose of this work, data from all LC-MS/MS experiments performed for a cell line were combined into one aggregate list. Protein annotations and functional classification of proteins were performed with DAVID 6.8^39,40^.

## Supplementary information

Supplemental Figure S1

Supplemental Dataset S1

Supplemental Table S1

Supplemental Table S2

Supplemental Table S3

## Data Availability

Data analyzed in this study are included in the Supplementary Information files that include: full list of peptides with altered amino acid sequences, peptide characteristics and Proteome Discoverer search parameters; spectral count data for the mutated peptides, non-mutated counterparts and parent proteins; matches to cancer driver genes and protein variants; full tandem mass spectra of selected peptides. The mass spectrometry raw files that generated high quality mutated peptide tandem mass spectra have been deposited to the ProteomeXchange Consortium via the PRIDE^68^ partner repository with the dataset identifier PXD014458.
