# Supplementary material for: Proteogenomic Analysis of Protein Sequence Alterations in Breast Cancer Cells
Source: Sci Rep. 2019 Jul 17;9:10381. doi: 10.1038/s41598-019-46897-z (PMC6637242; doi:10.1038/s41598-019-46897-z)
Supplement: Supplementary file 1 — Supplemental Figure S1 [file 41598_2019_46897_MOESM1_ESM.pdf]

Proteogenomic Analysis of Protein Sequence Alterations in Breast Cancer Cells

Supplemental Figure S1

[1,2]Iulia M. Lazar,* [1]Arba Karcini, [1]Shreya Ahuja, and [3]Carli Estrada-Palma

[1]Department of Biological Sciences, [2]Carilion School of Medicine, and [3]Department of Biochemistry, Virginia Tech
1981 Kraft Drive, Blacksburg, VA 24061

*Correspondence to: Iulia M. Lazar
E-mail: malazar@vt.edu
Phone: 540-231-5077

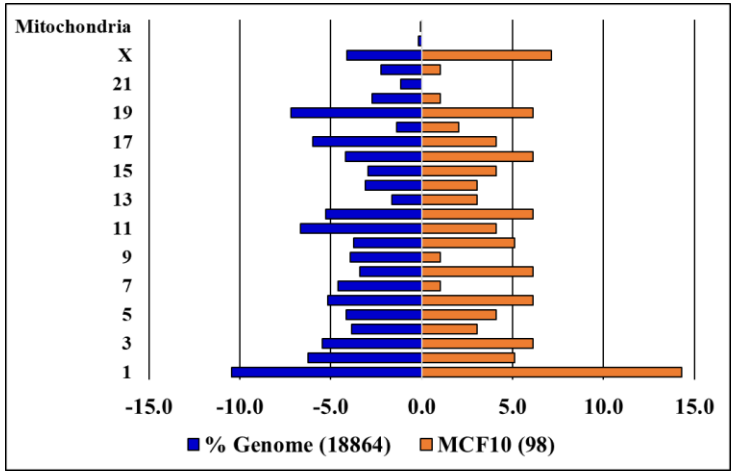
Mitochondria
X
21
19
17
15
13
11
9
7
5
3
1
-15.0 -10.0 -5.0 0.0 5.0 10.0 15.0
% Genome (18864)
MCF10 (98)

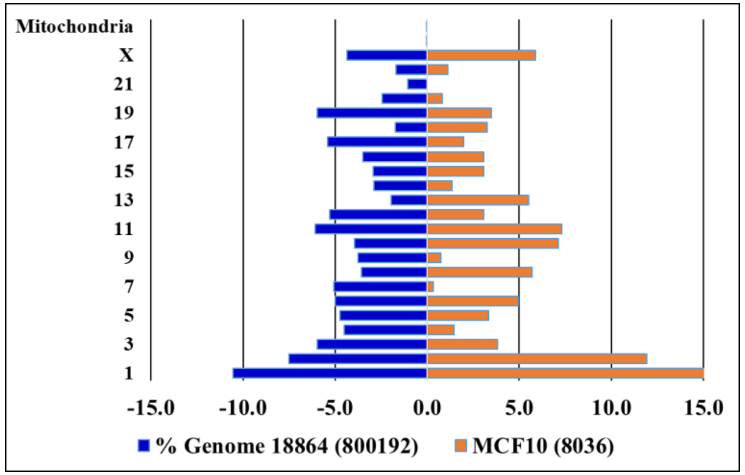
Mitochondria
X
21
19
17
15
13
11
9
7
5
3
1
-15.0 -10.0 -5.0 0.0 5.0 10.0 15.0
% Genome 18864 (800192)
MCF10 (8036)

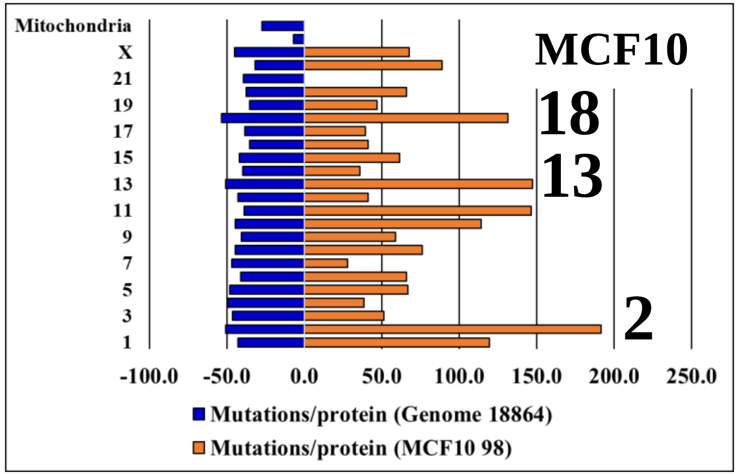
MCF10
18
13
2
Mitochondria
X
21
19
17
15
13
11
9
7
5
3
1
-100.0 -50.0 0.0 50.0 100.0 150.0 200.0 250.0
Mutations/protein (Genome 18864)
Mutations/protein (MCF10 98)
