# Supplementary material for: Proteogenomic Analysis of Protein Sequence Alterations in Breast Cancer Cells
Source: Sci Rep. 2019 Jul 17;9:10381. doi: 10.1038/s41598-019-46897-z (PMC6637242; doi:10.1038/s41598-019-46897-z)

Proteogenomic Analysis of Protein Sequence Alterations in Breast Cancer Cells

Supplemental Dataset S1

[1,2]Iulia M. Lazar,* [1]Arba Karcini, [1]Shreya Ahuja, and [3]Carli Estrada-Palma

[1]Department of Biological Sciences, [2]Carilion School of Medicine, and [3]Department of Biochemistry, Virginia Tech
1981 Kraft Drive, Blacksburg, VA 24061

*Correspondence to: Iulia M. Lazar
E-mail: malazar@vt.edu
Phone: 540-231-5077

Sequence: AFQHLSEAVQAAEEEAQPPSWSCGLAAGVIDAYMTLADFCDQQLR, Charge: +3, Monoisotopic m/z: 1624.28000 Da (+1194.34 mmu/+735.31 ppm), MH+: 4870.82545 Da, RT: 204.08 min

Identified with: Sequest HT (v1.3); XCorr:5.64

Fragment match tolerance used for search: 1 Da

Fragments used for search: a; a-H_2O; a-NH_3; b; b-H_2O; b-NH_3; y; y-H_2O; y-NH_3

Protein references (1): c.10214C>T p.P3405L|SCGLAAG|Missense|COSMIC|Autonomic ganglia(1)

CANCER_sp_P78527,PRKDC_HUMAN DNA-dependent protein kinase catalytic subunit

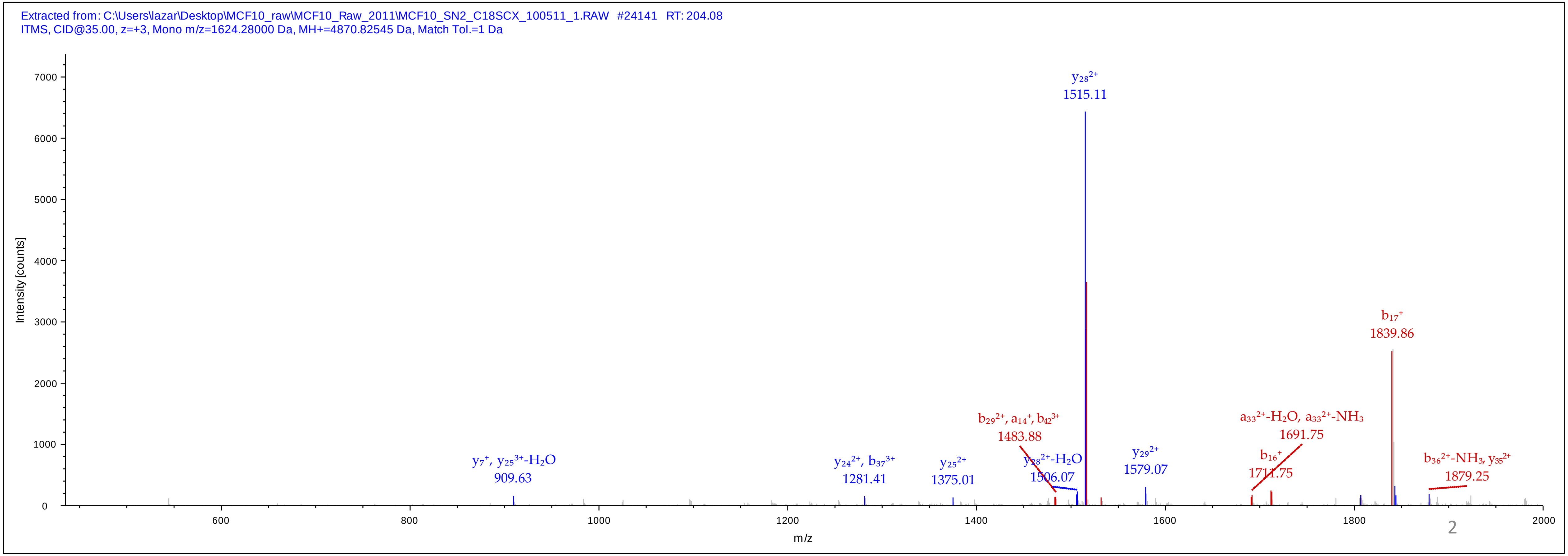

Sequence: AGNVSKDEIDSAVKMLVSLK, Charge: +3, Monoisotopic m/z: 702.26000 Da (+211.37 mmu/+300.98 ppm), MH+: 2104.76545 Da, RT: 170.12 min

Identified with: Sequest HT (v1.3); XCorr:4.20

Fragment match tolerance used for search: 1 Da

Fragments used for search: a; a-H_2O; a-NH_3; b; b-H_2O; b-NH_3; y; y-H_2O; y-NH_3

Protein references (1): c.92C>T p.A31V|AGNVSKD|Missense|COSMIC|Large intestine(1) CANCER_sp_P23381,SYWC_HUMAN Tryptophan--tRNA ligase, cytoplasmic

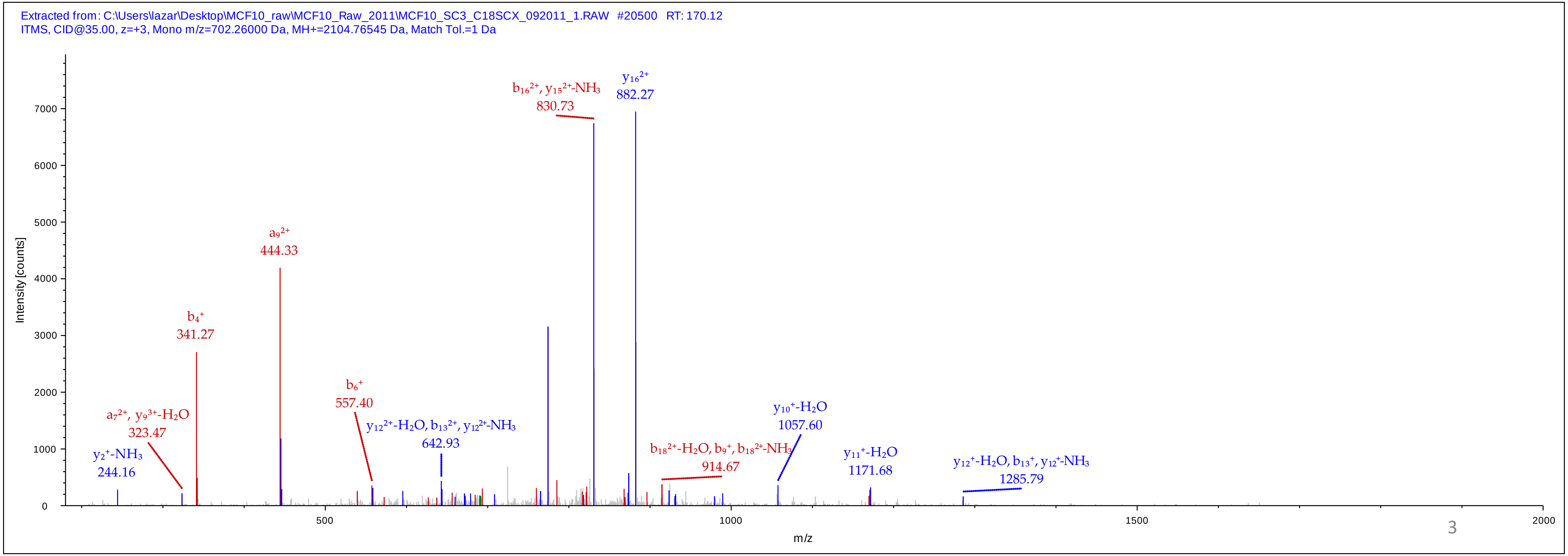

Sequence: AINQSQSVQESLESLLQSIGEVEQNLEGK, Charge: +3, Monoisotopic m/z: 1054.04000 Da (+840.34 mmu/+797.25 ppm), MH+: 3160.10545 Da, RT: 205.58 min

Identified with: Sequest HT (v1.3); XCorr:6.26

Fragment match tolerance used for search: 1 Da

Fragments used for search: a; a-H_2O; a-NH_3; b; b-H_2O; b-NH_3; y; y-H_2O; y-NH_3

Protein references (1): p.A2547>?|KAINQSQ|Missense|COSMIC|Lung(1) OR sp|Q9UPN3-2,MACF1_HUMAN Isoform 2 of Microtubule-actin cross-linking factor 1, isoforms 1/2/3/5|c.6133_6134GC>AA|p.A2045>?|KAINQSQ|Missense|COSMIC|Lung(1) - [c.7639_7640GC>AA] CANCER_sp_Q9UPN3-5,MACF1_HUMAN Isoform 4 of Microtubule-actin cross-linking factor 1, isoforms 1/2/3/5

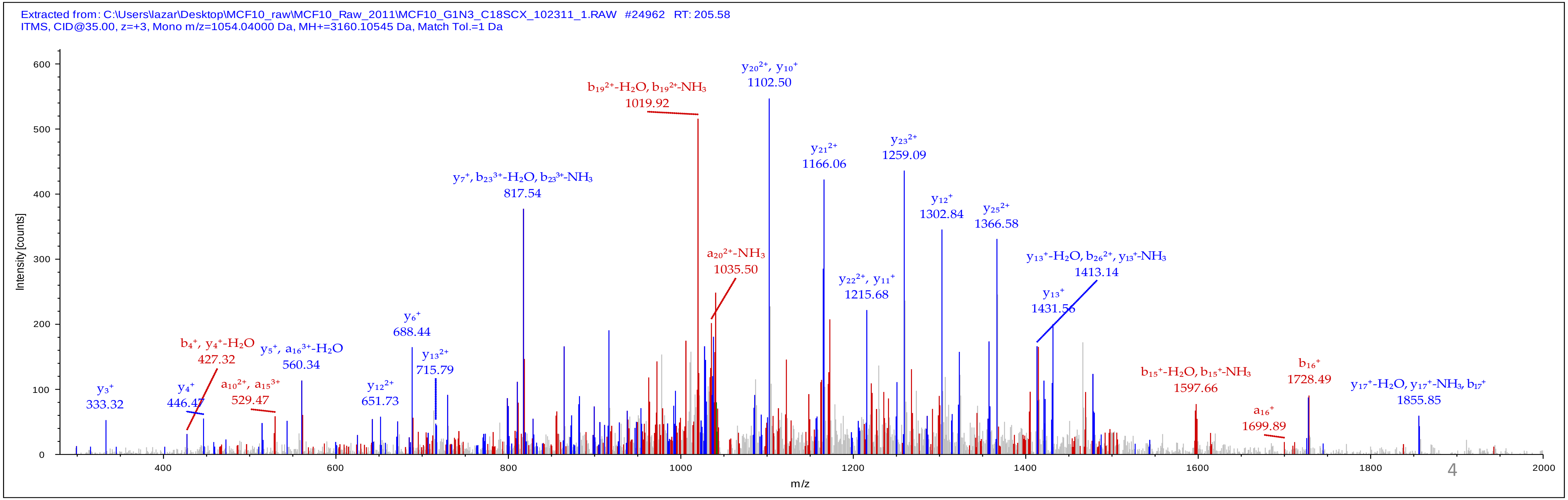

Sequence: ALMLQGVDLLDDAVAVTMGPK, Charge: +2, Monoisotopic m/z: 1080.84000 Da (+1772.42 mmu/+1639.86 ppm), MH+: 2160.67272 Da, RT: 217.04 min

Identified with: Sequest HT (v1.3); XCorr:4.11

Fragment match tolerance used for search: 1 Da

Fragments used for search: a; a-H_2O; a-NH_3; b; b-H_2O; b-NH_3; y; y-H_2O; y-NH_3

Protein references (1): c.269C>A p.A90D|DLLDDAV|Missense|COSMIC|Lung(1) OR sp|P10809,CH60_HUMAN 60 kDa heat shock protein, mitochondrial|c.143C>A|p.A48D|DLLDDAV|Missense|COSMIC|Lung(1) CANCER_tr_C9JL25,C9JL25_HUMAN 60 kDa heat shock protein, mitochondrial (Fragment)

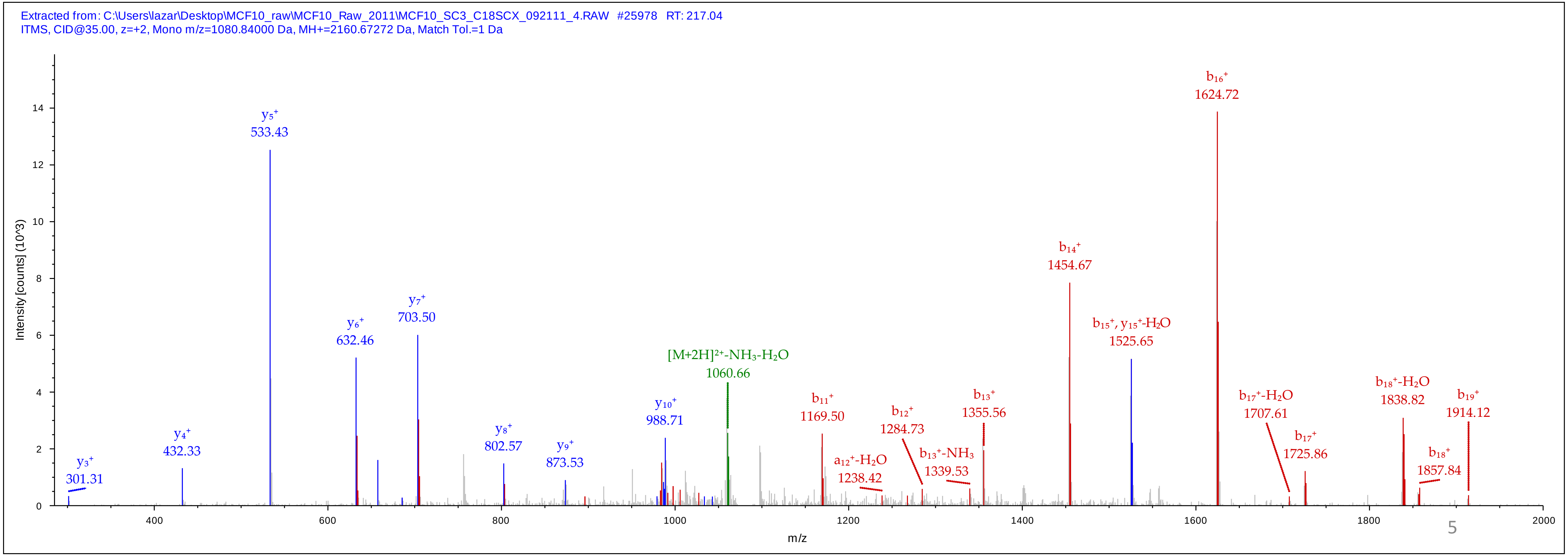

Sequence: AMQAAGQIPATALLPTMTPDGLSVTPTPVPVVGSQMTR, Charge: +3, Monoisotopic m/z: 1269.46000 Da (+465.43 mmu/+366.64 ppm), MH+: 3806.36545 Da, RT: 112.02 min

Identified with: Sequest HT (v1.3); XCorr:4.92

Fragment match tolerance used for search: 1 Da

Fragments used for search: a; a-H_2O; a-NH_3; b; b-H_2O; b-NH_3; y; y-H_2O; y-NH_3

Protein references (1): c.391G>T p.A131S|DGLSVTP|Missense|COSMIC|Lung(1) CANCER_sp_P26368,U2AF2_HUMAN Splicing factor U2AF 65 kDa subunit

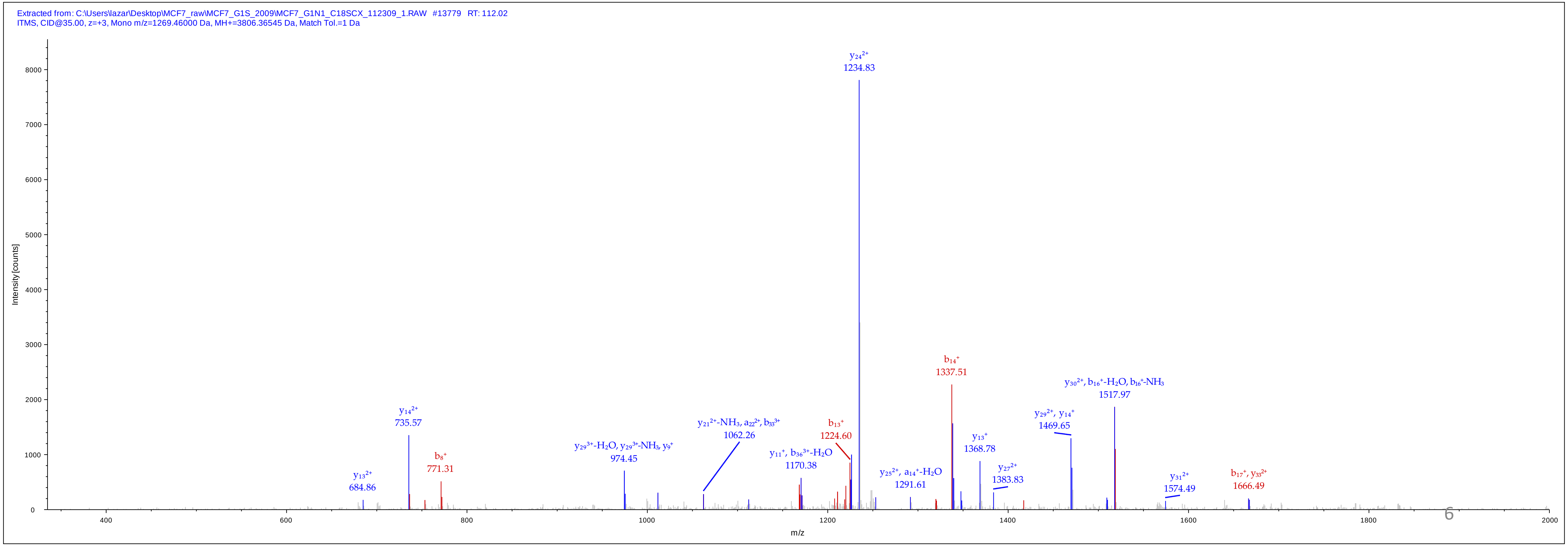

Sequence: ASTVKSVLELIPELNEKGEAYNSLMK, Charge: +3, Monoisotopic m/z: 954.88000 Da (-295.82 mmu/-309.79 ppm), MH+: 2862.62545 Da, RT: 175.52 min

Identified with: Sequest HT (v1.3); XCorr:4.38

Fragment match tolerance used for search: 1 Da

Fragments used for search: a; a-H_2O; a-NH_3; b; b-H_2O; b-NH_3; y; y-H_2O; y-NH_3

Protein references (1): c.3953A>G p.E1318G|NEKGEAY|Missense|COSMIC|Liver(2) CANCER_sp_P42704,LPPRC_HUMAN Leucine-rich PPR motif-containing protein, mitochondrial

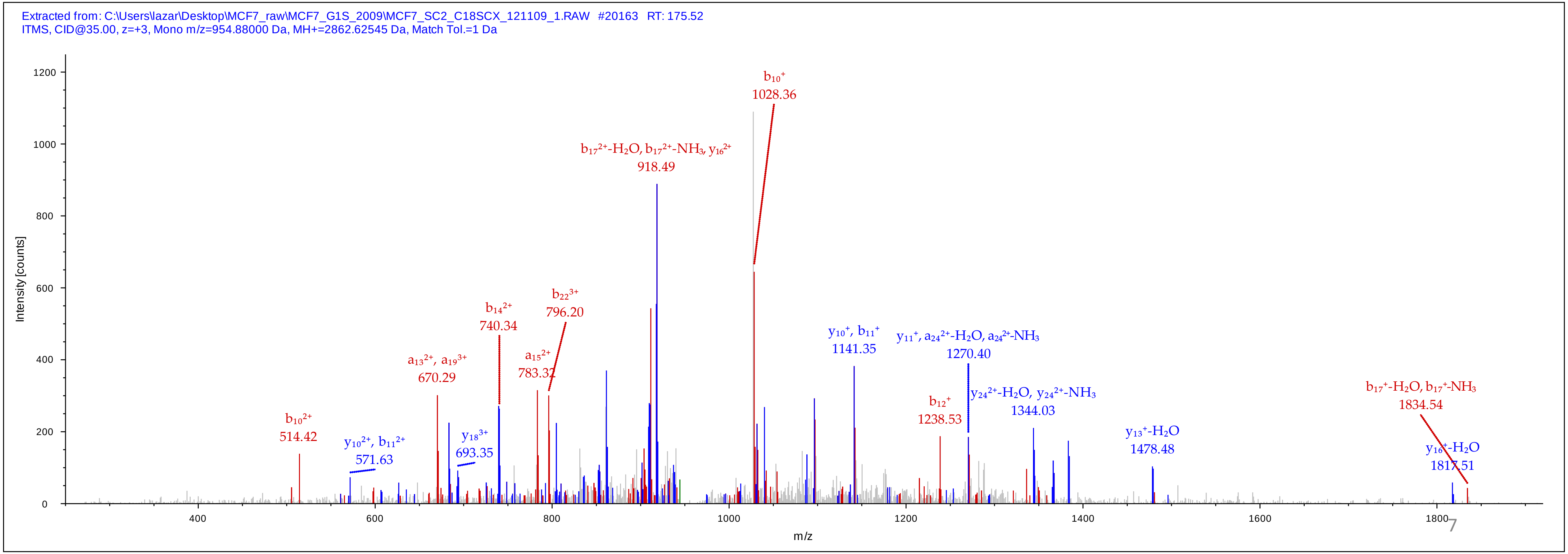

Sequence: CAPLFSGTEHHASLIDSLLHTVYRLSK, Charge: +3, Monoisotopic m/z: 999.54000 Da (+353.41 mmu/+353.57 ppm), MH+: 2996.60545 Da, RT: 106.52 min

Identified with: Sequest HT (v1.3); XCorr:4.41

Fragment match tolerance used for search: 1 Da

Fragments used for search: a; a-H_2O; a-NH_3; b; b-H_2O; b-NH_3; y; y-H_2O; y-NH_3

Protein references (1): c.7606G>T p.A2536S|PLFSGTE|Missense|COSMIC|Lung(1) CANCER_sp_Q92736,RYR2_HUMAN Ryanodine receptor 2

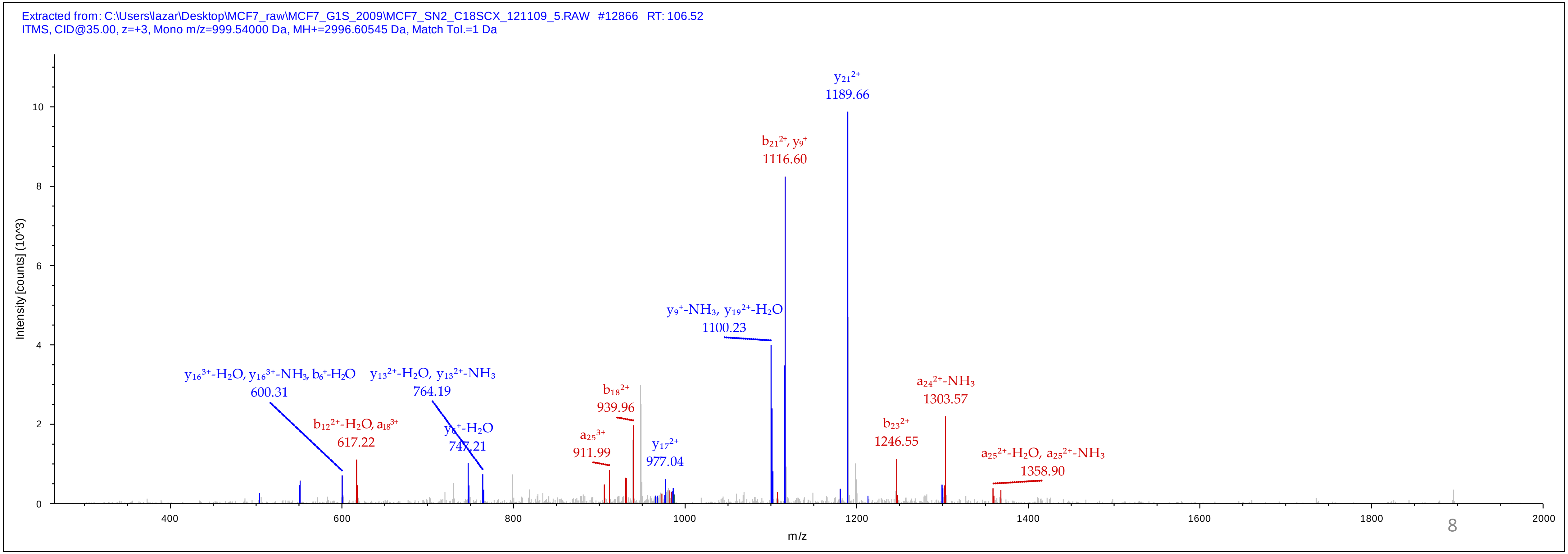

Sequence: CQEKWDKLLLTSTEK, Charge: +2, Monoisotopic m/z: 913.32000 Da (+1846.58 mmu/+2021.84 ppm), MH+: 1825.63272 Da, RT: 115.09 min
Identified with: Sequest HT (v1.3); XCorr:4.05
Fragment match tolerance used for search: 1 Da
Fragments used for search: a; a-H_2O; a-NH_3; b; b-H_2O; b-NH_3; y; y-H_2O; y-NH_3
Protein references (1): c.785A>G p.Y262C|VKRCQEK|Missense|COSMIC|Large intestine(1);Ovary(1)
CANCER_sp_Q7L0Y3,MRRP1_HUMAN Mitochondrial ribonuclease P protein 1

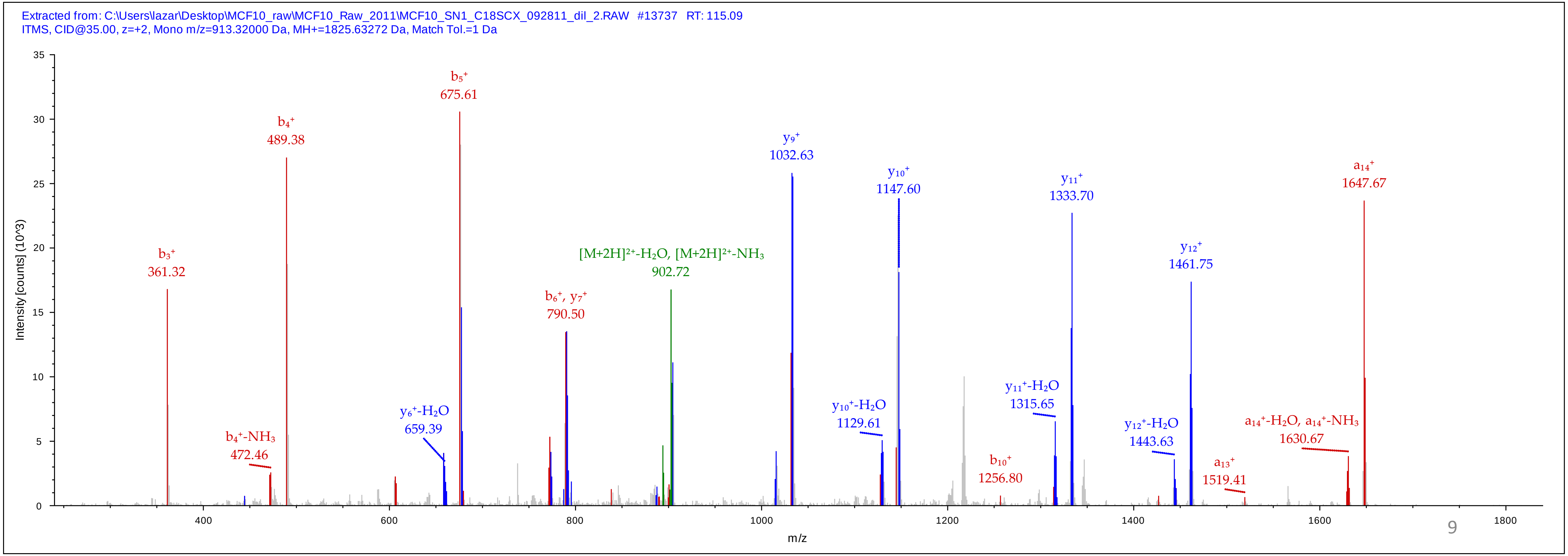

Sequence: DDLQFLADLEELITKFQVFRISQR, Charge: +3, Monoisotopic m/z: 975.06000 Da (-462.43 mmu/-474.25 ppm), MH+: 2923.16545 Da, RT: 199.91 min

Identified with: Sequest HT (v1.3); XCorr:4.35

Fragment match tolerance used for search: 1 Da

Fragments used for search: a; a-H_2O; a-NH_3; b; b-H_2O; b-NH_3; y; y-H_2O; y-NH_3

Protein references (1): c.2913C>A p.H971Q|RISQRSY|Missense|COSMIC|Large intestine(1)

CANCER_sp_Q9Y6X0,SETBP_HUMAN SET-binding protein

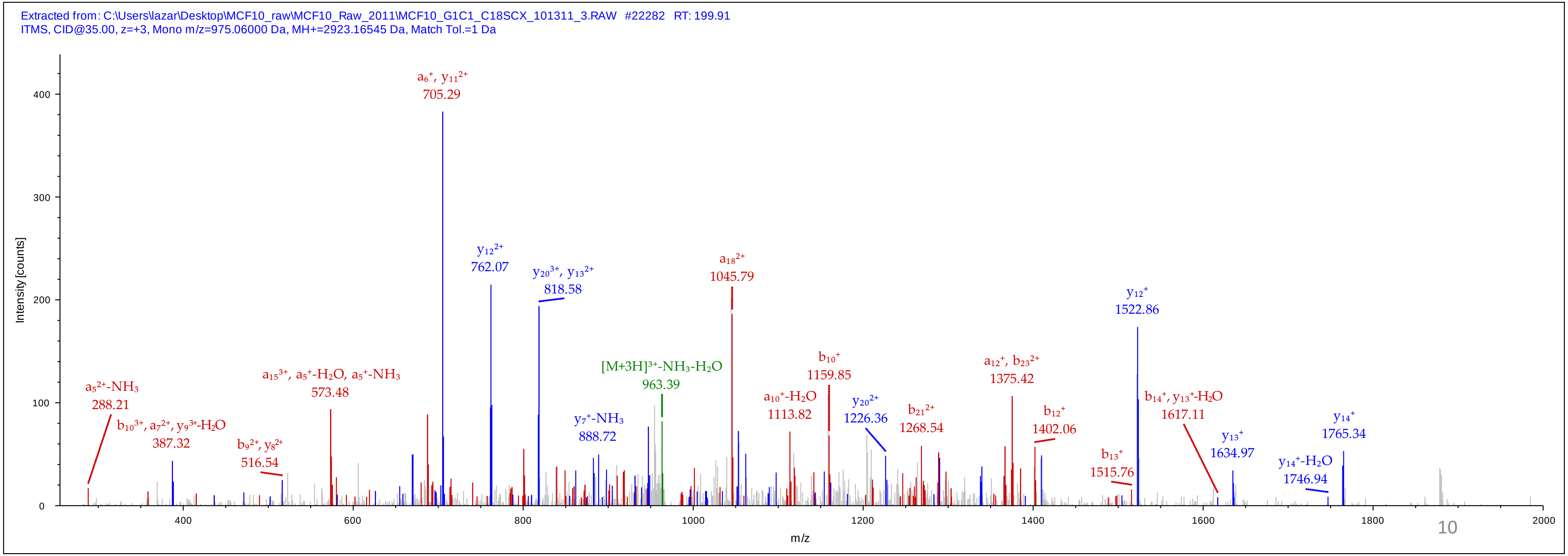

Sequence: DIMTYVSSFYHAFSGAQK, Charge: +2, Monoisotopic m/z: 1026.72000 Da (+239.88 mmu/+233.64 ppm), MH+: 2052.43272 Da, RT: 180.14 min
Identified with: Sequest HT (v1.3); XCorr:3.34
Fragment match tolerance used for search: 1 Da
Fragments used for search: a; a-H_2O; a-NH_3; b; b-H_2O; b-NH_3; y; y-H_2O; y-NH_3
Protein references (1): c.767C>A p.A256D|DEKDIMT|Missense|COSMIC|Ovary(1) CANCER_sp_O43707,ACTN4_HUMAN Alpha-actinin-4

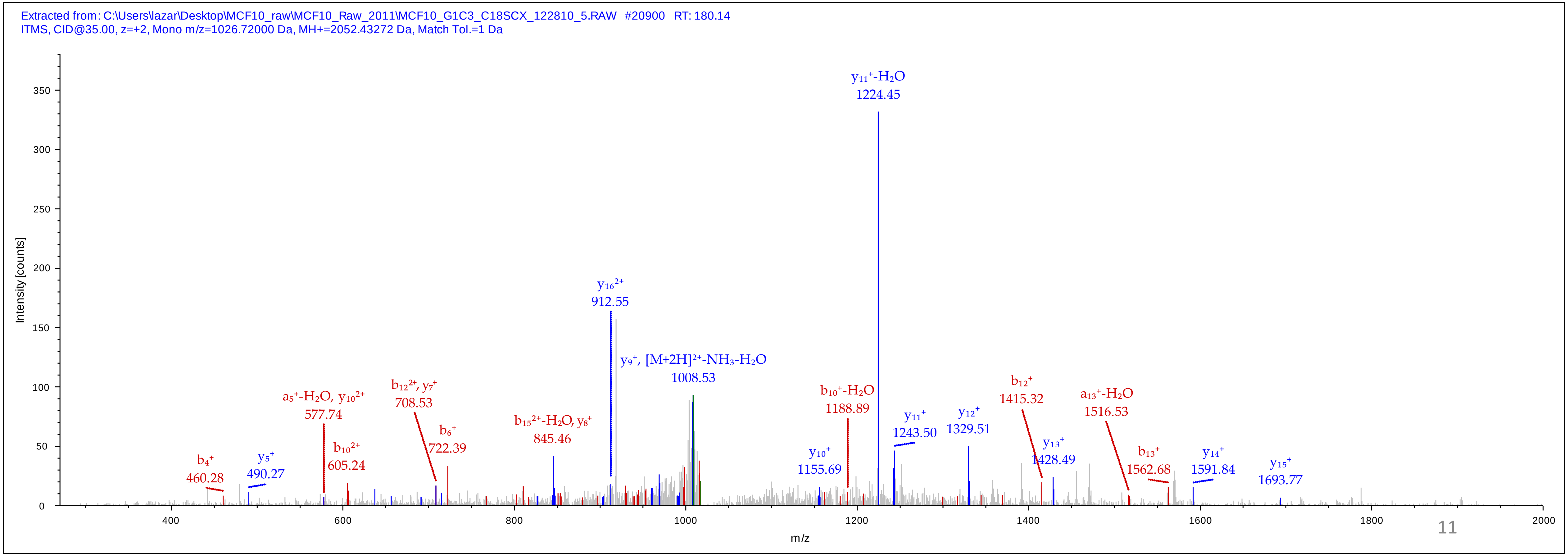

Sequence: DQEGQDLLLFIDNIFR, Charge: +2, Monoisotopic m/z: 968.78000 Da (+285.93 mmu/+295.14 ppm), MH+: 1936.55272 Da, RT: 195.72 min,
Identified with: Sequest HT (v1.3); XCorr:4.66
Fragment match tolerance used for search: 1 Da
Fragments used for search: a; a-H_2O; a-NH_3; b; b-H_2O; b-NH_3; y; y-H_2O; y-NH_3
Protein references (1): c.901G>T p.V301L|GQDLLLF|Missense|COSMIC|Kidney(1) CANCER_sp_P06576,ATPB_HUMAN ATP synthase subunit beta, mitochondrial

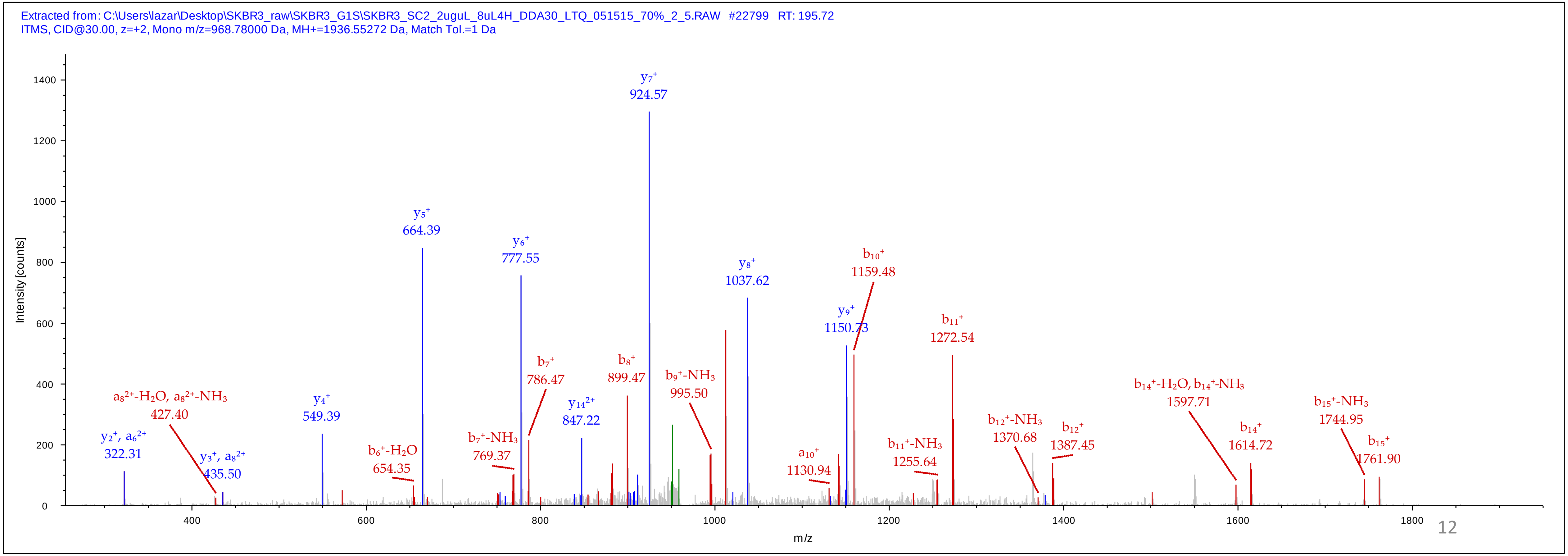

Sequence: EAMQQADDWLGIPQVITPEEIVDPNVDEHSVMTYMSQFPK, Charge: +3, Monoisotopic m/z: 1529.98000 Da (-70.18 mmu/-45.87 ppm), MH+: 4587.92545 Da, RT: 158.82 min
Identified with: Sequest HT (v1.3); XCorr:5.67
Fragment match tolerance used for search: 1 Da
Fragments used for search: a; a-H_2O; a-NH_3; b; b-H_2O; b-NH_3; y; y-H_2O; y-NH_3
Protein references (1): c.781C>A p.L261M|MTYMSQF|Missense|COSMIC|Pancreas(2) CANCER_sp_P21333,FLNA_HUMAN Filamin-A

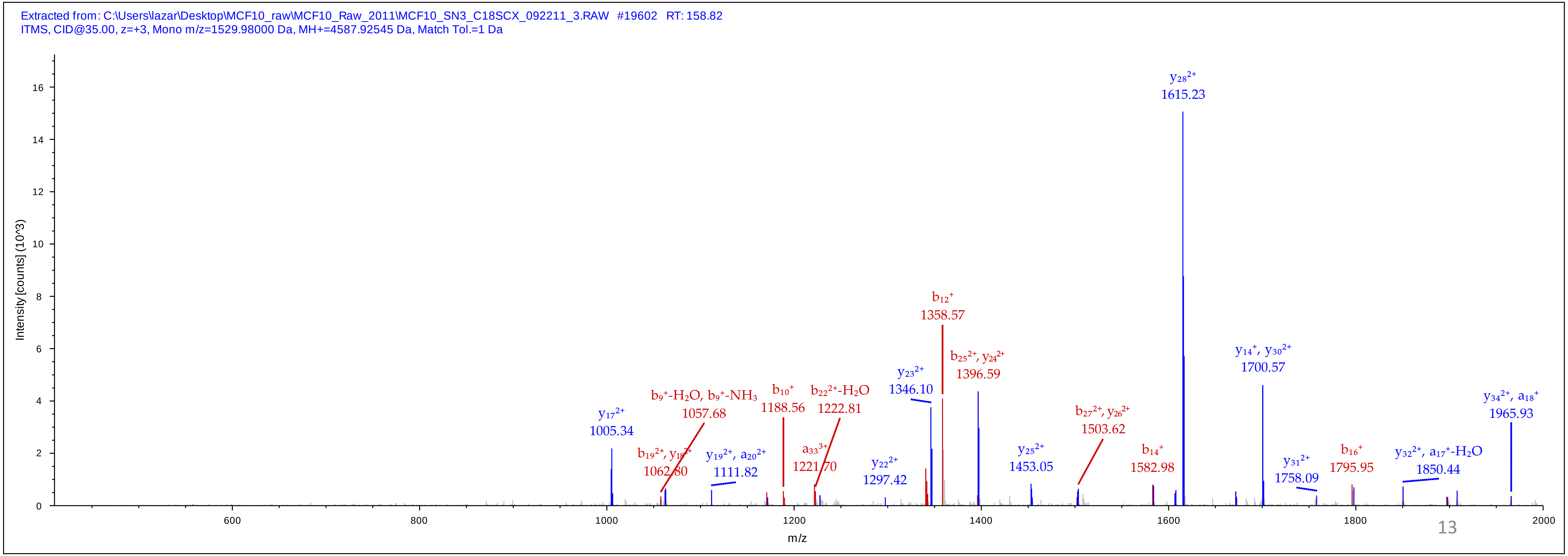

Sequence: EAVMSFSITETEKIK, Charge: +3, Monoisotopic m/z: 571.17108 Da (-460.18 mmu/-805.67 ppm), MH+: 1711.49869 Da, RT: 156.46 min

Identified with: Sequest HT (v1.3); XCorr:4.25

Fragment match tolerance used for search: 1 Da

Fragments used for search: a; a-H_2O; a-NH_3; b; b-H_2O; b-NH_3; y; y-H_2O; y-NH_3

Protein references (1): c.1058A>G p.N353S|AVMSFSI|Missense|COSMIC|Large intestine(1)

CANCER_sp_Q5T4T6,SYC2L_HUMAN Synaptonemal complex protein 2-like

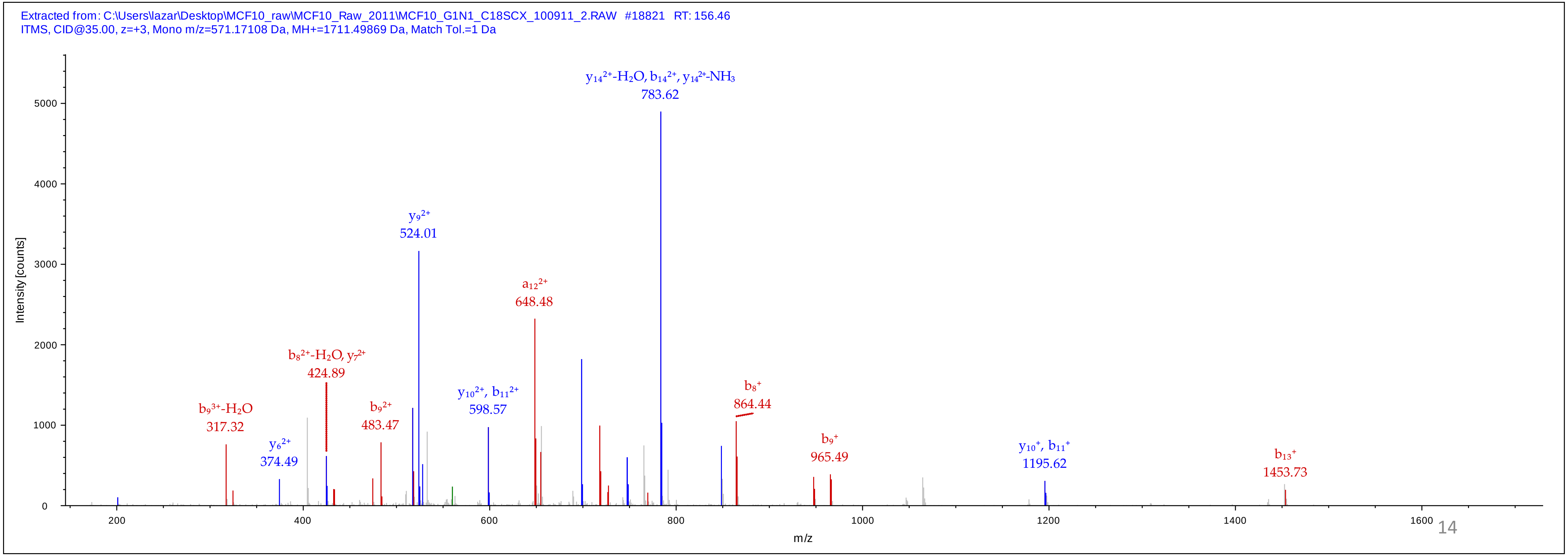

Sequence: EFADSLGIPFLETSAKNAMNVEQSFMTMAAEIK, Charge: +3, Monoisotopic m/z: 1207.28000 Da (-302.46 mmu/-250.53 ppm), MH+: 3619.82545 Da, RT: 204.55 min

Identified with: Sequest HT (v1.3); XCorr:4.59

Fragment match tolerance used for search: 1 Da

Fragments used for search: a; a-H_2O; a-NH_3; b; b-H_2O; b-NH_3; y; y-H_2O; y-NH_3

Protein references (1): c.476C>T p.T159M|KNAMNVE|Missense|COSMIC|Large intestine(1) CANCER_sp_P62820,RAB1A_HUMAN Ras-related protein Rab-1A

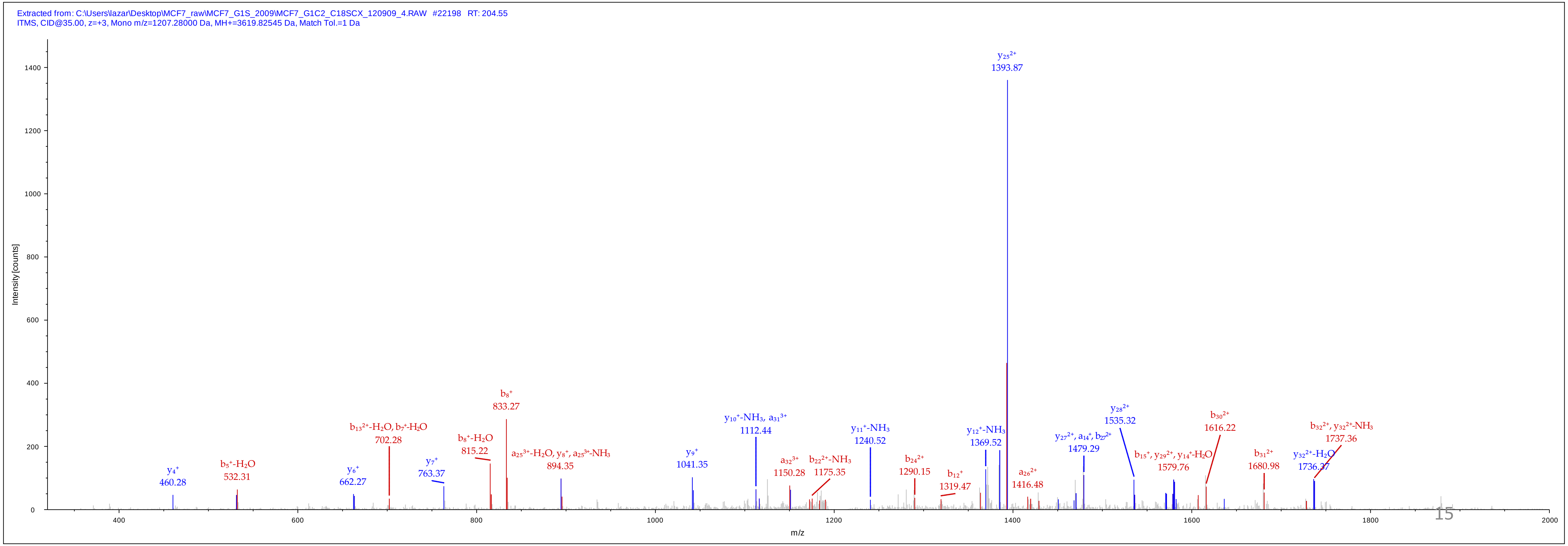

Sequence: EFDTLSGKVEESPDK, Charge: +2, Monoisotopic m/z: 842.64000 Da (+1739.22 mmu/+2064.01 ppm), MH+: 1684.27272 Da, RT: 85.24 min

Identified with: Sequest HT (v1.3); XCorr:3.47

Fragment match tolerance used for search: 1 Da

Fragments used for search: a; a-H_2O; a-NH_3; b; b-H_2O; b-NH_3; y; y-H_2O; y-NH_3

Protein references (1): c.2278T>G p.L760V|SGKVEES|Missense|COSMIC|Breast(1) CANCER_sp_Q9BXX2,AN30B_HUMAN Ankyrin repeat domain-containing protein 30B

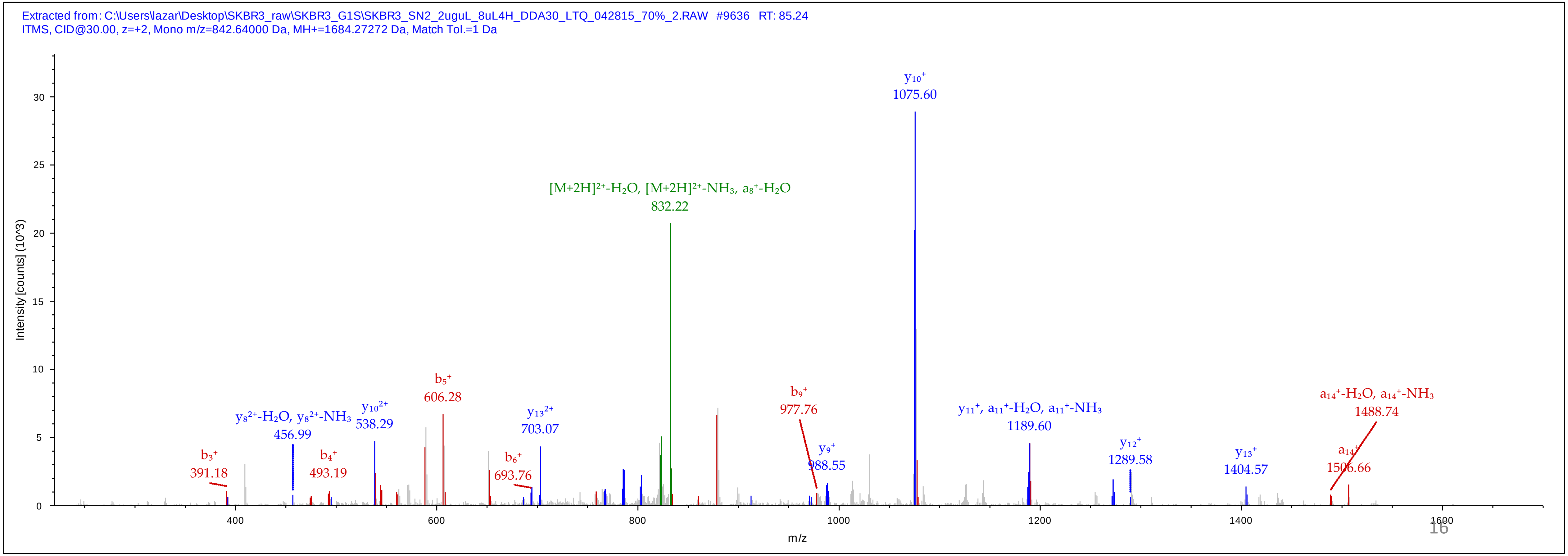

Sequence: EIYPYVIQERRPTLNELGISTPEELGLDKV, Charge: +3, Monoisotopic m/z: 1158.68000 Da (+729.86 mmu/+629.91 ppm), MH+: 3474.02545 Da, RT: 177.58 min

Identified with: Sequest HT (v1.3); XCorr:4.84

Fragment match tolerance used for search: 1 Da

Fragments used for search: a; a-H_2O; a-NH_3; b; b-H_2O; b-NH_3; y; y-H_2O; y-NH_3

Protein references (1): c.389T>G p.L130R|IQERRPT|Missense|COSMIC|Pancreas(1) CANCER_sp_P20674,COX5A_HUMAN Cytochrome c oxidase subunit 5A, mitochondrial

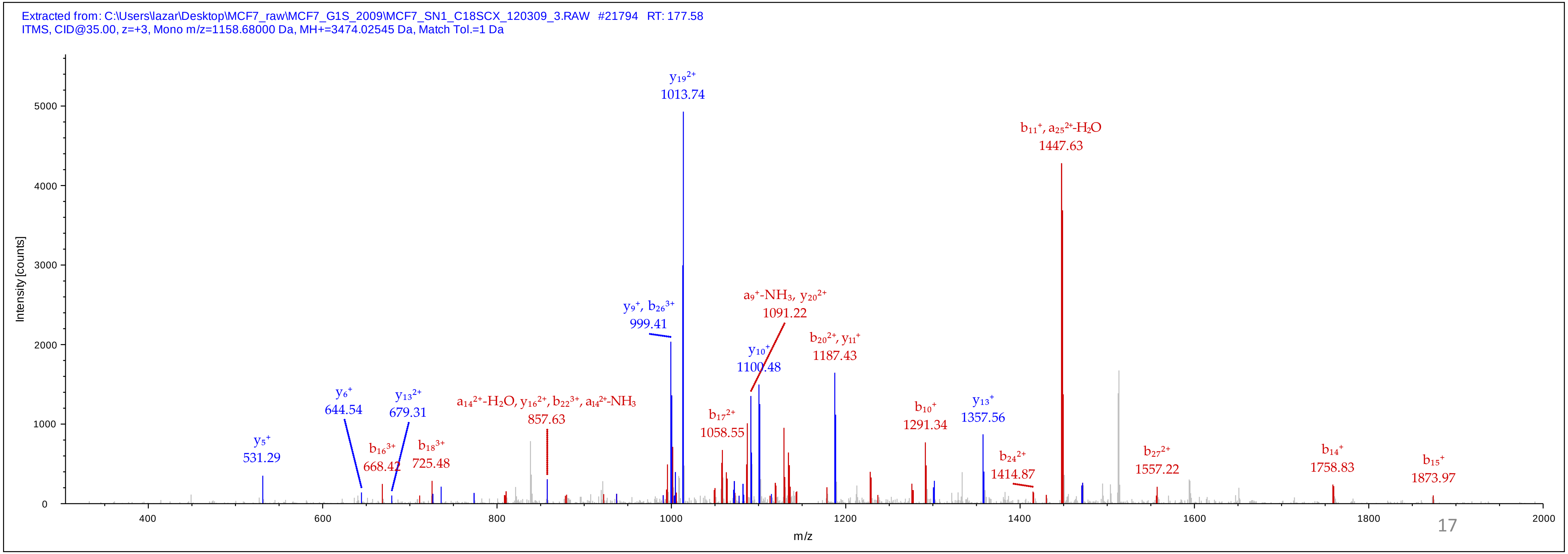

Sequence: EQLQQEQALLEEIER, Charge: +2,   Monoisotopic m/z: 928.82000 Da (+346.71 mmu/+373.28 ppm),   MH+: 1856.63272 Da,   RT: 85.13 min

Identified with: Sequest HT (v1.3); XCorr:4.64

Fragment match tolerance used for search: 1 Da

Fragments used for search: a; a-H_2O; a-NH_3; b; b-H_2O; b-NH_3; y; y-H_2O; y-NH_3

Protein references (1): c.4157G>A p.R1386Q|EQLQQEQ|Missense|COSMIC|Breast(1) OR sp|Q15149-2,PLEC_HUMAN Isoform 2 of Plectin|c.3827G>A|p.R1276Q|EQLQQEQ|Missense|COSMIC|Breast(1) OR sp|Q15149-4,PLEC_HUMAN Isoform 4 of Plectin|c.3746G>A|p.R1249Q|EQLQQEQ|Missense|COSMIC|Breast(1) CANCER_sp_Q15149,PLEC_HUMAN Plectin

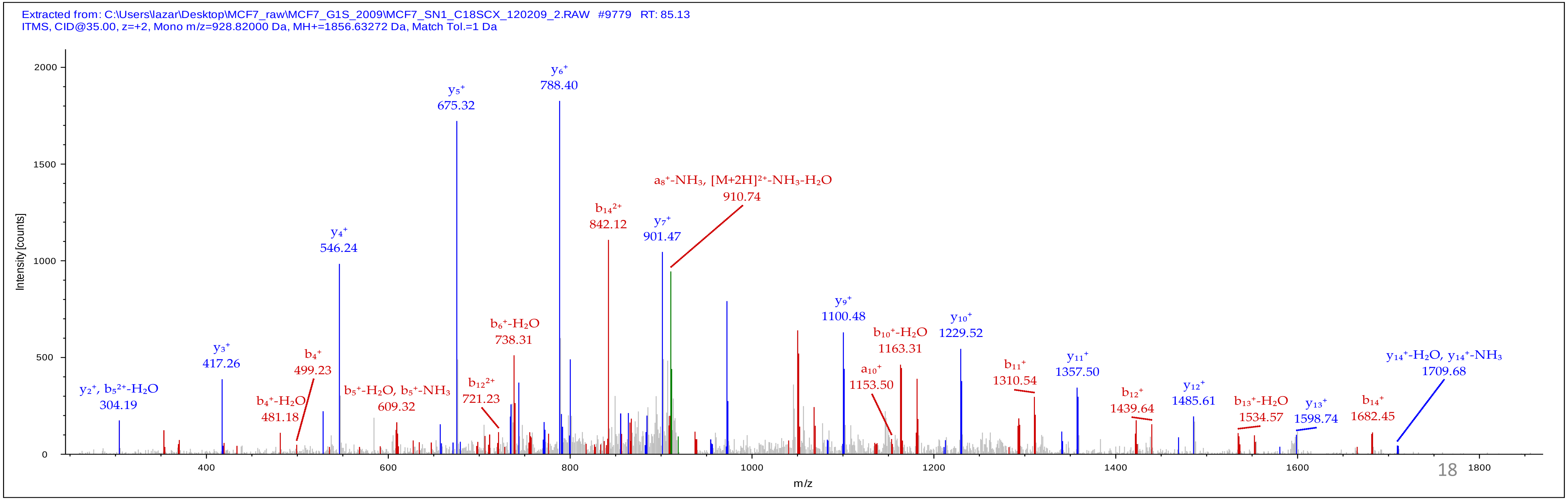

Sequence: FGLAHLMALGLGPWMAVEIPDLIQK, Charge: +3, Monoisotopic m/z: 907.32000 Da (-173.92 mmu/-191.68 ppm), MH+: 2719.94545 Da, RT: 179.00 min

Identified with: Sequest HT (v1.3); XCorr:4.26

Fragment match tolerance used for search: 1 Da

Fragments used for search: a; a-H_2O; a-NH_3; b; b-H_2O; b-NH_3; y; y-H_2O; y-NH_3

Protein references (1): c.436C>A p.L146M|GPWMAVE|Missense|COSMIC|Autonomic ganglia(1)

CANCER_sp_Q9Y3C8,UFC1_HUMAN Ubiquitin-fold modifier-conjugating enzyme 1

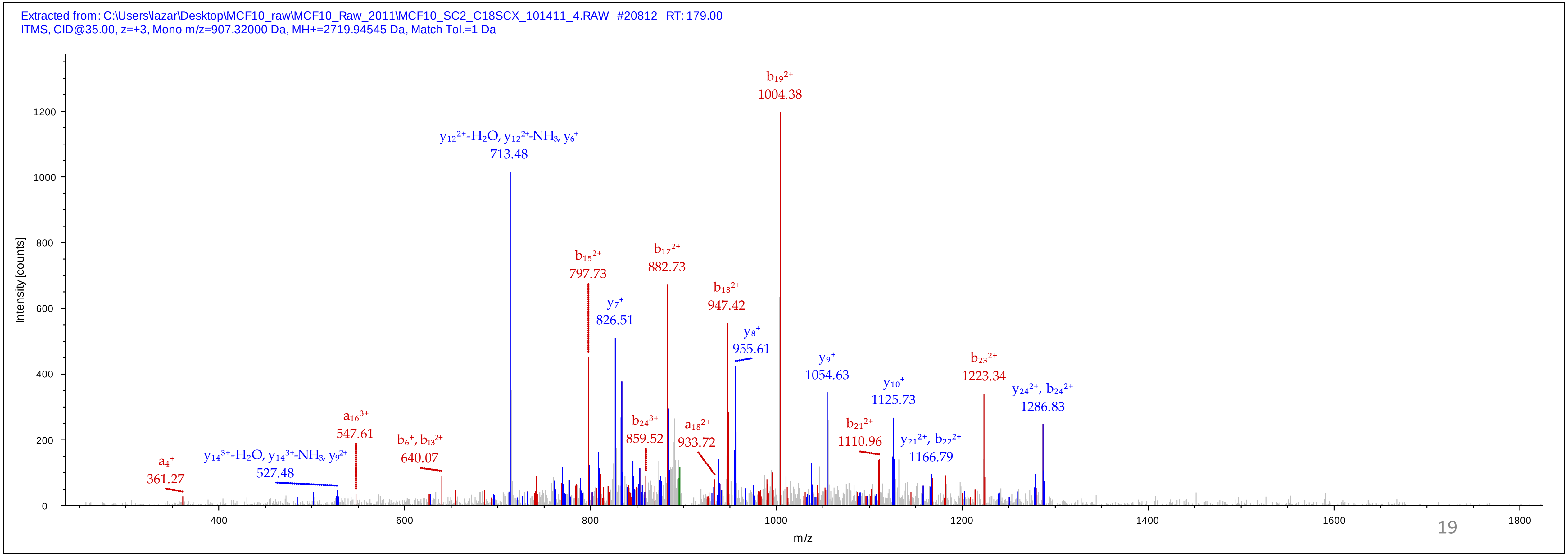

Sequence: FQSSAVMALQEGCEAYLVGLFEDTNLCAIHAK, Charge: +3, Monoisotopic m/z: 1153.30000 Da (-252.69 mmu/-219.1 ppm), MH+: 3457.88545 Da, RT: 199.23 min

Identified with: Sequest HT (v1.3); XCorr:4.71

Fragment match tolerance used for search: 1 Da

Fragments used for search: a; a-H_2O; a-NH_3; b; b-H_2O; b-NH_3; y; y-H_2O; y-NH_3

Protein references (1): c.287C>G p.A96G|LQEGCEA|Missense|COSMIC|Endometrium(1) CANCER_sp_P68431,H31_HUMAN Histone H3.1

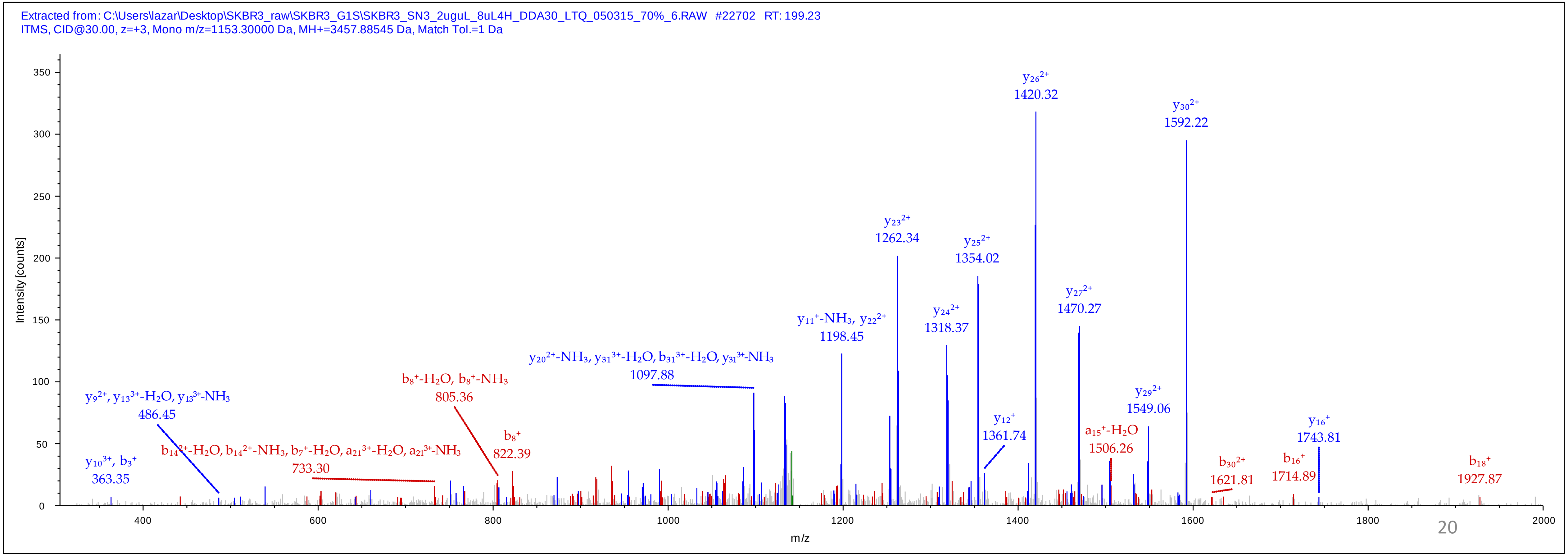

Sequence: FTLDCTHPVEDGIMDAANYEQFLQER, Charge: +3, Monoisotopic m/z: 1015.64000 Da (+848.73 mmu/+835.66 ppm), MH+: 3044.90545 Da, RT: 141.04 min

Identified with: Sequest HT (v1.3); XCorr:4.43

Fragment match tolerance used for search: 1 Da

Fragments used for search: a; a-H_2O; a-NH_3; b; b-H_2O; b-NH_3; y; y-H_2O; y-NH_3

Protein references (1): c.116T>A p.F39Y|AANYEQF|Missense|COSMIC|Endometrium(1) CANCER_sp_P35268,RL22_HUMAN 60S ribosomal protein L22

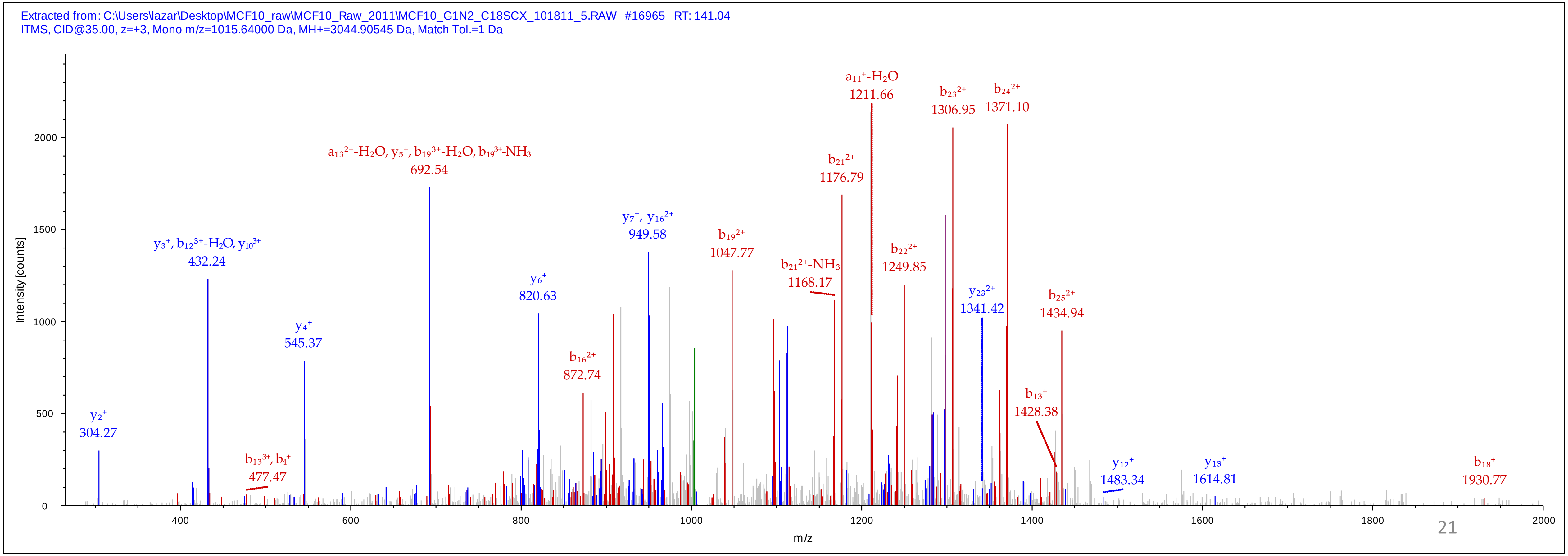

Sequence: GCIEKLSEDVEQLKK, Charge: +2, Monoisotopic m/z: 860.72000 Da (+767.11 mmu/+891.24 ppm), MH+: 1720.43272 Da, RT: 120.55 min
Identified with: Sequest HT (v1.3); XCorr:3.72
Fragment match tolerance used for search: 1 Da
Fragments used for search: a; a-H_2O; a-NH_3; b; b-H_2O; b-NH_3; y; y-H_2O; y-NH_3
Protein references (1): c.157G>T p.V53L|VEQLKKQ|Missense|COSMIC|Lung(1) CANCER_sp_P61266,STX1B_HUMAN Syntaxin-1B

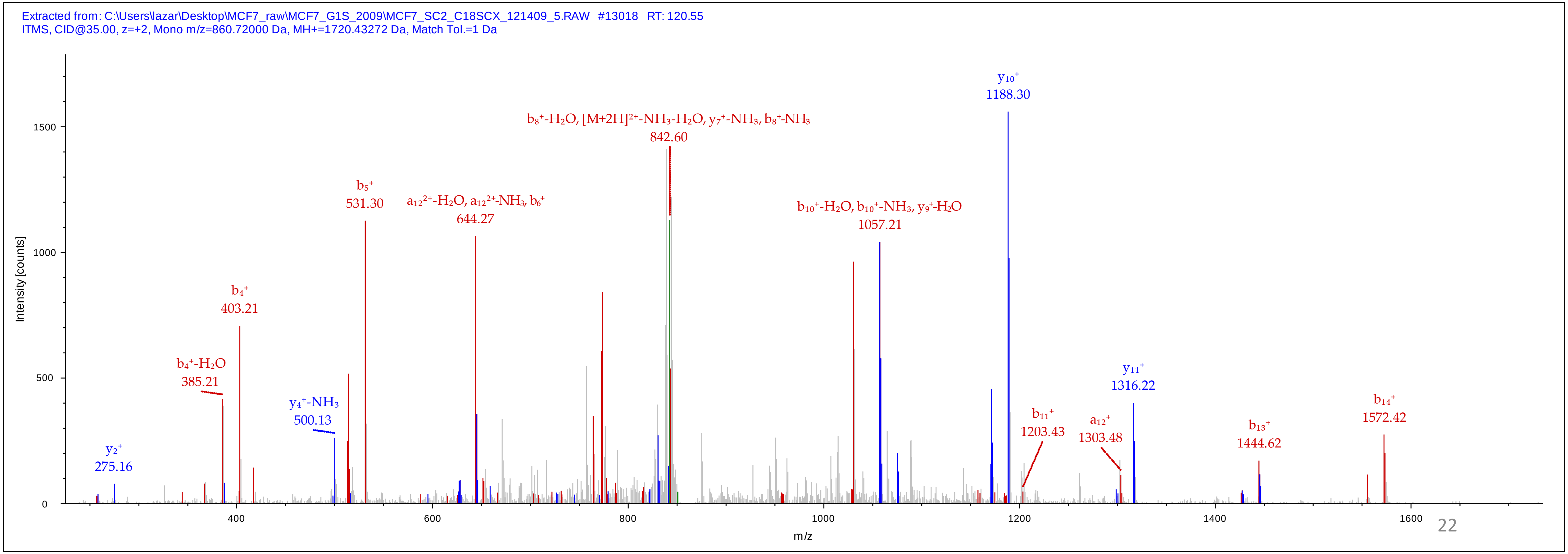

Sequence: GFDFVTFESPADAK, Charge: +2, Monoisotopic m/z: 766.22000 Da (+361.01 mmu/+471.15 ppm), MH+: 1531.43272 Da, RT: 91.71 min
Identified with: Sequest HT (v1.3); XCorr:4.05
Fragment match tolerance used for search: 1 Da
Fragments used for search: a; a-H_2O; b; b-H_2O; y; y-H_2O; y-NH_3
Protein references (1): c.155C>A p.A52D|RGFDFVT|Missense|COSMIC|Endometrium(1) OR sp|P38159,RBMX_HUMAN RNA-binding motif protein, X chromosome|c.155C>A|p.A52D|RGFDFVT|Missense|COSMIC|Endometrium(1) CANCER_sp_P38159-3,RBMX_HUMAN Isoform 3 of RNA-binding motif protein, X chromosome

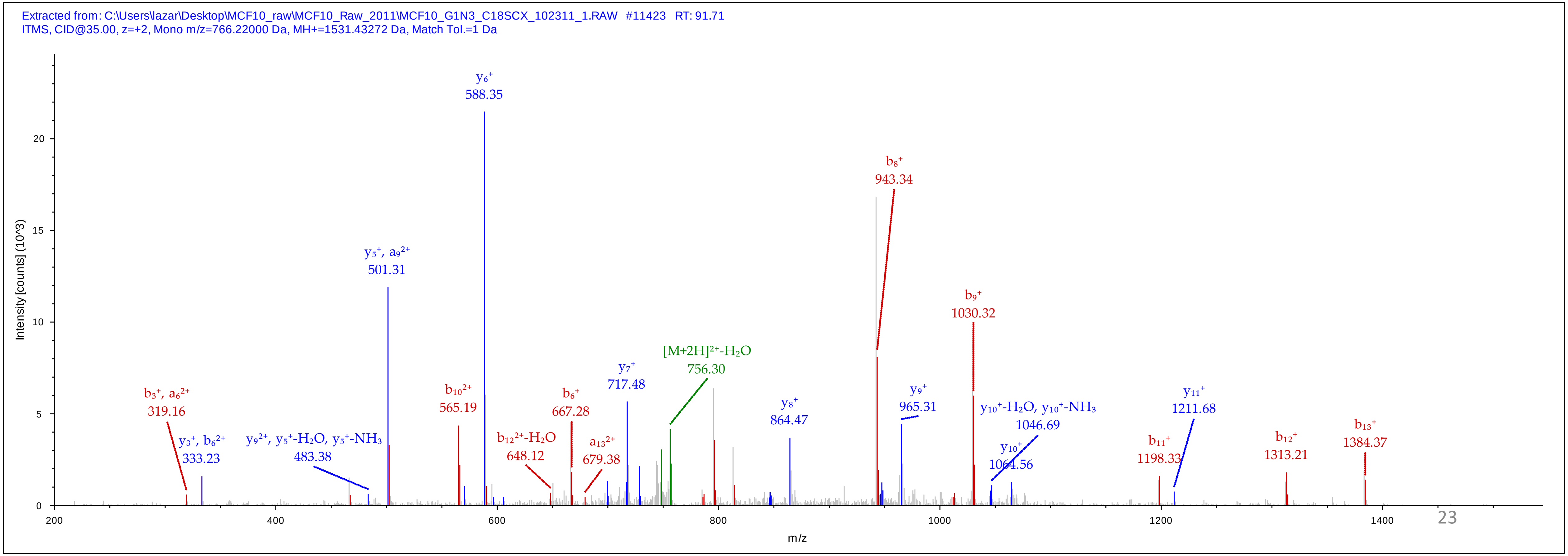

Sequence: GFGFITFTNPEHASDAMR, Charge: +2, Monoisotopic m/z: 999.76000 Da (+297.75 mmu/+297.82 ppm), MH+: 1998.51272 Da, RT: 87.51 min

Identified with: Sequest HT (v1.3); XCorr:4.55

Fragment match tolerance used for search: 1 Da

Fragments used for search: a; a-H_2O; a-NH_3; b; b-H_2O; b-NH_3; y; y-H_2O; y-NH_3

Protein references (1): c.185T>A p.V62D|HASDAMR|Missense|COSMIC|Breast(1) CANCER_sp_P98179,RBM3_HUMAN Putative RNA-binding protein 3

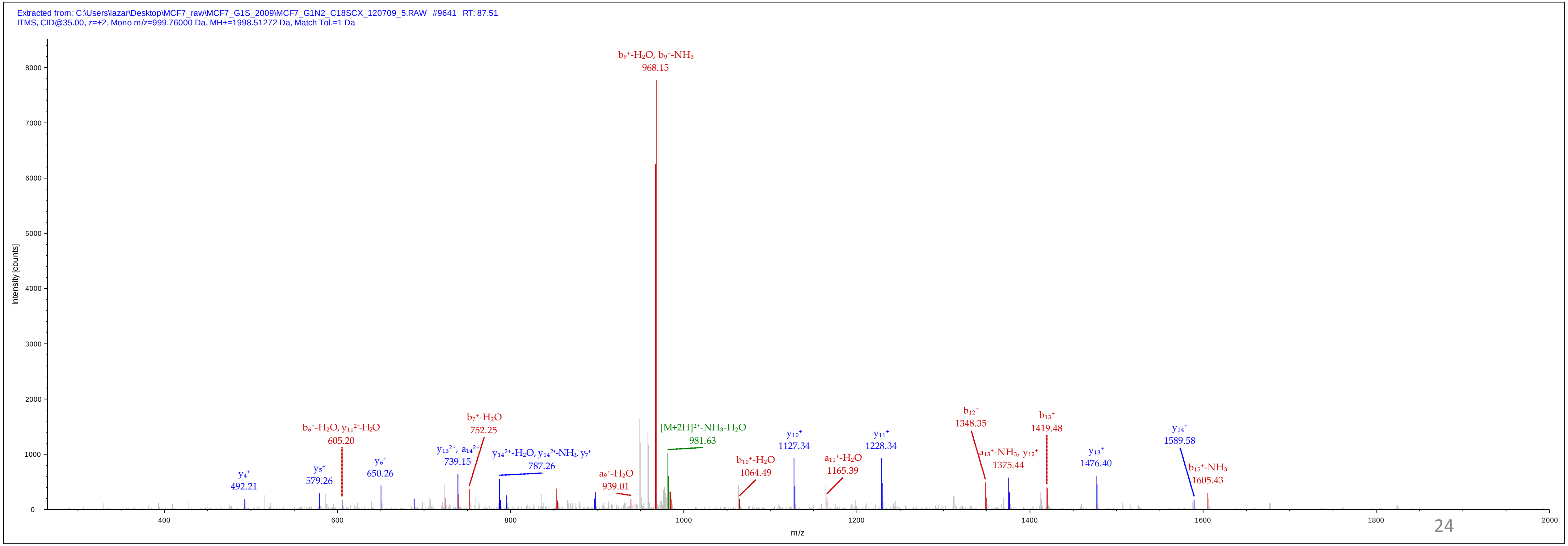

Sequence: GMLDLLEVHLLDFPNIVIK, Charge: +2, Monoisotopic m/z: 1090.40000 Da (+286.67 mmu/+262.91 ppm), MH+: 2179.79272 Da, RT: 138.29 min

Identified with: Sequest HT (v1.3); XCorr:3.89

Fragment match tolerance used for search: 1 Da

Fragments used for search: a; a-H_2O; a-NH_3; b; b-H_2O; b-NH_3; y; y-H_2O; y-NH_3

Protein references (1): c.5612C>T p.P1871L|MLDLLEV|Missense|COSMIC|Kidney(1) CANCER_sp_Q6P2Q9,PRP8_HUMAN Pre-mRNA-processing-splicing factor 8

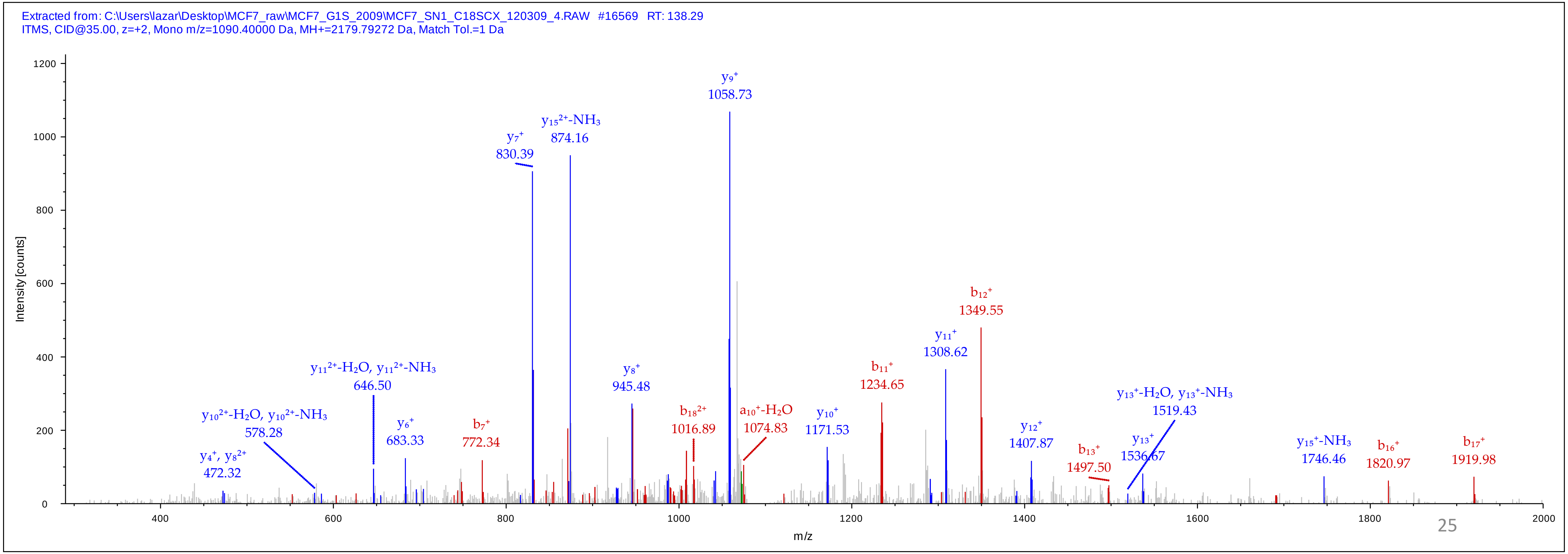

Sequence: GTAAAAAAAAAAAAAK, Charge: +2, Monoisotopic m/z: 614.63226 Da (-203.8 mmu/-331.58 ppm), MH+: 1228.25725 Da, RT: 30.71 min

Identified with: Sequest HT (v1.3); XCorr:5.23

Fragment match tolerance used for search: 1 Da

Fragments used for search: a; a-H_2O; b; b-H_2O; y; y-H_2O; y-NH_3

Protein references (1): p.A159_K160insAAA|AAAAAAKVP|Insertion - in frame|COSMIC|Endometrium(1) - [c.445_446insCTGCTGCTG] CANCER_sp_P50914,RL14_HUMAN 60S ribosomal protein L14

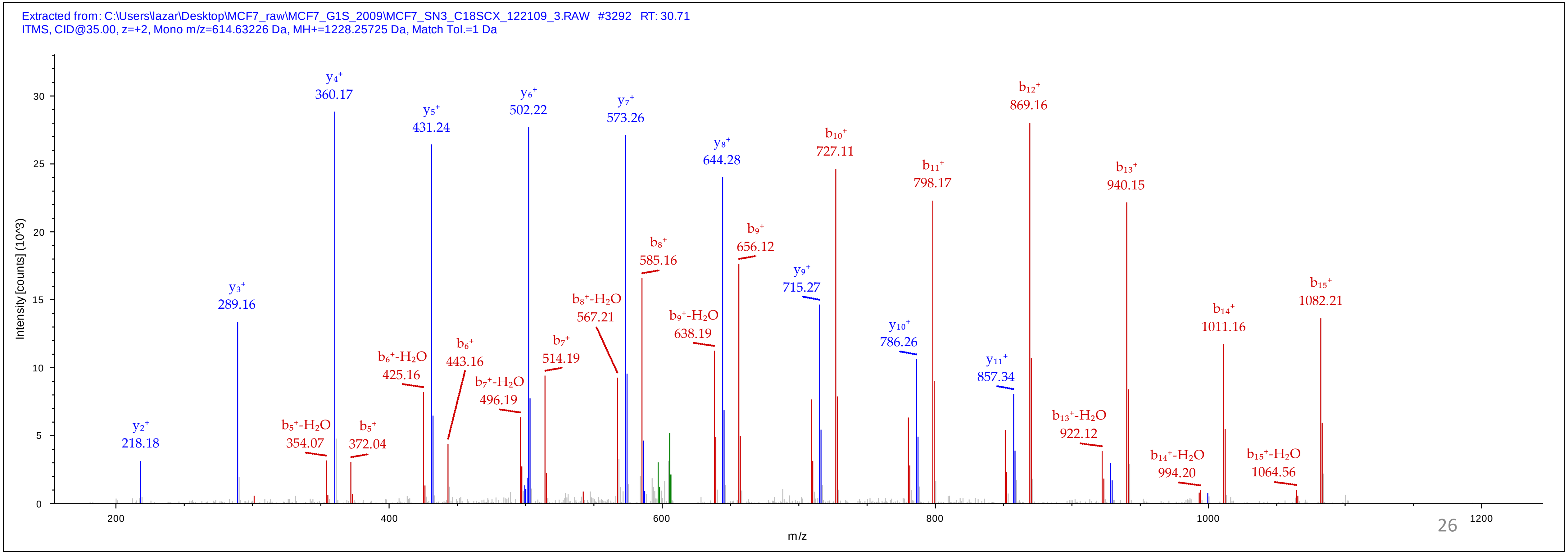

Sequence: HQGVMVGMCQKDSYVGDEAQSK, Charge: +3, Monoisotopic m/z: 799.20000 Da (-493.03 mmu/-616.9 ppm), MH+: 2395.58545 Da, RT: 44.51 min
Identified with: Sequest HT (v1.3); XCorr:4.59
Fragment match tolerance used for search: 1 Da
Fragments used for search: a; a-H_2O; a-NH_3; b; b-H_2O; b-NH_3; y; y-H_2O; y-NH_3
Protein references (1): c.148G>T p.G50C|VGMCQKD|Missense|COSMIC|Ovary(1) CANCER_sp_P68133,ACTS_HUMAN Actin, alpha skeletal muscle

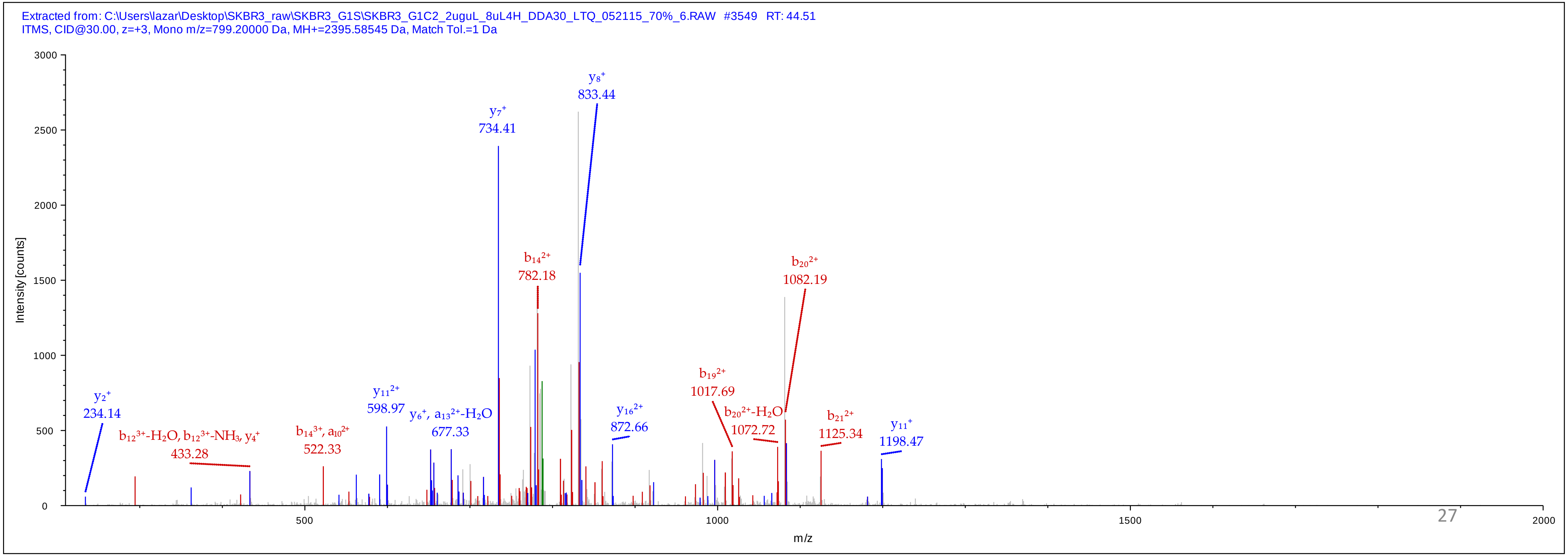

Sequence: HRILPEKYPPPTELLDLQPLPVSALR, Charge: +3, Monoisotopic m/z: 998.86000 Da (+622.78 mmu/+623.49 ppm), MH+: 2994.56545 Da, RT: 141.61 min

Identified with: Sequest HT (v1.3); XCorr:4.56

Fragment match tolerance used for search: 1 Da

Fragments used for search: a; a-H_2O; a-NH_3; b; b-H_2O; b-NH_3; y; y-H_2O; y-NH_3

Protein references (1): c.3866T>G p.L1289R|FRHRILP|Missense|COSMIC|Large intestine(1) CANCER_sp_O75643,U520_HUMAN U5 small nuclear ribonucleoprotein 200 kDa helicase

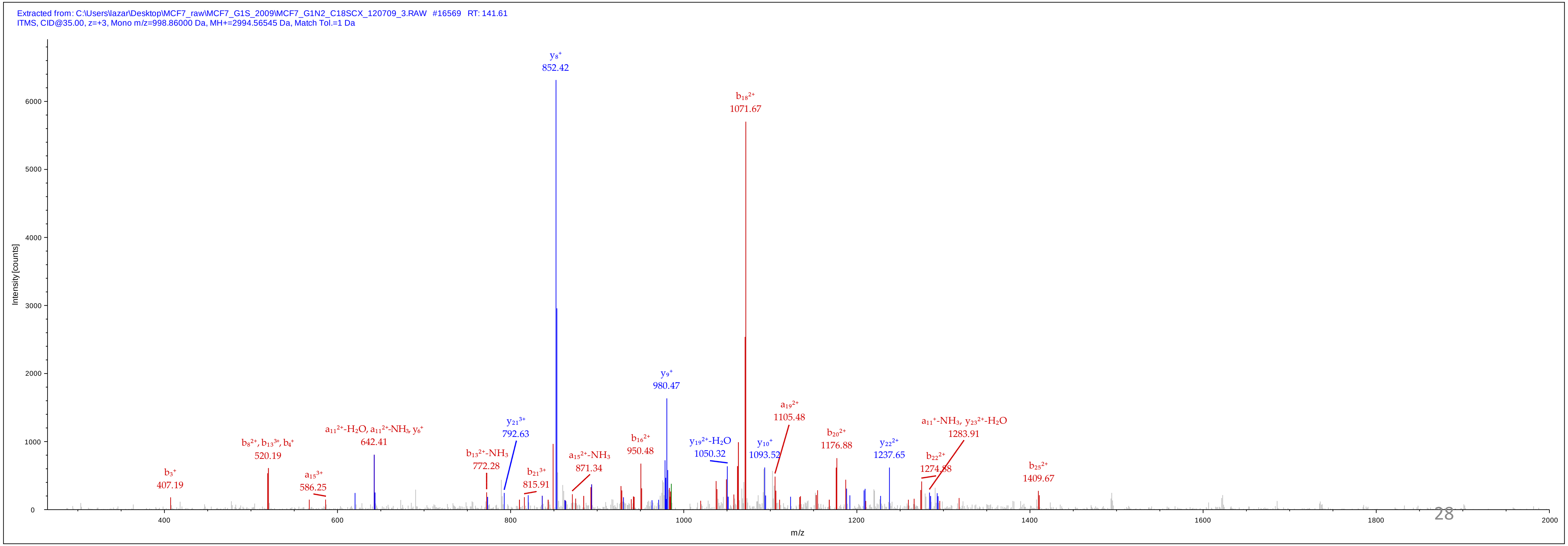

Sequence: IMSLVDPNHCGLVTFQAFIDFMSR, Charge: +3, Monoisotopic m/z: 915.92000 Da (+1474.58 mmu/+1609.94 ppm), MH+: 2745.74545 Da, RT: 171.51 min

Identified with: Sequest HT (v1.3); XCorr:4.93

Fragment match tolerance used for search: 1 Da

Fragments used for search: a; a-H_2O; a-NH_3; b; b-H_2O; b-NH_3; y; y-H_2O; y-NH_3

Protein references (1): c.2467A>T p.S823C|PNHCGLV|Missense|COSMIC|Lung(2) CANCER_sp_O43707,ACTN4_HUMAN Alpha-actinin-4

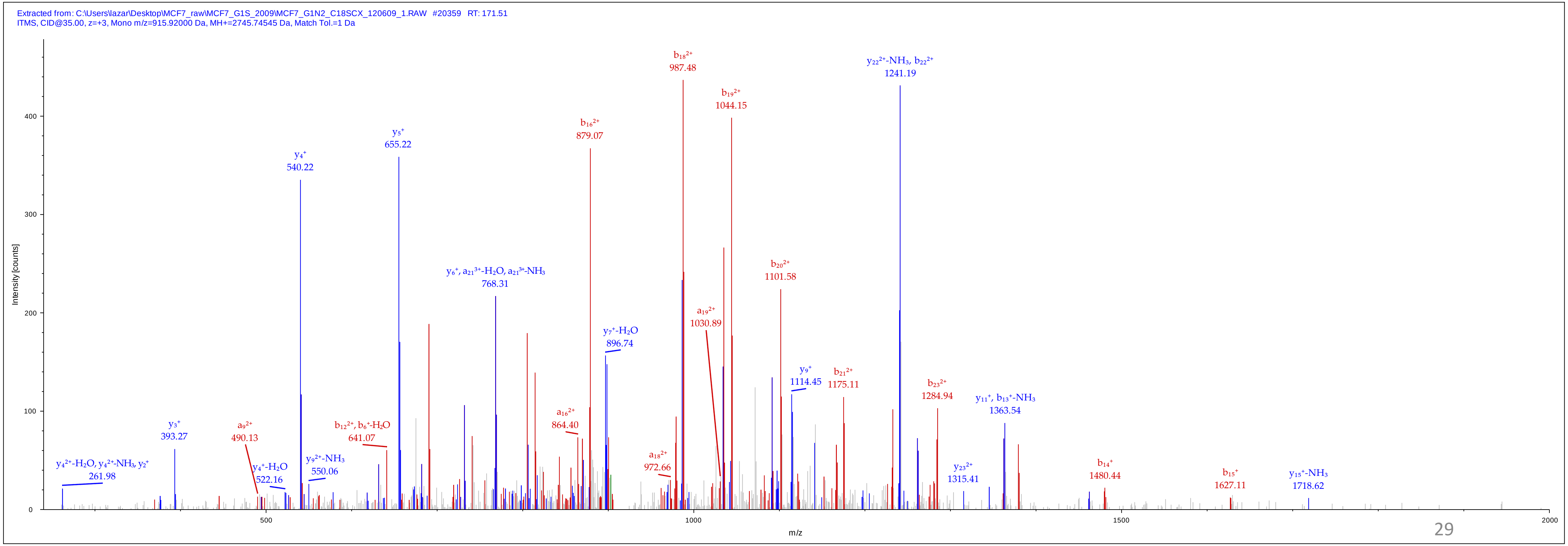

Sequence: KHTLSFVDVGTGK, Charge: +2, Monoisotopic m/z: 694.70087 Da (-179.55 mmu/-258.46 ppm), MH+: 1388.39446 Da, RT: 33.85 min
Identified with: Sequest HT (v1.3); XCorr:3.87
Fragment match tolerance used for search: 1 Da
Fragments used for search: a; a-H_2O; a-NH_3; b; b-H_2O; b-NH_3; y; y-H_2O; y-NH_3
Protein references (1): c.1886A>T p.Y629F|TLSFVDV|Missense|COSMIC|Adrenal gland(1) CANCER_sp_P31040,DHSA_HUMAN Succinate dehydrogenase [ubiquinone] flavoprotein subunit, mitochondrial

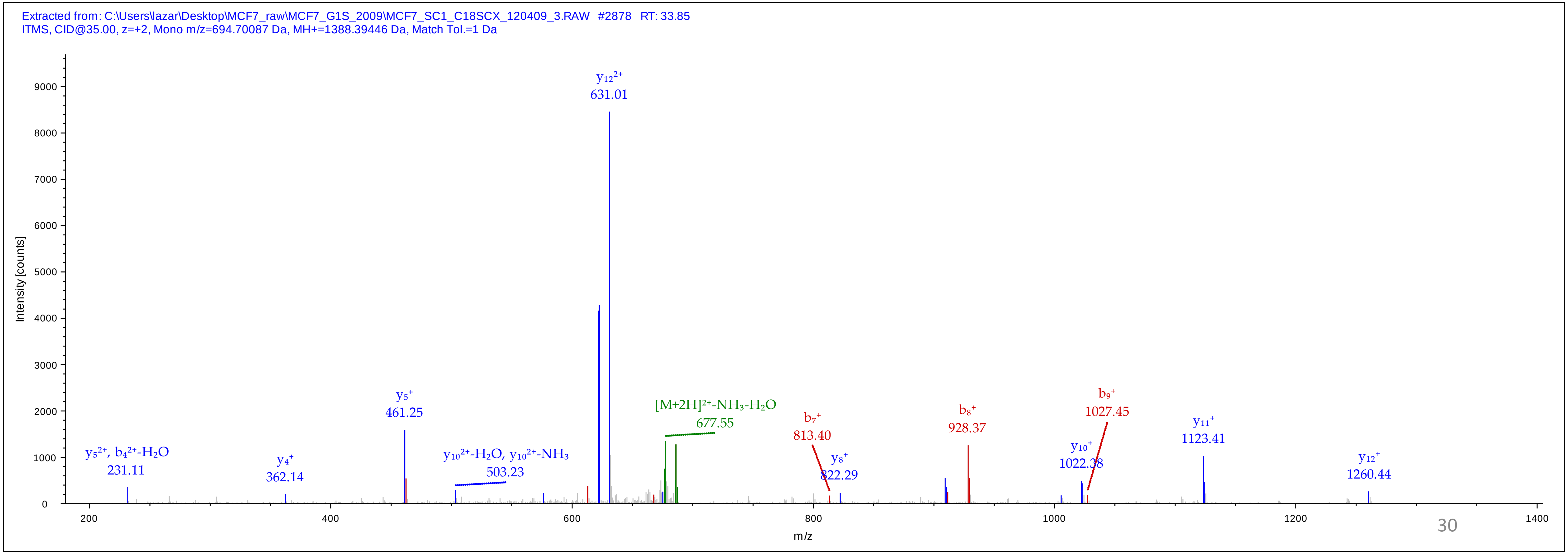

Sequence: KSQESLTENPSETLKPATSISSTSQTKGINVK, Charge: +4, Monoisotopic m/z: 847.12000 Da (-1326.64 mmu/-1566.05 ppm), MH+: 3385.45817 Da, RT: 182.97 min
Identified with: Sequest HT (v1.3); XCorr:4.22
Fragment match tolerance used for search: 1 Da
Fragments used for search: a; a-H_2O; a-NH_3; b; b-H_2O; b-NH_3; y; y-H_2O; y-NH_3
Protein references (1): c.5204T>C p.I1735T|ISSTSQT|Missense|COSMIC|Large intestine(1)
CANCER_tr_F5GXV7,F5GXV7_HUMAN Neurobeachin

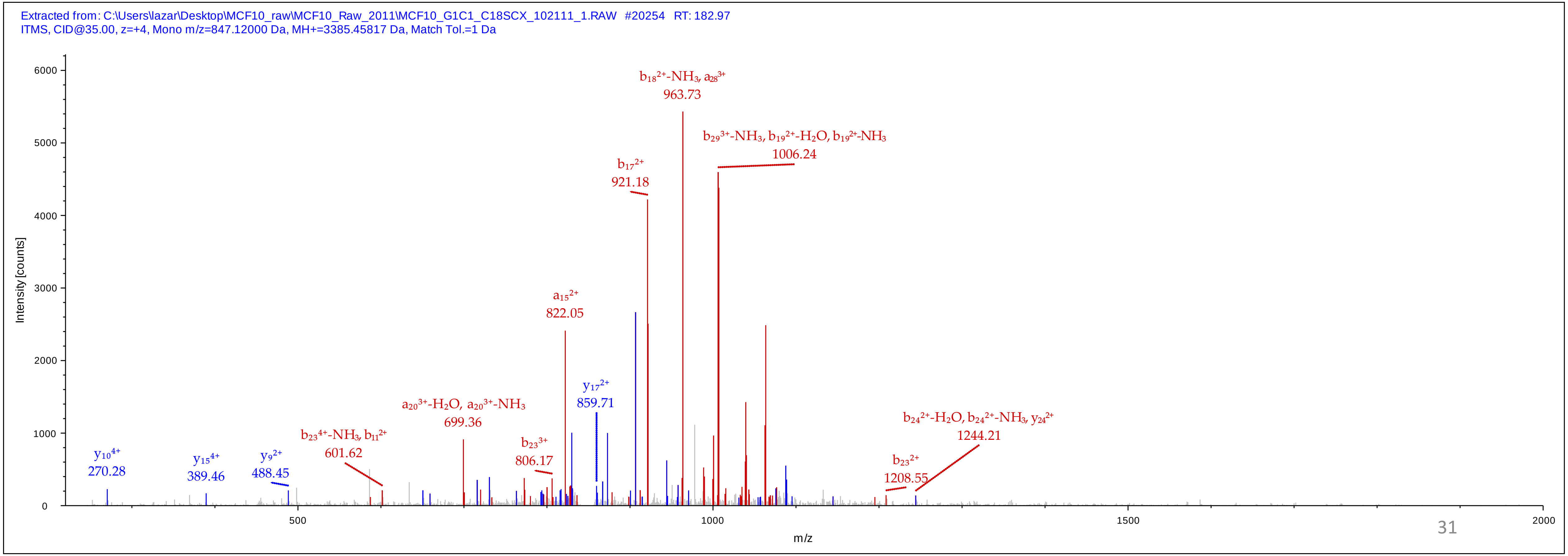

Sequence: LCGLLVLGSWCISVMGFLLETLTILR, Charge: +3, Monoisotopic m/z: 951.30000 Da (+438.94 mmu/+461.41 ppm), MH+: 2851.88545 Da, RT: 191.79 min

Identified with: Sequest HT (v1.3); XCorr:4.25

Fragment match tolerance used for search: 1 Da

Fragments used for search: a; a-H_2O; b; b-H_2O; y; y-H_2O; y-NH_3

Protein references (1): c.467C>T p.S156F|VMGFLLE|Missense|COSMIC|Kidney(1) CANCER_sp_O60412,OR7C2_HUMAN Olfactory receptor 7C2

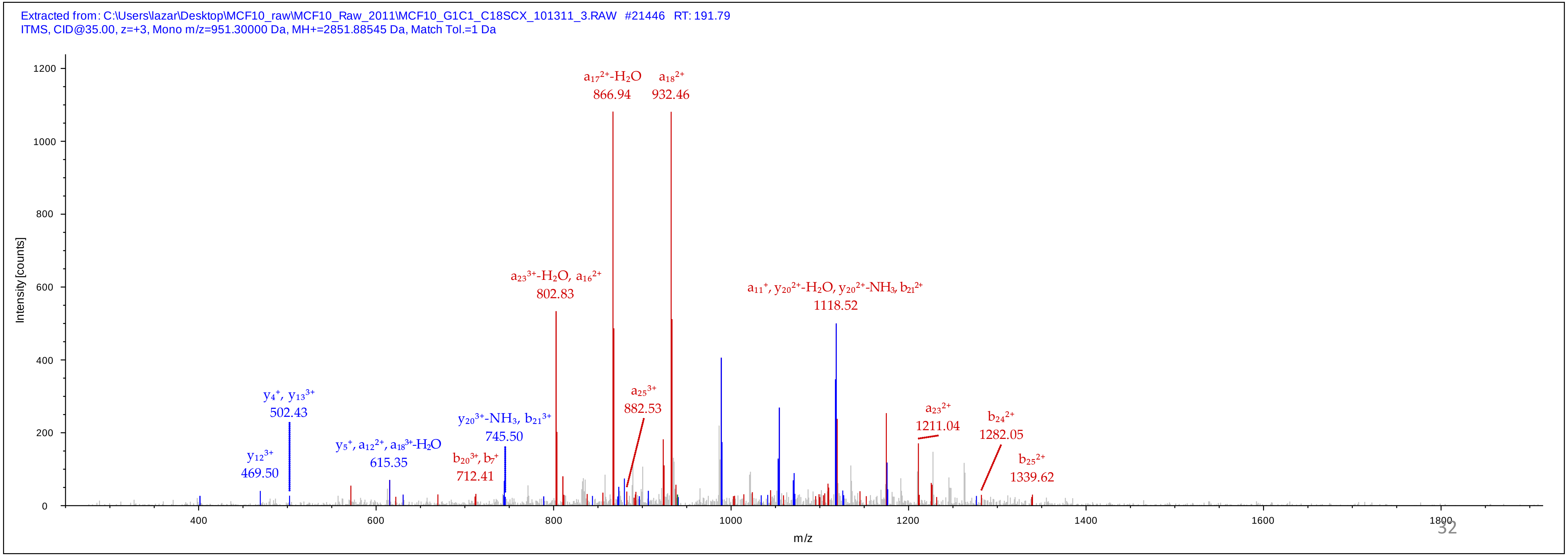

Sequence: LCYVALYFEQEMATAASSSSLEK, Charge: +2, Monoisotopic m/z: 1271.78000 Da (+682.21 mmu/+536.42 ppm), MH+: 2542.55272 Da, RT: 128.71 min

Identified with: Sequest HT (v1.3); XCorr:4.33

Fragment match tolerance used for search: 1 Da

Fragments used for search: a; a-H_2O; a-NH_3; b; b-H_2O; b-NH_3; y; y-H_2O; y-NH_3

Protein references (1): c.2764G>T p.D922Y|VALYFEQ|Missense|COSMIC|Endometrium(1);Lung(1)

CANCER_sp_Q6S8J3,POTEE_HUMAN POTE ankyrin domain family member E

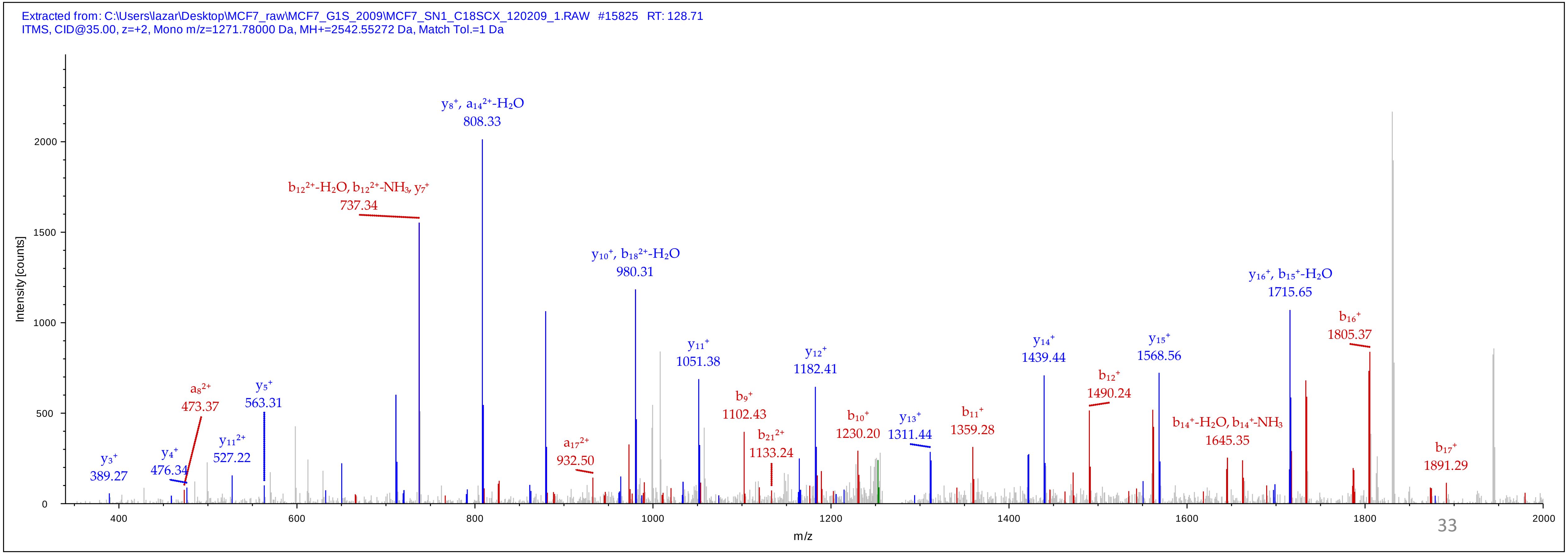

Sequence: LDTNSDGQLDYSEFLNLIGGLAMACHDSFLK, Charge: +3, Monoisotopic m/z: 1131.08000 Da (+1213.43 mmu/+1072.81 ppm), MH+: 3391.22545 Da, RT: 210.16 min

Identified with: Sequest HT (v1.3); XCorr:4.03

Fragment match tolerance used for search: 1 Da

Fragments used for search: a; a-H_2O; a-NH_3; b; b-H_2O; b-NH_3; y; y-H_2O; y-NH_3

Protein references (1): c.230T>A p.F77Y|QLDYSEF|Missense|COSMIC|Ovary(1) CANCER_sp_P31949,S10AB_HUMAN Protein S100-A11

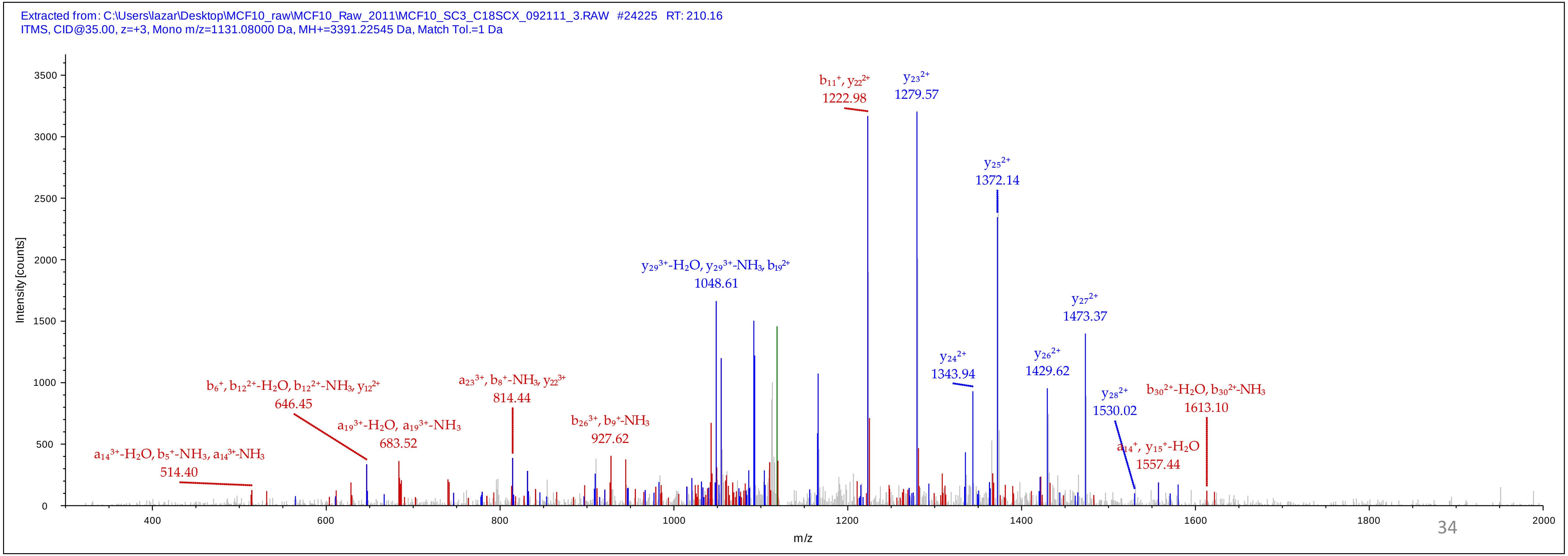

Sequence: LFDHLESPTPNPTEPLFLAQAEVYK, Charge: +3,   Monoisotopic m/z: 952.94000 Da (+120.05 mmu/+125.98 ppm), MH+: 2856.80545 Da,   RT: 108.60 min

Identified with: Sequest HT (v1.3); XCorr:5.80

Fragment match tolerance used for search: 1 Da

Fragments used for search: a; a-H_2O; a-NH_3; b; b-H_2O; b-NH_3; y; y-H_2O; y-NH_3

Protein references (1): c.2915C>T p.P972L|FDHLESP|Missense|COSMIC|Lung(1) CANCER_sp_P49327,FAS_HUMAN Fatty acid synthase

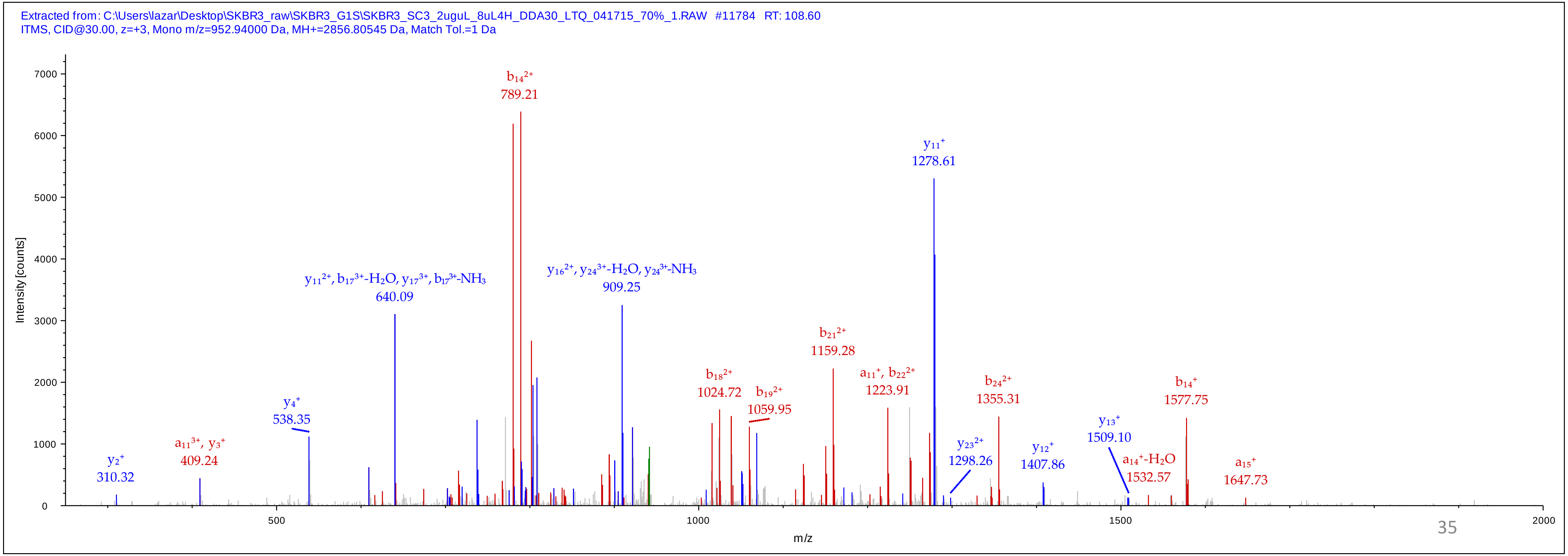

Sequence: LFLASLAAAGSGTDAQVALENEVK, Charge: +3, Monoisotopic m/z: 792.46000 Da (+40.05 mmu/+50.53 ppm), MH+: 2375.36545 Da, RT: 208.31 min

Identified with: Sequest HT (v1.3); XCorr:4.11

Fragment match tolerance used for search: 1 Da

Fragments used for search: a; a-H_2O; a-NH_3; b; b-H_2O; b-NH_3; y; y-H_2O; y-NH_3

Protein references (1): c.6458T>A p.V2153E|VALENEV|Missense|COSMIC|Lung(1) OR co|HUWE1|c.6047T>A|p.V2016E|VALENEV|Missense|COSMIC|Lung(1) CANCER_sp_Q7Z6Z7,HUWE1_HUMAN E3 ubiquitin-protein ligase HUWE1

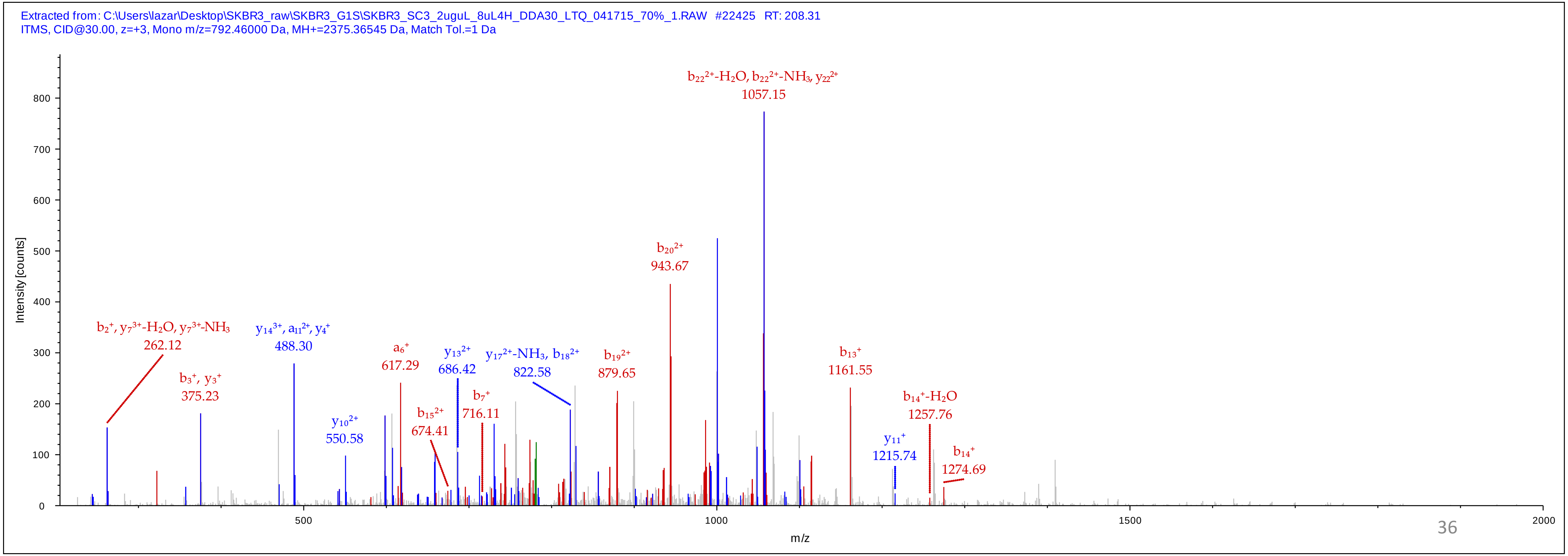

Sequence: LTENLSALQR, Charge: +2, Monoisotopic m/z: 574.58000 Da (+1761.85 mmu/+3066.33 ppm), MH+: 1148.15272 Da, RT: 61.66 min
Identified with: Sequest HT (v1.3); XCorr:3.77
Fragment match tolerance used for search: 1 Da
Fragments used for search: a; a-H_2O; a-NH_3; b; b-H_2O; b-NH_3; y; y-H_2O; y-NH_3

Protein references (1): c.1193G>A p.R398Q|SALQRLQ|Missense|COSMIC|Prostate(2) CANCER_sp_Q8TD16-2,BICD2_HUMAN Isoform 2 of Protein bicaudal D homolog 2

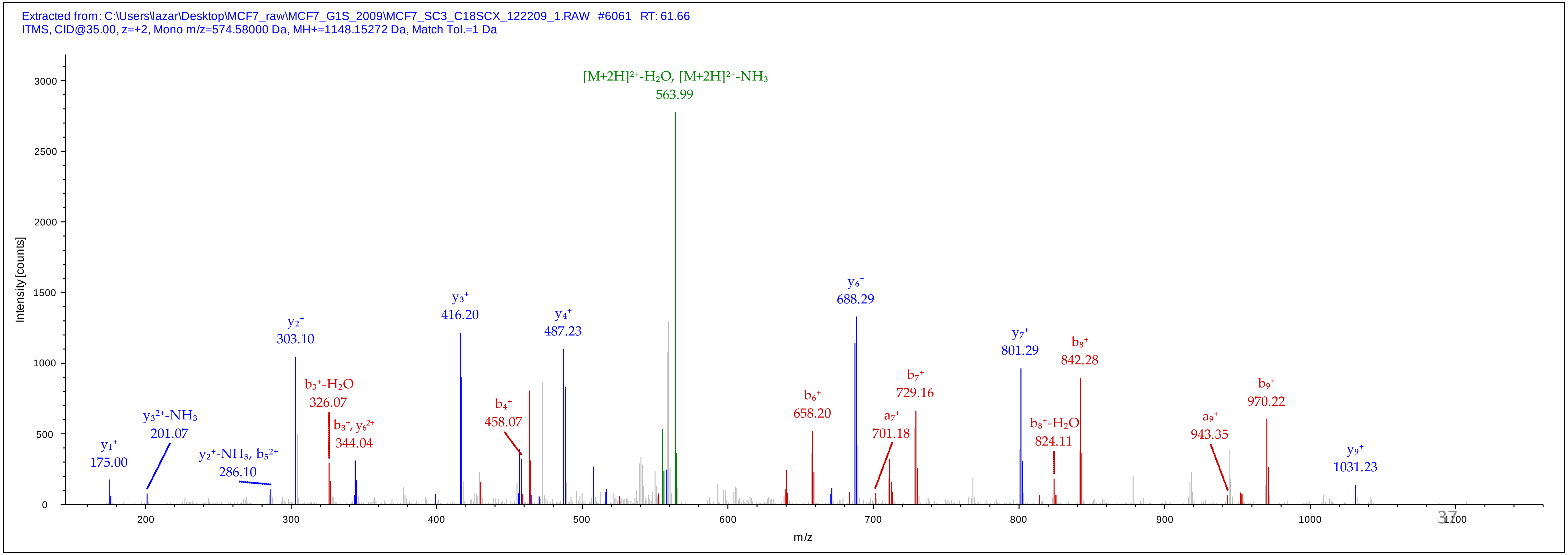

Sequence: LTQAQIFDYSEIPNFPR, Charge: +2, Monoisotopic m/z: 1020.34000 Da (+324.85 mmu/+318.38 ppm), MH+: 2039.67272 Da, RT: 102.09 min

Identified with: Sequest HT (v1.3); XCorr:4.37

Fragment match tolerance used for search: 1 Da

Fragments used for search: a; a-H_2O; a-NH_3; b; b-H_2O; b-NH_3; y; y-H_2O; y-NH_3

Protein references (1): c.151G>A p.G51S|FDYSEIP|Missense|COSMIC|Stomach(1) CANCER_sp_P00491,PNPH_HUMAN Purine nucleoside phosphorylase

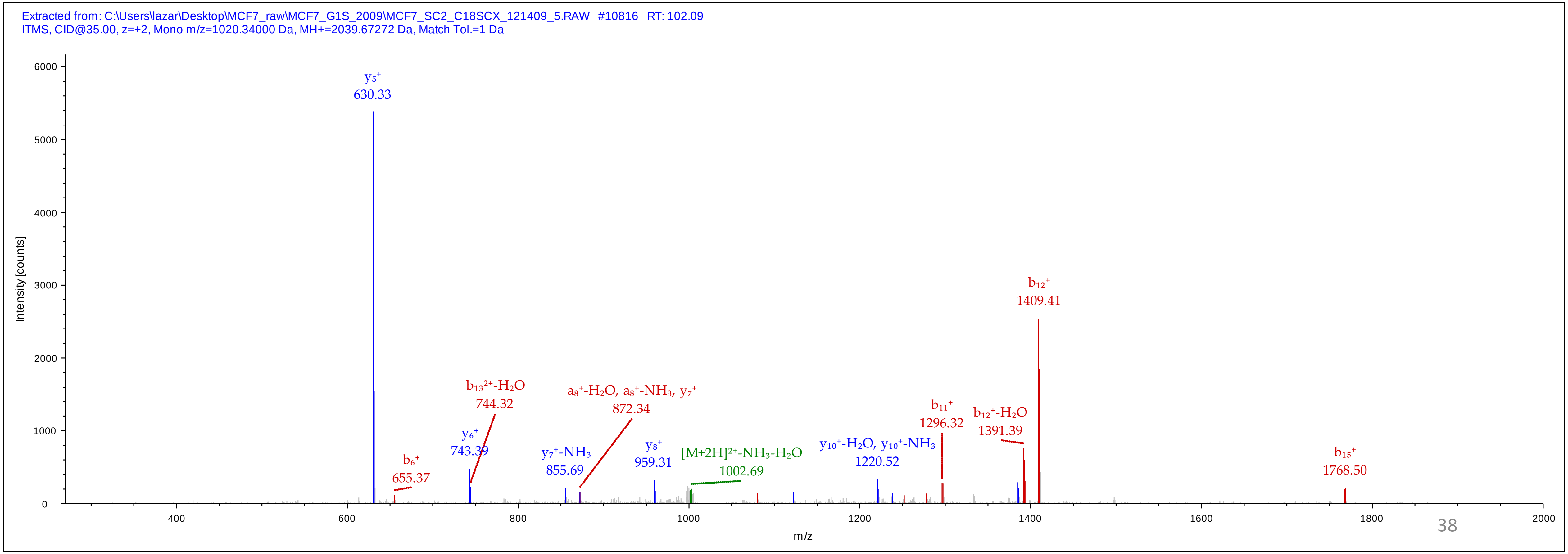

Sequence: MDATFIGNSTAIQELFK, Charge: +2, Monoisotopic m/z: 943.24000 Da (-232.02 mmu/-245.99 ppm), MH+: 1885.47272 Da, RT: 129.25 min,

Identified with: Sequest HT (v1.3); XCorr:4.97

Fragment match tolerance used for search: 1 Da

Fragments used for search: a; a-H_2O; a-NH_3; b; b-H_2O; b-NH_3; y; y-H_2O; y-NH_3

Protein references (1): c.1091C>A p.A364D|LKMDATF|Missense|COSMIC|Lung(1) CANCER_sp_P04350,TBB4A_HUMAN Tubulin beta-4A chain

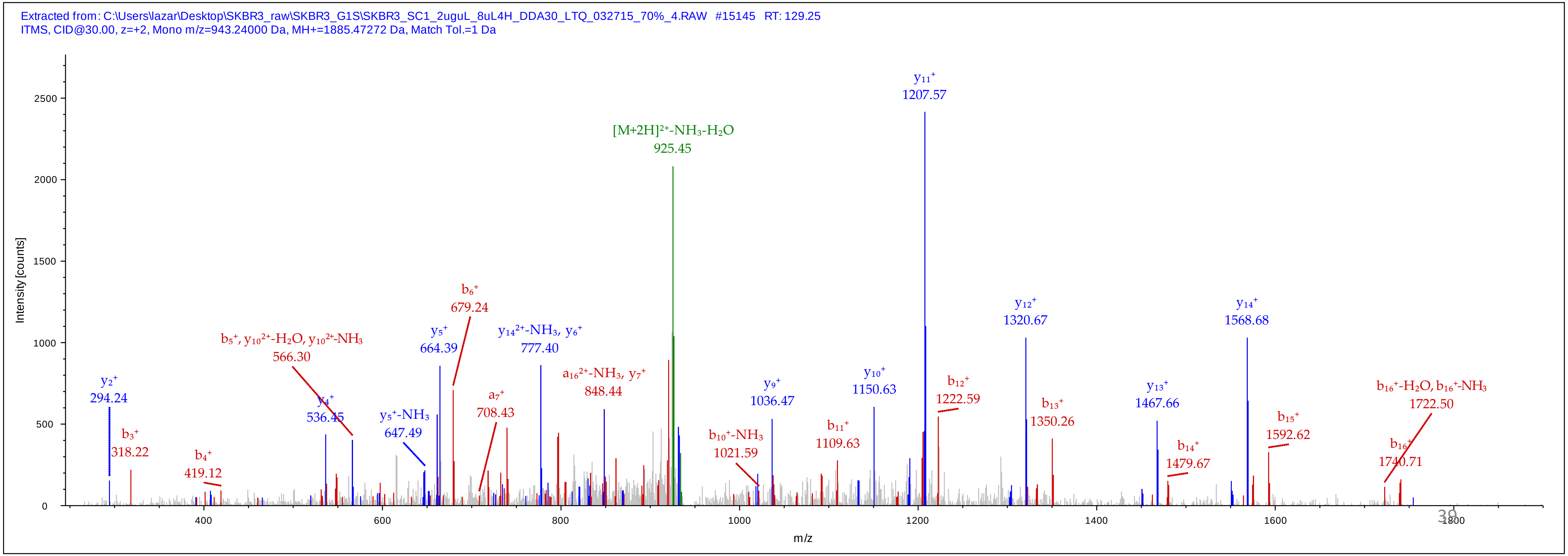

Sequence: MHDMNTDQENLVGTHDAPIR, Charge: +3,   Monoisotopic m/z: 764.80000 Da (-548.32 mmu/-716.94 ppm),   MH+: 2292.38545 Da,   RT: 30.14 min

Identified with: Sequest HT (v1.3); XCorr:4.49

Fragment match tolerance used for search: 1 Da

Fragments used for search: a; a-H_2O; a-NH_3; b; b-H_2O; b-NH_3; y; y-H_2O; y-NH_3

Protein references (1): c.250T>A p.L84M|MHDMNTD|Missense|COSMIC|Skin(2) CANCER_sp_O43684,BUB3_HUMAN Mitotic checkpoint protein BUB3

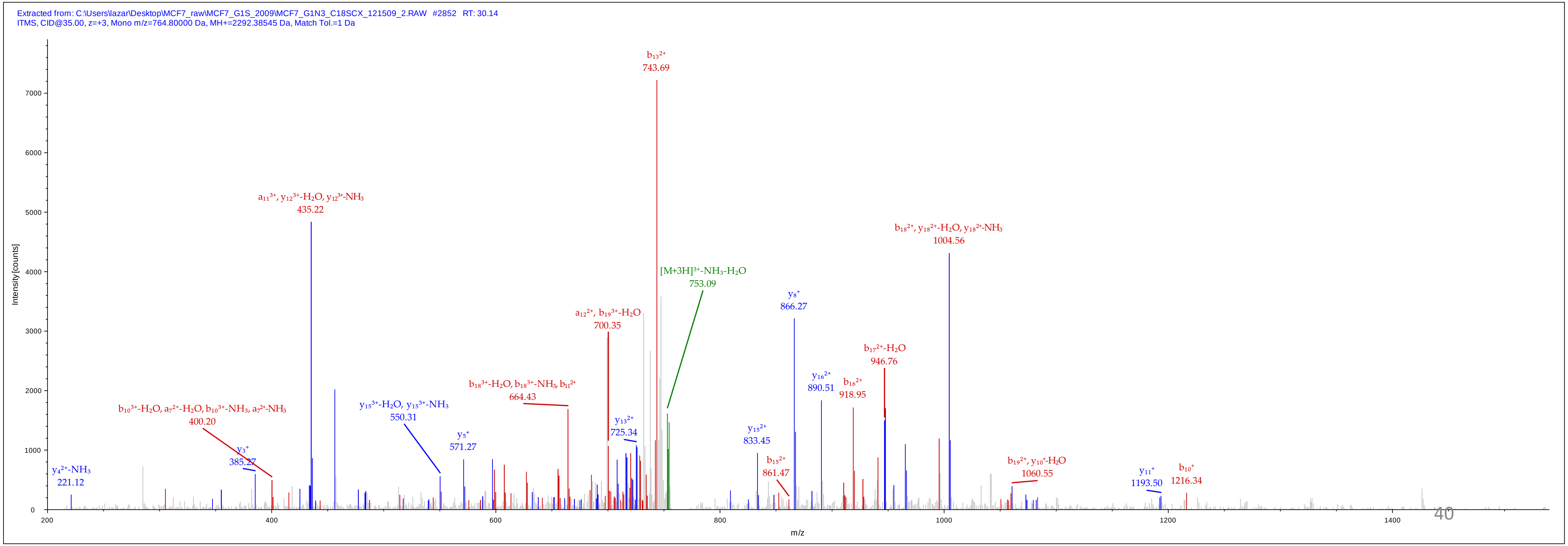

Sequence: MLVVLRQGTREEDDVVSEDLVQQDVQDLYEAGELK, Charge: +3, Monoisotopic m/z: 1341.02000 Da (+684.96 mmu/+510.78 ppm), MH+: 4021.04545 Da, RT: 196.16 min

Identified with: Sequest HT (v1.3); XCorr:5.76

Fragment match tolerance used for search: 1 Da

Fragments used for search: a; a-H_2O; a-NH_3; b; b-H_2O; b-NH_3; y; y-H_2O; y-NH_3

Protein references (1): c.485T>G p.L162R|VVLRQGT|Missense|COSMIC|Liver(2) OR co|ANXA6|c.641T>G|p.L214R|VVLRQGT|Missense|COSMIC|Liver(2) CANCER_sp_P08133,ANXA6_HUMAN Annexin A6

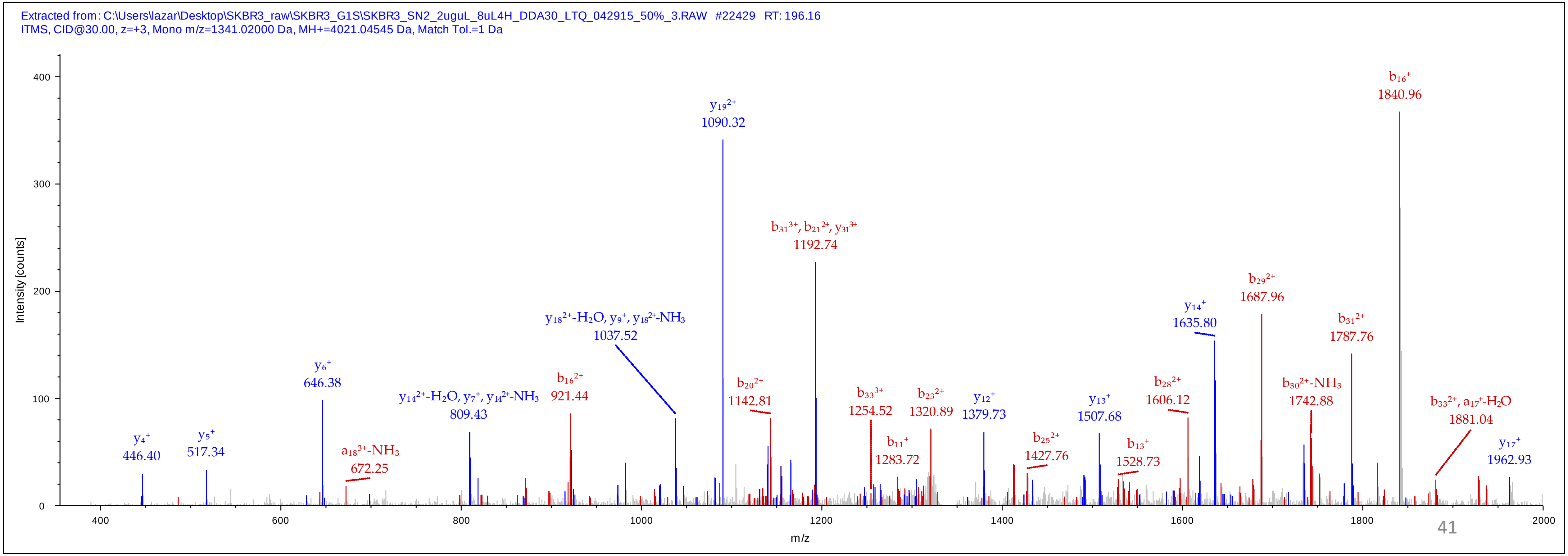

Sequence: QVHPDTGISSKVMGIMNSFVNDIFER, Charge: +3, Monoisotopic m/z: 974.24000 Da (-241.21 mmu/-247.59 ppm), MH+: 2920.70545 Da, RT: 185.78 min

Identified with: Sequest HT (v1.3); XCorr:5.14

Fragment match tolerance used for search: 1 Da

Fragments used for search: a; a-H_2O; a-NH_3; b; b-H_2O; b-NH_3; y; y-H_2O; y-NH_3

Protein references (1): c.176C>T p.A59V|SSKVMGI|Missense|COSMIC|Endometrium(1) CANCER_sp_P23527,H2B1O_HUMAN Histone H2B type 1-O

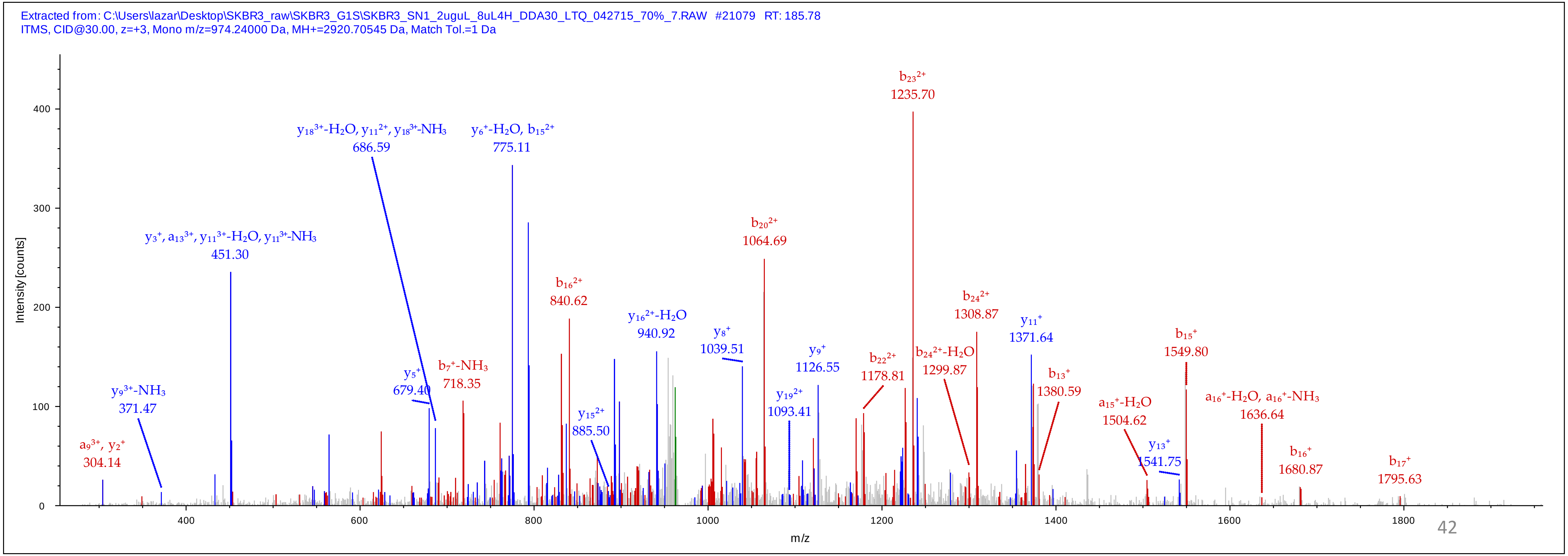

Sequence: QVYPDTGISSKAMGIMNSFVNDIFER, Charge: +3, Monoisotopic m/z: 974.28000 Da (+474.92 mmu/+487.45 ppm), MH+: 2920.82545 Da, RT: 185.86 min
Identified with: Sequest HT (v1.3); XCorr:4.76
Fragment match tolerance used for search: 1 Da
Fragments used for search: a; a-H_2O; a-NH_3; b; b-H_2O; b-NH_3; y; y-H_2O; y-NH_3
Protein references (1): c.148C>T p.H50Y|KQVYPDT|Missense|COSMIC|Haematopoietic and lymphoid tissue(2) OR sp|Q99879,H2B1M_HUMAN Histone H2B type 1-M|c.148C>T|p.H50Y|KQVYPDT|Missense|COSMIC|NS(1) CANCER_sp_O60814,H2B1K_HUMAN Histone H2B type 1-K

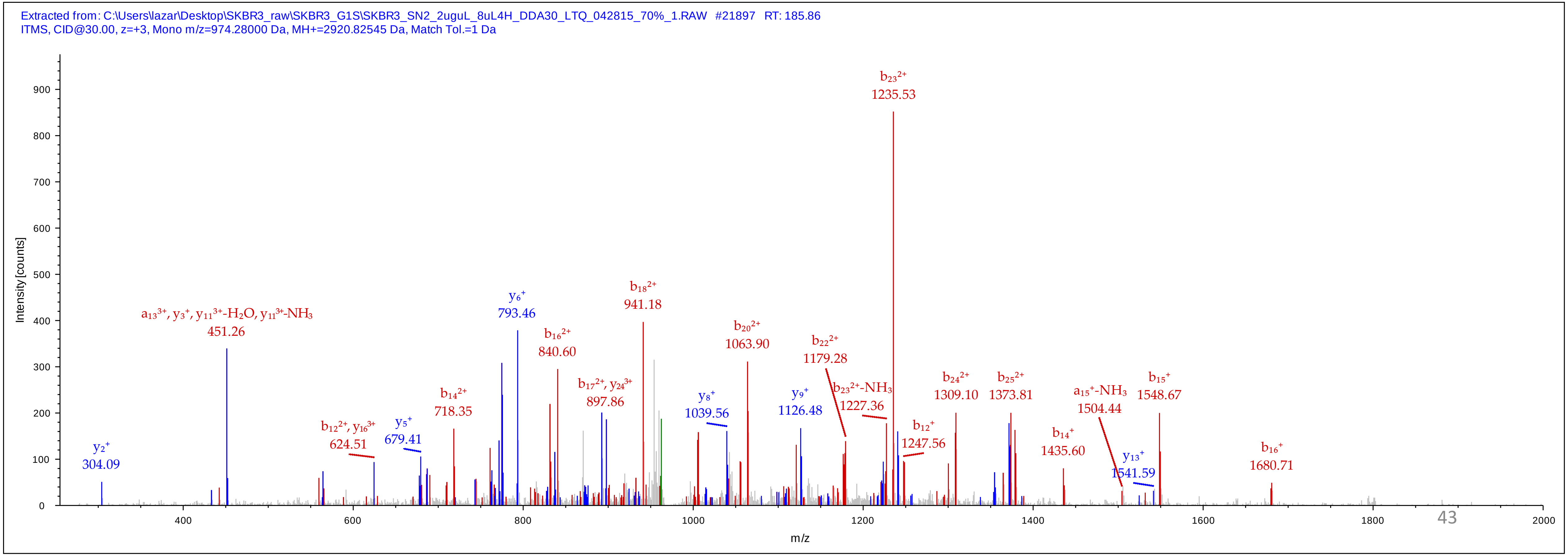

Sequence: SDASSGQSGSRSASRTTR, Charge: +2, Monoisotopic m/z: 899.18000 Da (-245.52 mmu/-273.04 ppm), MH+: 1797.35272 Da, RT: 58.40 min

Identified with: Sequest HT (v1.3); XCorr:4.03

Fragment match tolerance used for search: 1 Da

Fragments used for search: a; a-H_2O; a-NH_3; b; b-H_2O; b-NH_3; y; y-H_2O; y-NH_3

Protein references (2): c.2650C>A p.R884S|DASSGQS|Missense|COSMIC|Lung(1) CANCER_sp_P20930,FILA_HUMAN Filaggrin

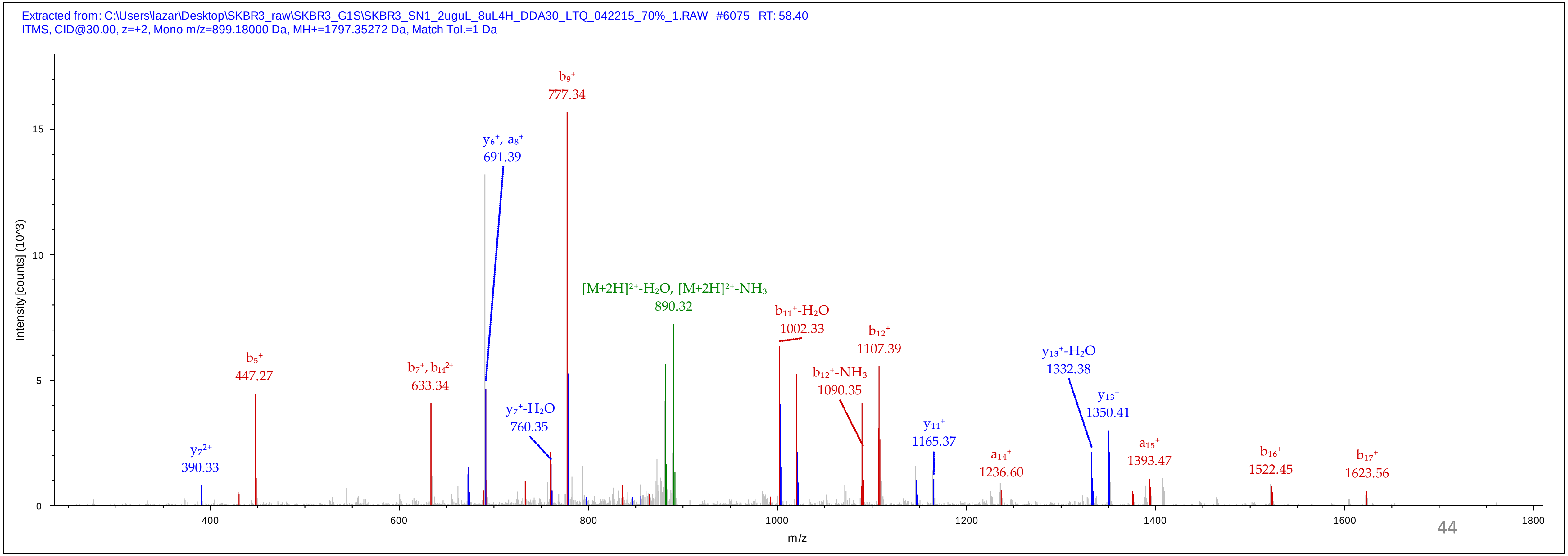

Sequence: SLGQNPTEAELQDMINEVDADGNGTIDFPEFFTMMAR, Charge: +3, Monoisotopic m/z: 1368.98000 Da (+365.01 mmu/+266.63 ppm), MH+: 4104.92545 Da, RT: 158.43 min

Identified with: Sequest HT (v1.3); XCorr:5.17

Fragment match tolerance used for search: 1 Da

Fragments used for search: a; a-H_2O; a-NH_3; b; b-H_2O; b-NH_3; y; y-H_2O; y-NH_3

Protein references (1): c.210G>T p.L70F|PEFFTMM|Missense|COSMIC|Oesophagus(1);Lung(1)

CANCER_sp_P62158,CALM_HUMAN Calmodulin

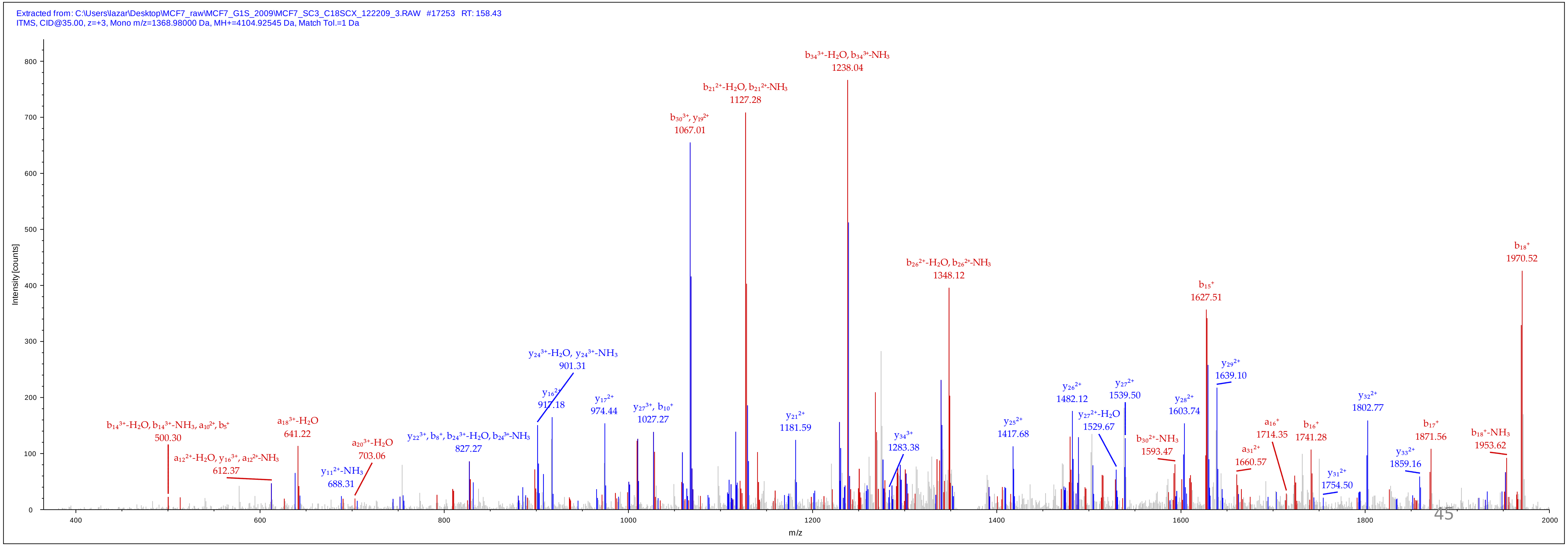

Sequence: SMGIMNSFVNDIFER, Charge: +2,   Monoisotopic m/z: 881.74000 Da (+1329.97 mmu/+1508.35 ppm),   MH+: 1762.47272 Da,   RT: 126.38 min

Identified with: Sequest HT (v1.3); XCorr:4.19

Fragment match tolerance used for search: 1 Da

Fragments used for search: a; a-H_2O; a-NH_3; b; b-H_2O; b-NH_3; y; y-H_2O; y-NH_3

Protein references (1): c.175G>T p.A59S|SSKSMGI|Missense|COSMIC|Lung(1) CANCER_sp_Q8N257,H2B3B_HUMAN Histone H2B type 3-B

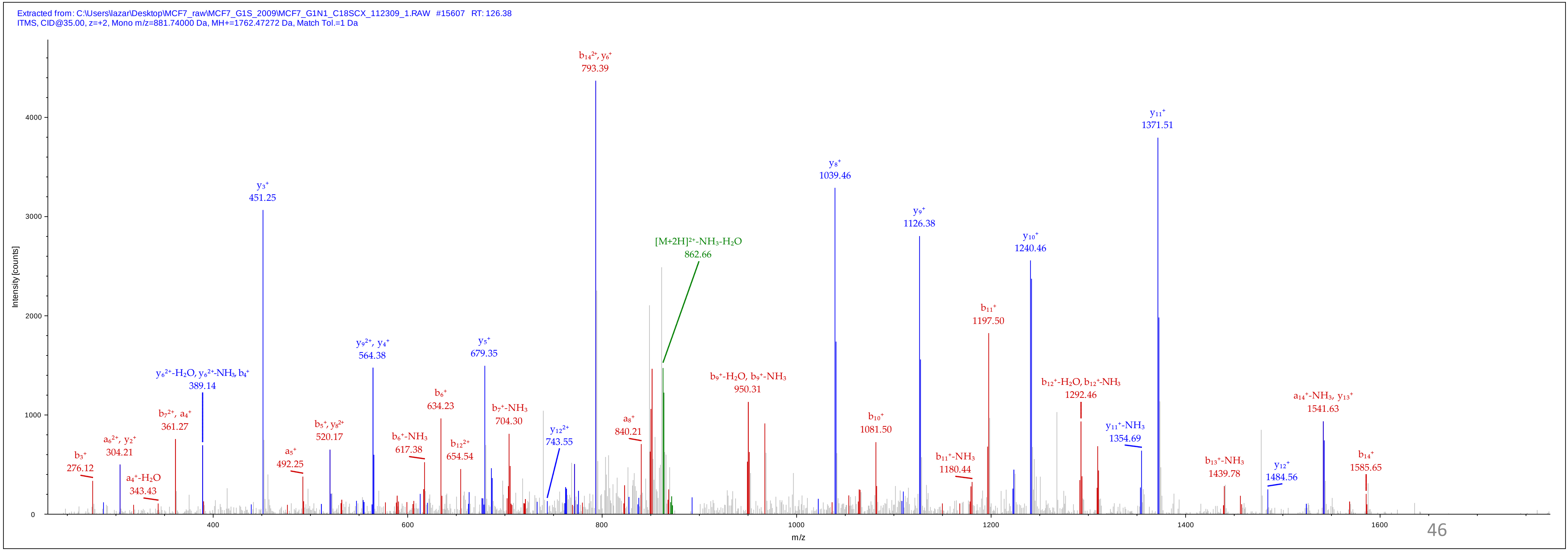

Sequence: SVIVVLRLNVDLQAVVIFELVY, Charge: +2, Monoisotopic m/z: 1251.38000 Da (+139.28 mmu/+111.3 ppm), MH+: 2501.75272 Da, RT: 181.32 min

Identified with: Sequest HT (v1.3); XCorr:3.61

Fragment match tolerance used for search: 1 Da

Fragments used for search: a; a-H_2O; a-NH_3; b; b-H_2O; b-NH_3; y; y-H_2O; y-NH_3

Protein references (1): c.1157delA p.K388fs*39|MQKNLRNQSVAMADWREMKSVIVVLRLNVDLQAVVIFELVY|Deletion - frameshift|COSMIC|Large intestine(4) CANCER_sp_Q8TC27,ADA32_HUMAN Disintegrin and metalloproteinase domain-containing protein 32

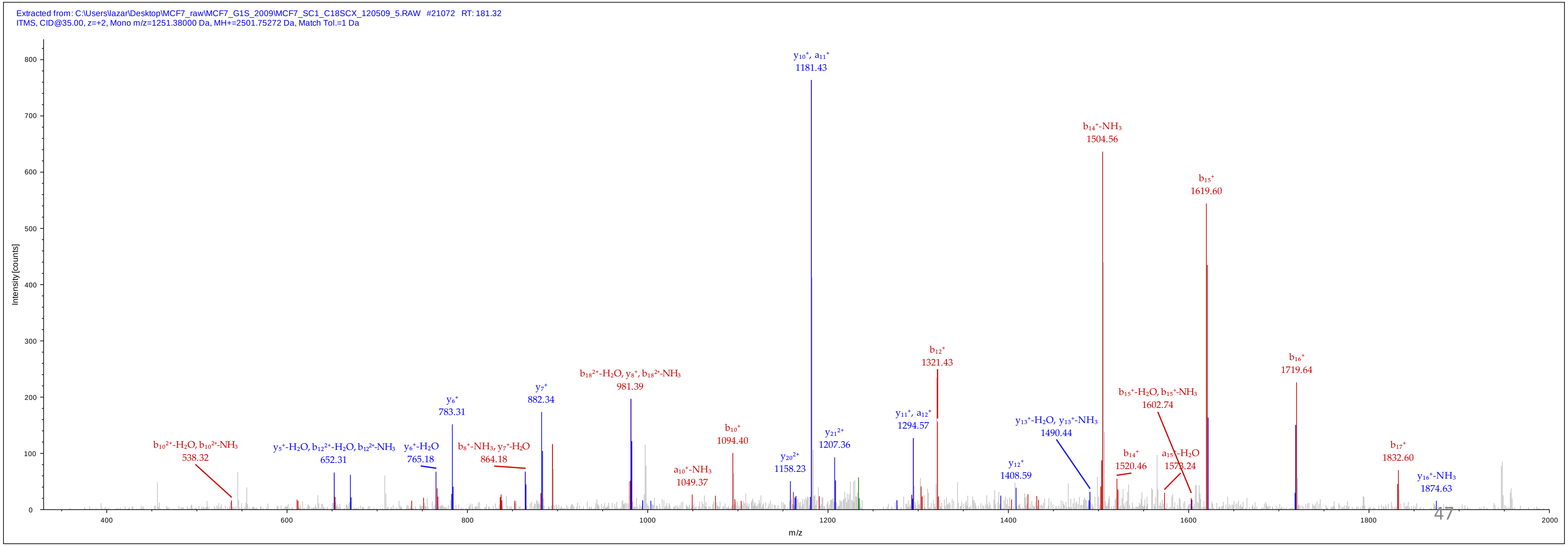

Sequence: SYELPDGQVITIGKER, Charge: +2,   Monoisotopic m/z: 903.38000 Da (+404.57 mmu/+447.84 ppm),   MH+: 1805.75272 Da,   RT: 73.80 min
Identified with: Sequest HT (v1.3); XCorr:4.02
Fragment match tolerance used for search: 1 Da
Fragments used for search: a; a-H_2O; a-NH_3; b; b-H_2O; b-NH_3; y; y-H_2O; y-NH_3
Protein references (1): c.762C>A p.N254K|TIGKERF|Missense|COSMIC|Breast(1) CANCER_sp_P68133,ACTS_HUMAN Actin, alpha skeletal muscle

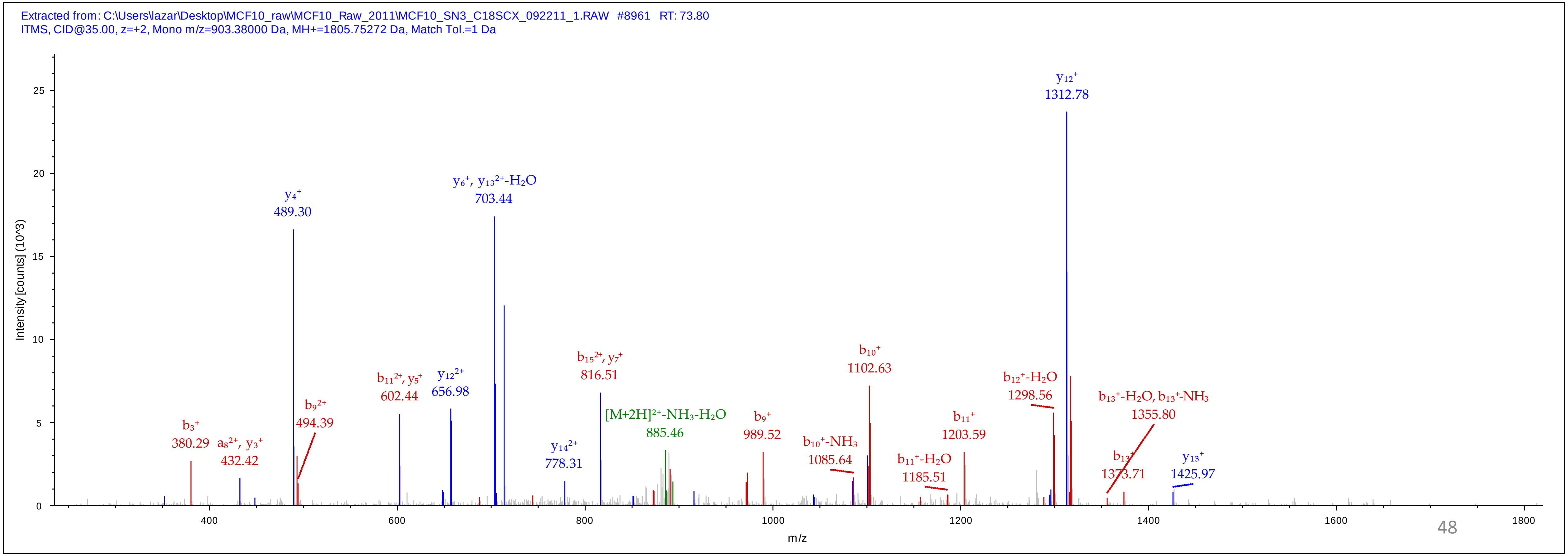

Sequence: TSPDPLPVSAAPSKAGLPR, Charge: +2, Monoisotopic m/z: 932.74000 Da (+1728.35 mmu/+1852.98 ppm), MH+: 1864.47272 Da, RT: 93.95 min
Identified with: Sequest HT (v1.3); XCorr:3.72
Fragment match tolerance used for search: 1 Da
Fragments used for search: a; a-H_2O; a-NH_3; b; b-H_2O; b-NH_3; y; y-H_2O; y-NH_3
Protein references (1): c.989C>T p.S330L|PDPLPVS|Missense|COSMIC|Skin(1) CANCER_sp_Q13469,NFAC2_HUMAN Nuclear factor of activated T-cells, cytoplasmic 2

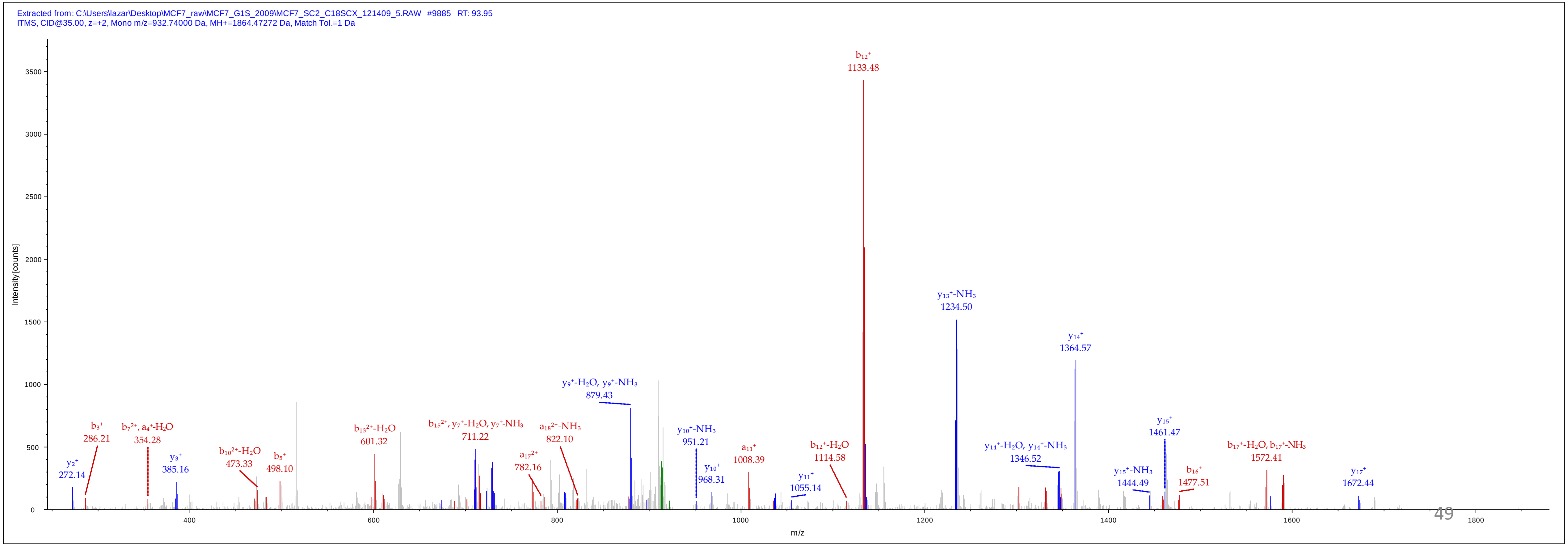

Sequence: TTGIVMDSGDGVTHTVPIYEAYALPHAILR, Charge: +3, Monoisotopic m/z: 1067.04000 Da (+492.09 mmu/+461.18 ppm), MH+: 3199.10545 Da, RT: 104.38 min

Identified with: Sequest HT (v1.3); XCorr:5.94

Fragment match tolerance used for search: 1 Da

Fragments used for search: a; a-H_2O; b; b-H_2O; y; y-H_2O; y-NH_3

Protein references (1): c.503G>C p.G168A|IYEAYAL|Missense|COSMIC|Endometrium(1) CANCER_sp_P63261,ACTG_HUMAN Actin, cytoplasmic 2

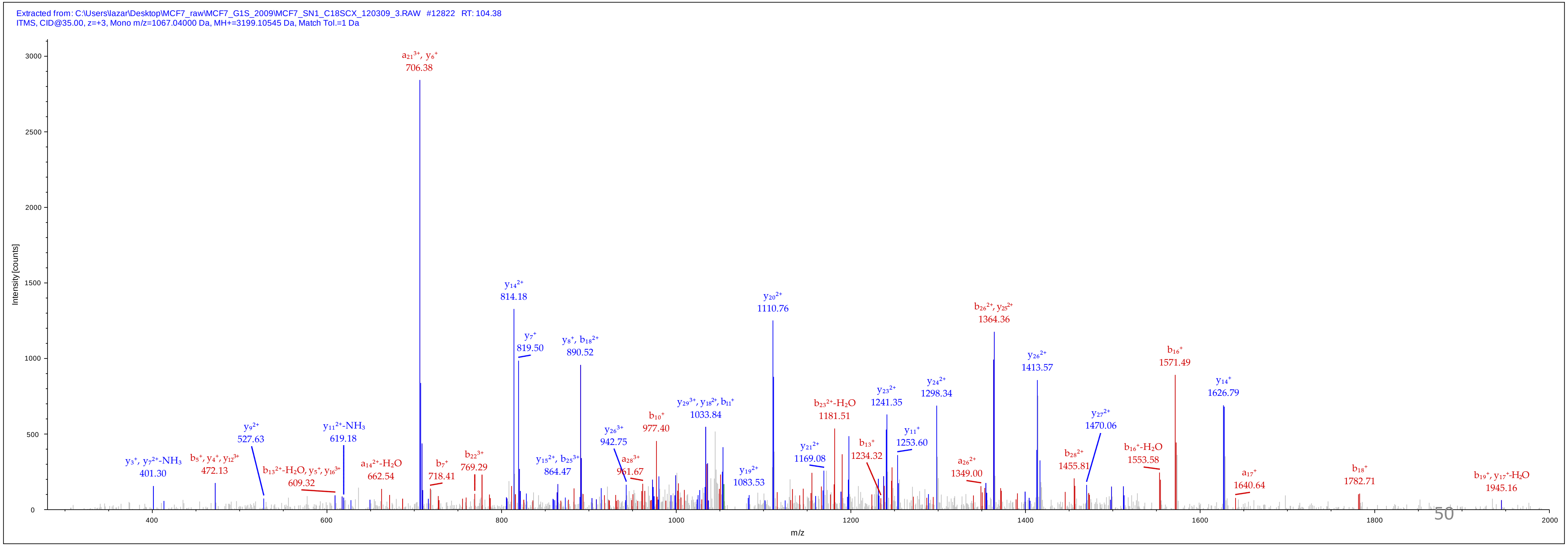

Sequence: TVSLGAGSKDELHIVEAEAMNYEGSPIK, Charge: +3, Monoisotopic m/z: 982.40000 Da (-90.32 mmu/-91.94 ppm), MH+: 2945.18545 Da, RT: 119.62 min,

Identified with: Sequest HT (v1.3); XCorr:4.82

Fragment match tolerance used for search: 1 Da

Fragments used for search: a; a-H_2O; a-NH_3; b; b-H_2O; b-NH_3; y; y-H_2O; y-NH_3

Protein references (1): c.157G>T p.A53S|GAGSKDE|Missense|COSMIC|Lung(1) OR sp|P06748,NPM_HUMAN Nucleophosmin|c.157G>T|p.A53S|GAGSKDE|Missense|COSMIC|Lung(1) CANCER_sp_P06748-3,NPM_HUMAN Isoform 3 of Nucleophosmin

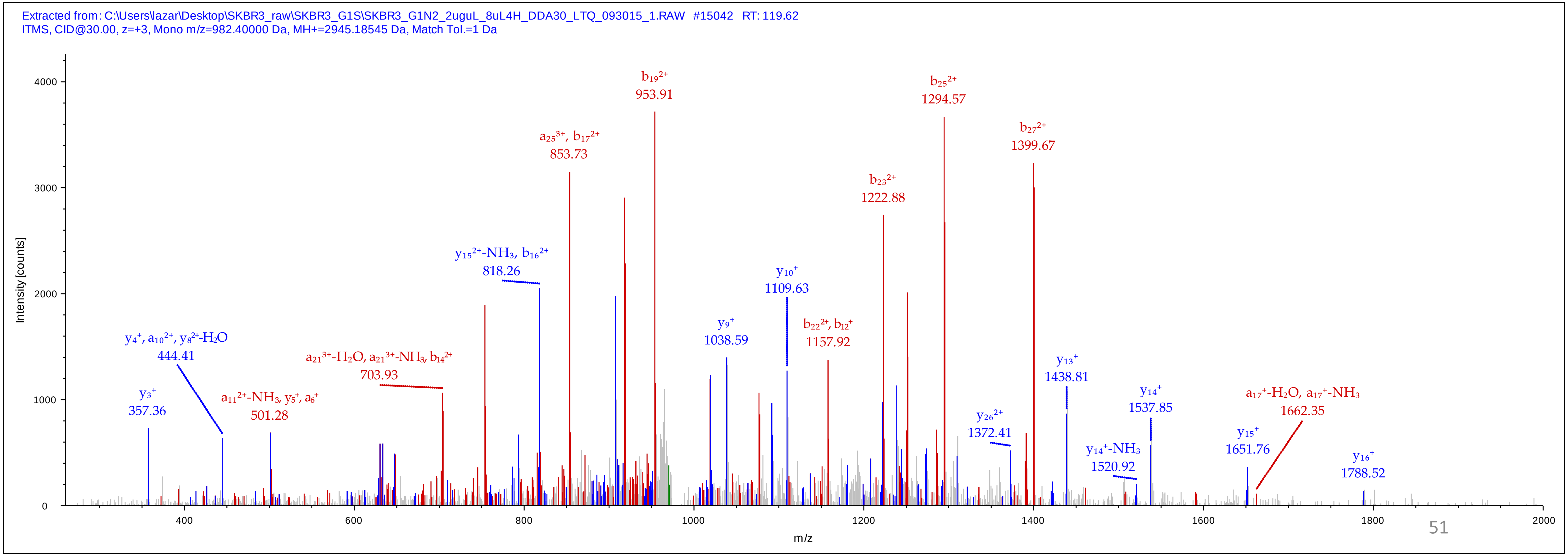

Sequence: VSELEEFINGPNNAHIQQVGDR, Charge: +3, Monoisotopic m/z: 822.34000 Da (-398.81 mmu/-484.97 ppm), MH+: 2465.00545 Da, RT: 72.83 min
Identified with: Sequest HT (v1.3); XCorr:4.03
Fragment match tolerance used for search: 1 Da
Fragments used for search: a; a-H_2O; a-NH_3; b; b-H_2O; b-NH_3; y; y-H_2O; y-NH_3
Protein references (1): c.3564T>G p.D1188E|ELEEFIN|Missense|COSMIC|Endometrium(1) CANCER_sp_P53675,CLH2_HUMAN Clathrin heavy chain 2

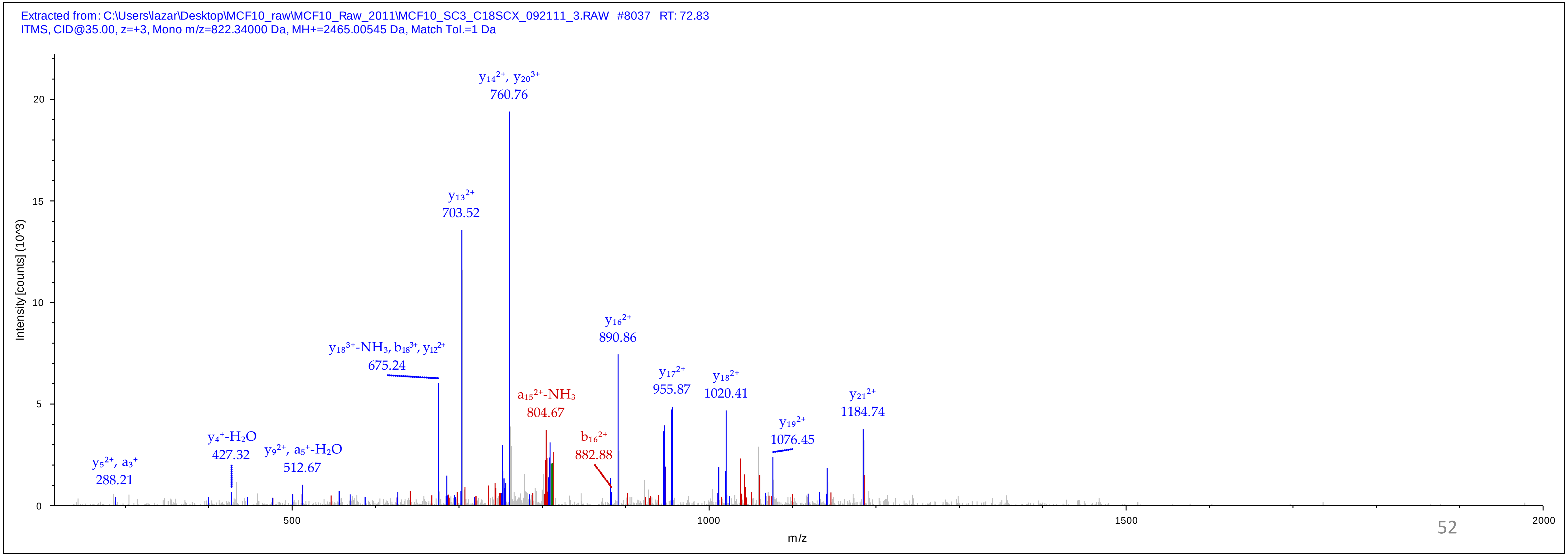

Sequence: VTNGAFTGEISLGMIK, Charge: +2, Monoisotopic m/z: 819.21472 Da (-217.46 mmu/-265.44 ppm), MH+: 1637.42217 Da, RT: 43.66 min
Identified with: Sequest HT (v1.3); XCorr:4.36
Fragment match tolerance used for search: 1 Da
Fragments used for search: a; a-H_2O; a-NH_3; b; b-H_2O; b-NH_3; y; y-H_2O; y-NH_3
Protein references (1): c.242C>T p.P81L|EISLGMI|Missense|COSMIC|Skin(1) CANCER_sp_P60174-1,TPIS_HUMAN Isoform 2 of Triosephosphate isomerase

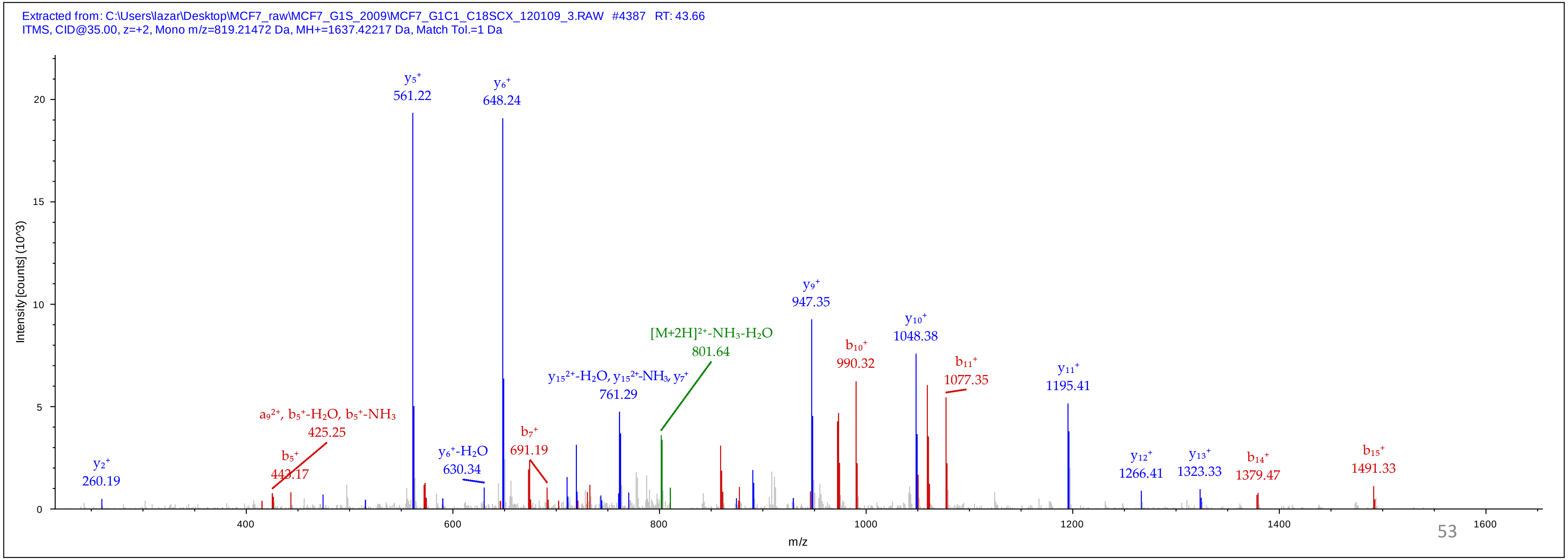

Supplement: Supplementary file 2 — Supplemental Dataset S1 [file 41598_2019_46897_MOESM2_ESM.pdf]
